# Decision-making regarding dental treatments – What factors matter from patients’ perspective? A systematic review

**DOI:** 10.1186/s12903-025-07032-9

**Published:** 2025-11-25

**Authors:** Susanne Felgner, Johannes-Felix Handrock, Carmen Cecilia Schroll, Fabian Schütte, Cornelia Henschke

**Affiliations:** 1 Department of Health Care Management, Berlin Centre of Health Economics Research (BerlinHECOR), Technische Universität Berlin, Berlin, Germany; 2 Institute of Gender in Medicine, Charité – Universitätsmedizin Berlin, Berlin, Germany; 3 Institute of General Practice and Interprofessional Care, University Hospital Tübingen, Tübingen, Germany

**Keywords:** Dental care, Patient, Access, Decision-making, Factors of choice, Out-of-pocket payment, Dental fear, MMAT, COVID-19, SARS-CoV-2

## Abstract

**Background:**

Achieving oral health for the population should be a concern of public health care systems, as it may affect their expenditures in the long term. Patients often face individual challenges in dental care. Why patients decide for or against dental treatments can be determined by many factors, e.g., their own financial resources, preferences, and external circumstances. This cross-country study aims to identify those factors.

**Methods:**

We systematically searched for literature in the biomedical databases PubMed (including MEDLINE), the Cochrane Library, and Web of Science to identify factors influencing dental treatment decisions across different countries. Factors of choice were extracted from relevant articles to develop a codebook for subsequent qualitative analysis using an inductive thematic analysis approach. Study quality was assessed using the Mixed Methods Appraisal Tool (MMAT). This systematic review followed the guidelines of the Preferred Reporting Items for Systematic reviews and Meta-Analyses (PRISMA) and the Synthesis Without Meta-analysis (SWiM) statements.

**Results:**

After multistage screening of *N* = 4,226 publications by two reviewers, *N* = 233 relevant articles of different study designs (qualitative (*N* = 42), quantitative (*N* = 177), and mixed-methods (*N* = 14)) were included in the analysis. Data collection was realized across different settings (e.g., dental practices (*N* = 18)) and approaches (e.g., interviews) in 49 countries. Included articles focused on specific treatments (e.g., caries treatment) or treatments in general (e.g., dental tourism). Across the countries, various factors of choice (*n* = 101) were identified, divided into three categories: (I) "Dentist & dental institution" (e.g., communication), (II) "Patient" (e.g., dental fear), and (III) "Treatment" (e.g., durability). The factors 'out-of-pocket payment' and 'dental fear' were identified in most of the articles (*N* = 136, *N* = 64) and were mentioned most frequently (code frequencies: *n* = 151, *n* = 73). In countries with the most articles (e.g., the UK (*N* = 28), Saudi Arabia (*N* = 23), the USA (*N* = 22), India (*N* = 19), and Brazil (*N* = 14)), also 'out-of-pocket payment' was identified most often (e.g., the UK: in 56% of the articles; India: 68%). Frequency of the factor 'dental fear' varied by country. One publication addressed the COVID-19 pandemic. It reported that treatment appointments were postponed and canceled by patients due to their fear of infection with SARS-CoV-2. The quality of the included studies varied considerably.

**Conclusions:**

A range of factors influence patients’ choice regarding dental treatments. Understanding patients’ motivation for seeking dental care can guide the development of interventions (e.g., awareness campaigns and health literacy efforts) that support proactive dental care. To improve oral health outcomes and reduce access barriers, tailored regulatory and informational strategies are essential.

**Supplementary Information:**

The online version contains supplementary material available at 10.1186/s12903-025-07032-9.

## Background

Oral health can affect many areas of human life [1]. It affects the overall health of people, their quality of life, and work productivity [2, 3], and thus also has economic effects [4–6]. Untreated dental diseases can lead to health issues such as mental or coronary diseases [7, 8], which may result in increased demand for medical treatment and associated health care costs.

Oral diseases are a global public health issue. In 2019, they accounted for an estimated US$387.09 billion in direct costs and US$322.69 billion in indirect costs worldwide, with highest expenditures reported for the USA and Germany, as a country of Western Europe [9], while further increases are expected [10]. Nevertheless, the amount of expenditures may still be underestimated, as dental care can be associated with (high) costs for patients [11]. Understanding why patients may or may not utilize dental care is therefore crucial not only to improve individual health outcomes, but also to inform health policies that aim to reduce the economic burden of untreated oral diseases. Targeted efforts to reduce access barriers and increase preventive care are critical for minimizing the progression of oral diseases and their broader economic impact.

Overall, coverage of dental services by public health care systems varies both across and within countries, e.g., partial coverage of prosthetic treatment in Austria and Germany, but no cost coverage for fixed prosthetics in Belgium [12, 13]. Although access to basic dental care is available in many countries and may appear barrier-free, not all patients take advantage of dental visits. As multiple studies from different countries have shown, a variety of factors can influence dental care utilization [14–16]. However, one decisive factor could be that as soon as dental services go beyond a certain level of care, out-of-pocket payments may arise for patients. These additional costs may be unaffordable for patients with limited financial means, thus deterring them from seeking necessary dental services [17, 18].

In addition, patients may underestimate the importance of oral health unless they experience pain or face an acute dental emergency [19]. Accordingly, preventive measures are taken only sporadically or not at all. Prior negative experiences and dental anxiety can further discourage individuals from seeking care [20, 21]. As dental care is one of the areas most affected by respiratory illnesses such as COVID-19 [22], patients may have been additionally avoiding dental visits due to their fear of infections since the beginning of the pandemic. For example, for medical disciplines including cardiology and cancer medicine, it was reported that patients postponed or even canceled screenings and necessary treatments during the pandemic [23, 24]. Globally, factors of choice for (not) utilizing dental services from patients’ perspective can be diverse and represent combinations of factors due to country-specific characteristics (e.g., cultural or religious aspects [25, 26]). Despite the broad range of factors influencing patients’ decision-making, there is still limited systematic understanding of how these factors interact across different national and health system contexts.

In 2021, the World Health Organization (WHO) resolution on oral health called on member states to strengthen oral health service delivery. It should be part of the essential health services package to provide universal health coverage. Member states are encouraged to consider measures such as promoting the integration of oral health into national health policies and strengthening prevention strategies. Based on a draft global strategy on tackling oral diseases, an action plan for public oral health was developed to be achieved by 2030 [27]. In the context of the COVID-19 pandemic, shifting health priorities, and increasing awareness of patient-centered care, understanding patients’ treatment decisions has become increasingly important. While previous studies have explored dental service utilization, a comprehensive international overview of patients’ decision-making factors is still lacking. Knowing the reasons that drive patients to seek dental care is valuable when deciding on interventions to improve population oral health. To address this gap, the present study provides a structured overview of the factors influencing patients’ dental treatment decisions.

This international systematic review identifies and synthesizes factors of choice and country-specific peculiarities that may be crucial for tailoring treatment options and designing patient communication. Insights can serve as an important information source for health policy makers, insurers, and dentists, in developing strategies to enhance patient satisfaction, accessibility to, and utilization of appropriate dental services. Moreover, this study supports dentists and dental professionals in making their practice more patient-centered and evidence-based by providing clarity on patients’ preferences. Therefore, the aim of this study is to systematically identify and analyze factors influencing patients’ decisions for or against dental treatments across different countries. Specifically, we address the following research question: "What factors influencing patients’ decision-making on dental treatments can be identified internationally?".

## Main text

### Methods

#### Systematic literature search and PICO scheme

For the literature search process, we followed relevant steps recommended for the preparation of health technology assessment (HTA) reports [28]: (1.) transformation of the research question into an answerable question using the PICO scheme, (2.) preparation of a research concept, (3.) identification of synonyms, (4.) selection of relevant information sources, (5.) development of search strategies, and (6.) conducting of searches in databases and (7.) a supplementary literature search.

Referring to our PICO scheme (Table 1), developed in step (1.), the population (P) consists of (potential) patients in dental care, including parents or caregivers (e.g., of children/adolescents or elderly people) making treatment decisions, and excluding patients with special needs in dental care (e.g., people with diabetes or HIV), with freedom of choice regarding dental treatments. The intervention (I) is dental treatments, including dental care and services. No comparator intervention (C) was defined. Outcomes (O) were patients’ factors of choice regarding the utilization of dental treatments. The study design (S) comprised primary studies, including comparative and non-comparative studies (qualitative, quantitative, and mixed-methods studies), considered in the full-text screening, and excluded secondary studies such as (systematic) reviews and other publication types (e.g., summaries, comments, study protocols). However, the items 'outcome' and 'study design' were not considered in the literature search process to avoid repetition (covered by (P)) and to ensure a sensitive search (regarding (S)). Although the outcome ("factors of choice") was not used as a search term, we ensured its inclusion conceptually by incorporating the related terms "determinant(s)" and "(patient) preferences" into the search strategy. These terms were derived from a preliminary scan of the relevant literature and reflect how the concept of patients’ choice is typically operationalized in existing research [29–31]. The search terms were part of a predefined strategy developed prior to querying the databases. A guideline on the literature search, study selection, and data analysis, as well as the previously developed search strategy, can be found as additional files (A1. Guideline on literature search, selection, and analysis; A2. Search strategy).

**Table 1:** PICO scheme, including search items

PICO item			(Search) item
P	–	Patient/problem	(Potential) patients with freedom of choice
I	–	Intervention	Dental treatment
C	–	Comparison	-
O	–	Outcome*	Patients’ factors of choice regarding the utilization of dental treatments
S	–	Study design*	Primary studies, including comparative and non-comparative studies (qualitative, quantitative, and mixed-methods studies)
T	–	Time period	10 years

^*^Item not considered in the literature search process

The research concept was built using blocks (2.) (Table 2). Synonyms for the search were identified by screening thematically relevant literature and websites (3.). We searched the biomedical databases PubMed (including MEDLINE), the Cochrane Library, and Web of Science (4.). Search strings were developed for each database in step no. five (5.). Two individual literature searches were conducted (6.): an initial search and an update search. The publication period of the initial search was from 2007 to 2018. The update search was conducted to cover the period from 2019, ensuring continuity with the initial search, through to January 2021 (7.). A protocol for this systematic review has been registered and published in the PROSPERO database (CRD42021276494).

**Table 2:** Research concept, including PICO and search item, and Boolean operators

PICO item P	Boolean operator	PICO item I	Boolean operator	PICO item T
Patient AND factors of choice	AND	Dental treatment	AND	10 years + update

#### Criteria and selection

The screening process was conducted in multiple stages. Duplicate entries were removed from the literature pool. Subsequent data selection included (I) a title/abstract (TiAb) and (II.1&2) a two-step full-text screening. Additionally, (III) reference lists of identified systematic reviews were searched by titles. For (I) the TiAb screening applied inclusion criteria focused on patients’ factors of choice and dental treatments. No exclusion criteria were defined for the TiAb screening. The full-text screening was performed in two steps: (II.1) the application of exclusion criteria (see Table 3), and (II.2) the "eligibility screening" [32], which involved evaluating whether the study addressed the research question in a meaningful way. For this purpose, we screened the discussion and conclusion sections of the articles to determine whether relevant findings on patients’ decision-making in dental care were reported. Both full-text screening steps were conducted independently by two reviewers. In cases of disagreement, a consensus was reached through discussion between them. Studies not available in English or German were excluded (E2) due to limitations in translation resources and to ensure accurate interpretation of findings. Studies focusing on patients with special needs (E4) were excluded to maintain a consistent population scope, as their treatment decisions may be influenced by condition-specific medical factors not representative of the general patient population.

**Table 3:** Literature exclusion criteria and abbreviations

Abbreviation	Exclusion criterion
E1	No full-text
E2	No full-text in English or German
E3	No qualitative or quantitative primary study
E4	Patient(s) with special needs
E5	No dental treatment(s)
E6	No patients’ factors of choice or preferences
E7	Multiple publication without relevant additional information

#### Data extraction, identification of factors of choice, and quality assessment

A standardized data extraction sheet was developed to extract the following items: study characteristics (e.g., country of study conduction, name of dental treatment, sample size, gender distribution), methodological details, and articles’ findings on patients’ factors of choice. For reasons of clarity and comparability, the extracted data were categorized into thematic groups, some of which showed a wide range of expressions within certain categories (e.g., income of study participants). For example, regarding the category "income", we only extracted income data for the group with the largest number (or proportion) of participants and classified this group as "low", "middle", or "high" according to the ranking in the respective article. Categories were defined pragmatically, based on the most frequently reported variables and those that allowed for meaningful synthesis across studies. For example, in Furnham et al. [33] the highest share of participants (24%) fell into the £15.000–22.000 income group, which was the second lowest of six groups. Accordingly, this was classified as "low" income. Similarly, we proceeded with the participant characteristics "education" (classified as: "school education" and "university or college degree") and "oral health status" (classified as: "(very) good" and "fair or poor"). While this approach ensured comparability across studies with heterogeneous reporting formats, it is important to acknowledge that country-specific definitions of these categories may limit direct cross-country interpretation, a point further elaborated in the *Discussion* section. For "age" we extracted the mean/median age or the age group with the most participants, depending on data availability. Types of treatment ("dental treatment or service") were grouped into three categories (i) "dental treatment", (ii) "dental service in general", and (iii) "dentist & dental office", following the categorization in a previous study [34]. Missing data were marked with "ns" (not stated or unclear) and were not imputed.

Data extraction was prepared by the reviewers SF and JFH, and was finalized by SF. SF and JFH independently extracted the factors of choice. After joint discussion, SF and JFH sorted and summarized these factors according to similar contents, resulting in a codebook as the basis for a subsequent qualitative analysis, which was conducted using ATLAS.ti (version 9).

#### Data analysis and presentation of results

Qualitative analysis was conducted using an inductive approach and thematic analysis to generate themes emerging directly from the data, without a predefined framework [35, 36]. To analyze the identified factors of choice based on the developed codebook, text passages from the included articles reporting study findings were coded by two reviewers (SF and JFH), with multiple coding applied when passages reflected more than one aspect of patients’ decision-making. For the coding process, the ATLAS.ti tool "inter-coder agreement analysis" was used. Krippendorff’s alpha was calculated to assess the inter-coder agreement (ICA), with α ≥ 0.80 defined as the threshold for high reliability [37]. To achieve this, SF and JFH initially discussed the coding scheme to ensure a shared understanding of all code definitions. The material was then coded independently through an iterative process until the target α level was reached, ensuring both consistency and methodological rigor. Discrepancies between the reviewers were resolved through discussion and consensus. After finalizing the code set, similar codes were grouped into overarching themes to identify key patterns across the studies [35, 36].

For quality assessment of the studies reported in the included articles, we used the Mixed Methods Appraisal Tool (MMAT) in tabular form. The MMAT is designed to critically assess different study designs: (I) qualitative studies and quantitative studies, including (II) randomized controlled trials (RCTs), (III) non-RCTs, (IVa) descriptive cross-sectional and (IVb) longitudinal studies, and (V) mixed-methods studies [38, 39]. Using two introductory assessment questions, S1 and S2, articles to be included for further assessment were identified. The subsequent five questions (response options: yes = 1 and no = 0 "points") were adapted to the respective study design [40]. The points of the five assessment questions were then summed up for each article ("number of points") and used to calculate an article’s ratio ("quality score": 0.0–1.0). A higher MMAT quality score indicates a higher study quality [32]. For example, as part of the assessment, we screened the title, introduction, and methods section of each article for keywords (e.g., "randomization" in RCTs, "content analysis" in qualitative studies). The quality scores are reported descriptively and critically discussed. However, they were not used to influence study inclusion, weighting, or interpretation of findings. The purpose of this quality appraisal was to provide transparency about the methodological rigor of the included studies. The MMAT was applied systematically and consistently across all studies. Study quality for articles from the initial search was assessed independently by two reviewers (SF and CCS). The MMAT results were then compared, and any discrepancies between the reviewers were discussed in detail and resolved through consensus. Since SF was to assess study quality for the update search alone, inter-coder reliability (ICR) was determined based on the articles from the initial search. A Cohen’s kappa value of κ ≥ 0.61 was considered indicative of substantial agreement [41]. If this threshold was not reached, a minimum sample from the update search was to be selected for a cross-check and ICR calculation [41]. This procedure was necessary due to a change in reviewers regarding the update search articles (SF and JFH), which resulted from capacity constraints. If the κ level was not met, additional quality assessment rounds with both reviewers were to be conducted, each followed by a discussion of discrepancies, until substantial agreement was achieved. Remaining differences were then resolved through consensus.

For a transparent report of our systematic review, we used the Preferred Reporting Items for Systematic reviews and Meta-Analyses (PRISMA) guideline [42] and its extension: the Synthesis Without Meta-analysis (SWiM) reporting guideline [43]. The most recent versions of the tools were applied: the MMAT 2018, PRISMA 2020, and SWiM 2020. Documentation based on PRISMA and SWiM can be found in the additional files (A3. PRISMA checklist; A4. SWiM checklist). However, while study quality was assessed using the MMAT, a formal GRADE [44] assessment for the certainty of evidence for the synthesized findings was not performed. The primary objective was to identify and synthesize factors rather than to quantify and grade certainty of specific intervention effects.

## Results

### Selected articles and their characteristics

**Fig. 1:**
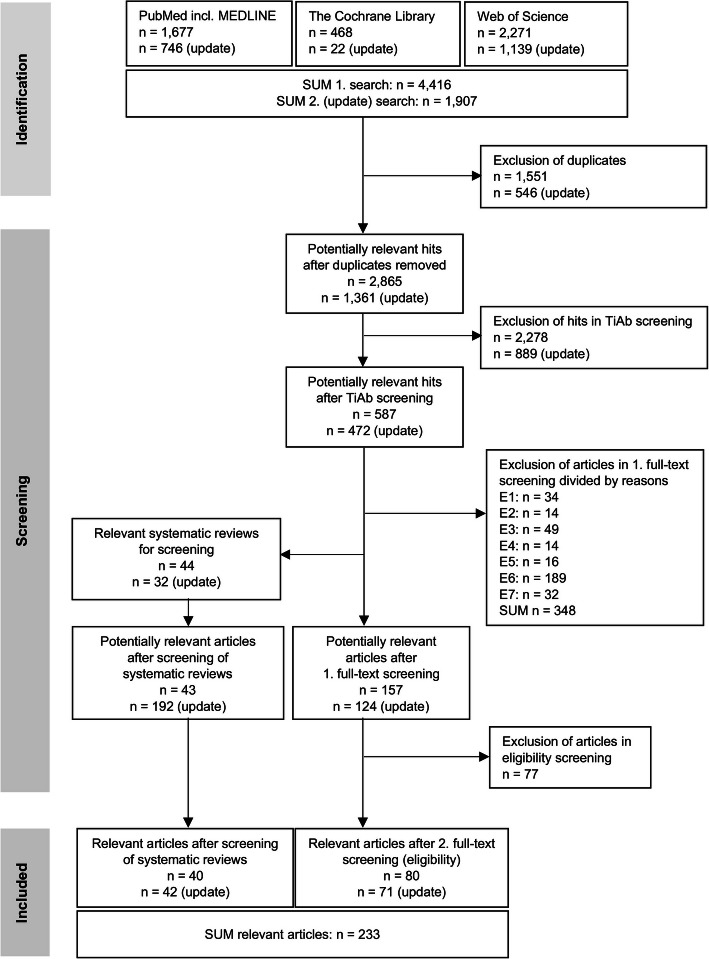
Flow chart of the literature search in databases and the screening process; n – number of hits/articles, TiAb – title/abstract, E1 – no full-text, E2 – no full-text in English or German, E3 – no qualitative or quantitative primary study, E4 – patient(s) with special needs, E5 – no dental treatment(s), E6 – no patients’ factors of choice or preferences, E7 – multiple publication without relevant additional information

A total of *N* = 6,323 hits (articles) were identified from the database searches (initial: *N* = 4,416; update: *N* = 1,907). The total number of relevant articles identified was *N* = 233 (*N* = 230 journal articles, two institutional reports [45, 46], and one master’s thesis [47]), including *N* = 42 qualitative, *N* = 177 quantitative (*N* = 8 RCTs, *N* = 9 non-RCTs, *N* = 160 descriptive studies (*N* = 156 cross-sectional, *N* = 4 longitudinal)), and *N* = 14 mixed-methods studies. An overview and the selection process of the identified literature in accordance with the PRISMA criteria [48] is given in the flow chart in Fig. 1. In addition, the search strings and hits in the databases are listed in an additional file (A5. Search strings for databases, including hits).

We identified articles on studies conducted in 49 different countries, with most articles from the UK (*N* = 28), Saudi Arabia (*N* = 23), and the USA (*N* = 22). Only one article per country was given for 19 countries, e.g., Poland [49], Greece [50], and Mexico [51]. A few studies (*N* = 5 articles) included participants from multiple countries, such as reported in Bucchi et al. [52] (Portugal, Chile, Spain, France, and Italy) and Ellis et al. [53] (the UK and Canada). Some articles could not be clearly assigned to certain countries, as they either reported on (online) studies with an international focus (*N* = 2, [54, 55]) or the place of conduction was not clearly stated (*N* = 9, e.g., [56, 57]). The location and the health sector considered (public: *N* = 29, private: *N* = 16, or both: *N* = 28) varied across the articles. We distinguished between dental practice (*N* = 18); (dental) clinic/hospital (*N* = 79) (e.g., in waiting areas [58, 59]); academic institution (*N* = 25) (e.g., dental department at a university [60] or college [61]); dental service institution (*N* = 7) (e.g., public health service of a city [62]); other non-private areas (*N* = 22) (e.g., school [63, 64], grocery store [47]); and in-private settings (*N* = 39) (e.g., at home [65, 66]). In four articles, the focused medical facility was an emergency department [67–70]. Due to the different study designs, various data collection instruments were used, including in-depth and focus group interviews, questionnaires (self-, interviewer-, dentist-administered, etc.), including surveys with scales (e.g., Visual Analogue Scale (VAS) [71, 72]), but also online searches (e.g., searches for blogs [54] and forum posts [73]), and combinations of approaches [55, 62].

Study populations ranged from a few participants, e.g., *n* = 2 in a case study [51], to *n* = 40,305 participants in a comprehensive questionnaire survey [74]. Most participants were interviewed directly (*N* = 214), but in some cases, parents or caregivers were consulted on behalf of their children (*N* = 24) (e.g., [75, 76]) or elderly people (*N* = 2, [77, 78]). Accordingly, age varied from young infants (e.g., 2 years of age [79–81]) to age groups with a high maximum age (e.g., 101 years [82]). In most articles, the proportion of female participants predominated (*N* = 137). In some articles, it was as high as 100% (*N* = 5), also due to the research questions (e.g., investigation of maternal beliefs and motivations for children’s first dental visit [76]). In terms of further sociodemographic characteristics, education levels of adult respondents ranged from elementary school education (e.g., [83]) to university degree and above (e.g., [84]), and income varied from "low" to "high", while study participants with low and middle (household) income predominated ("low": *N* = 22, 39% of all articles reporting this characteristic; "middle": *N* = 21, 37%). Overall, in less than half of the articles, data on participants’ education (*N* = 107, 46%) and income (*N* = 74, 32%) were reported. This limited reporting of sociodemographic data in a significant portion of included articles constrains a more granular understanding of how these characteristics might modulate patients' factors of choice across diverse contexts and should be considered when interpreting the generalizability of findings related to these demographics. Furthermore, the articles focused on various aspects of dental care, as can be seen when we grouped the articles into the defined categories according to the types of treatment for which factors of choice were identified: (i) "dental treatment" (*N* = 138), describing a single treatment (e.g., implant overdenture [85], teeth whitening [86]); (ii) "dental care in general" (*N* = 68), including the entirety of dental treatments (e.g., oral health care of patients [51], utilization of dental services [15]); and (iii) "dentist & dental practice" (*N* = 27) (e.g., appearance of the dentist [63], organization [87], (technical) equipment of dental practice [88]).

In the additional files (A6. Characteristics, factors of choice, and references of included articles (*N* = 233)), extracted study characteristics (i.e., information on study setting (e.g., country) and population (e.g., age, income), and treatment category), as well as the factors of choice and the articles’ references, are listed by study design. Moreover, details on the articles’ methods can be found in the additional files (A7. Methodological characteristics of included articles (*N* = 233), and search details (e.g., recruitment, in-/exclusion criteria, incentives, source of literature)).

### Findings of studies

#### Factors of choice

Several different factors of choice could be identified. Overall, some of these were identified only once (e.g., 'dentist smell' [89], (patient’s) 'forgetfulness' [16]) or in a few articles (e.g., 'chewing ability'/'function': *N* = 7, e.g., [90, 91]), while others were mentioned in several articles. With *N* = 136 articles, 'out-of-pocket payment' (and its synonyms; e.g., 'cost') is the factor that was mentioned in most articles (e.g., [67, 92, 93]), followed by 'fear' (of treatment) (*N* = 64, e.g., [68, 94]; synonyms: e.g., 'fear of needles'), 'aesthetics' (*N* = 53, e.g., [95, 96]; synonyms: e.g., 'cosmetic'), and 'pain' (*N* = 49, e.g., [72, 97]; synonyms: e.g., 'pain intensity').

The codebook contained *n* = 176 codes as a framework for the qualitative analysis. There was a total of two coding runs, as the ICA target value was not reached after the first one (α = 0.683). In the second run, the value was an acceptable α = 0.865. After the coding runs, some codes were excluded (e.g., if they were merged with other codes, meaning the same factors). Also, some codes were split because they reflected several factors. This was followed by a final consensus discussion, resulting in *n* = 187 codes representing factors of choice. Subsequently, codes with the same content were summarized thematically, and a suitable term was assigned by the reviewers in a discussion. This resulted in *n* = 101 factors, which were first assigned to nine subcategories and then to the three categories: (I) "Dentist & dental institution", (II) "Patient", and (III) "Treatment". These categories were developed by the reviewers. The category "Dentist & dental institution" comprises a total of *n* = 44 factors, divided into four subcategories: (I.1) "Access to care" (e.g., factor 'location', 'transportation'); (I.2) "Communication" (e.g., 'transparency', 'understandable information'); (I.3) "Qualification" (e.g., 'academic institution'); and (I.4) "Organization" (e.g., 'customer service', 'facilities'). The category "Patient" comprises three subcategories with a total of *n* = 37 factors representing patient characteristics: (II.1) "Medical characteristics" (e.g., 'emergency', 'prevention'); (II.2) "Social characteristics" (e.g., 'religion', 'social environment'); and (II.3) "Individual characteristics" (e.g., 'aesthetics', 'pain'). The category "Treatment", with *n* = 20 factors, is divided into two subcategories: (III.1) "Treatment characteristics" (e.g., 'complexity of treatment', 'tooth saving') and (III.2) "Costs" ('out-of-pocket payment', 'second opinion', 'installment'). Code frequencies showed that 'out-of-pocket payment' (*n* = 151) is also ranked first in this analysis, again followed by 'dental fear' (*n* = 73), 'aesthetics' (*n* = 64), and 'pain' (*n* = 61). Overall, *n* = 23 codes were used only once, e.g., 'quality assessment culture', 'social class', and 'willingness-to-travel' (WTT).

**Fig. 2:**
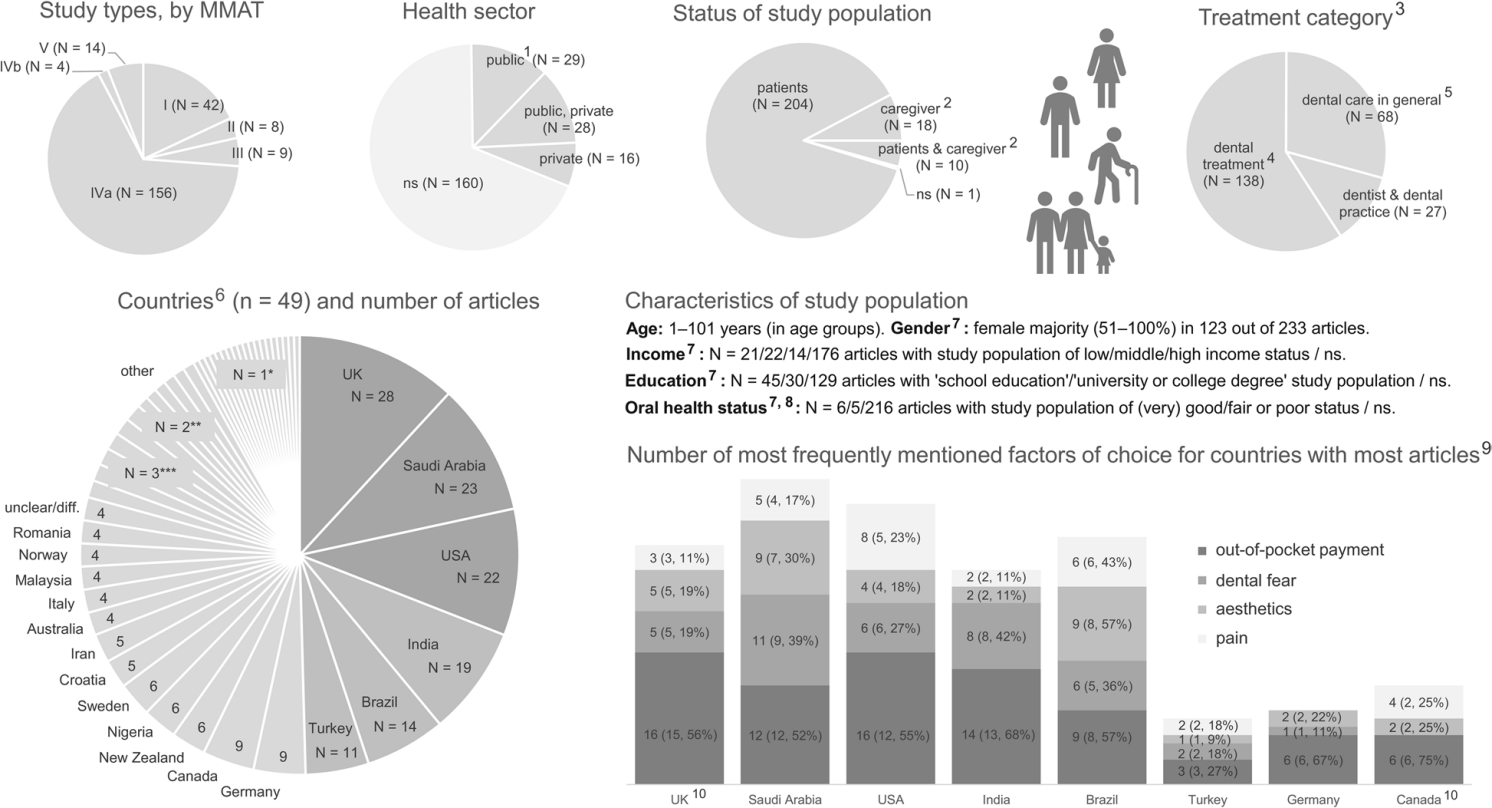
Factors of choice and descriptive results of the identified articles; diff. – different; MMAT – Mixed Methods Appraisal Tool (I – qualitative studies; II – quantitative randomized controlled trials (RCTs); III – quantitative non-randomized controlled trials (non-RCTs); IV – quantitative descriptive studies: a – cross-sectional, b – longitudinal; V – mixed-methods studies); ns – not stated or unclear; N – number of articles; 1 – including military hospitals; 2 – e.g., parents or relatives; 3 – categories into which the dental treatments analyzed in the articles were grouped (for further information and examples, see additional file A6.); 4 – e.g., implants, veneers; 5 – e.g., dental visits, toothache pain; 6 – articles covering multiple countries ('multi-country article') are counted once per country; 7 – majority or all of the study population; mixed-methods studies counted multiple times if applicable; 8 – self-perceived or dentist-diagnosed; 9 – bars represent the total frequency with which each factor was identified across articles for a given country (multiple mentions within an article were possible); values in brackets indicate the number of articles in which the factor was identified and, after the comma, the percentage relative to that country’s included articles; 10 – one multi-country article was excluded from this country-specific analysis because factor attribution was not feasible; denominators therefore are the UK: *N* = 27 articles, Canada: *N* = 8; * Bosnia and Herzegovina, Finland, France, Greece, Hong Kong, Kuwait, Lebanon, Mexico, Philippines, Poland, Portugal, Russia, South Korea, Sudan, Syria, Taiwan, Tanzania, Trinidad, Spain; ** Austria, Belgium, Bulgaria, Chile, Pakistan, Singapore; *** China, Ireland, Jordan, Netherlands, Switzerland, Thailand

In addition, in seven out of the eight countries with the most identified articles (i.e., the UK (*N* = 28), Saudi Arabia (*N* = 23), the USA (*N* = 22), India (*N* = 19), Brazil (*N* = 14), Turkey (*N* = 11), Germany (*N* = 9), and Canada (*N* = 9)), the factor 'out-of-pocket payment' was most prevalent. For example, these include Canada (*N* = 6, in 67% of the country’s articles [85, 93, 98–101]), India (*N* = 13, 68% [57, 61, 73, 102–111]), Germany (*N* = 6, 67% [77, 112–116]), the UK (*N* = 15, 54% [14, 70, 92, 96, 116–126]), and the USA (*N* = 12, 55% [46, 60, 65, 67, 97, 127–133]). Frequencies of the factors 'dental fear', 'aesthetics', and 'pain' vary among the countries. For example, 'dental fear' is frequently mentioned in articles from India (*N* = 8, 42% [57, 63, 66, 102, 103, 107, 108, 110]), Saudi Arabia (*N* = 9, 39% [16, 68, 134–140]), and Brazil (*N* = 5, 36% [141–145]), but it has less prominence in articles from Germany (*N* = 1, 11% [146]) and Canada (*N* = 0). 'Aesthetics' is mentioned most frequently in Brazilian articles (*N* = 8, 57% [62, 141–144, 147–149]), followed by articles from Saudi Arabia (*N* = 7, 30% [81, 86, 91, 135, 137, 150, 151]). In contrast, this factor is less often mentioned in articles from other countries, e.g., India (*N* = 2, 11% [73, 152]) and Turkey (*N* = 1, 9% [153]). 'Pain' is frequently mentioned as a factor of choice in studies conducted in Brazil (*N* = 6 articles, 43% [142–144, 149, 154, 155]), followed by Canada (*N* = 2 articles, 25% [101, 156]), and the USA (*N* = 5 articles, 23% [67, 76, 97, 128, 129]). However, this factor was not identified in the German context (*N* = 0). Fig. 2 presents a country-specific breakdown of the four most frequently mentioned factors of choice ('out-of-pocket payment', 'dental fear', 'aesthetics', and 'pain'). Bar heights indicate the total frequency with which each factor was identified across articles for a given country, while percentages denote the proportion relative to that country’s included articles (see figure legend for details). For this analysis, we only considered articles in which the identified factors could be clearly assigned to a single country; this was not possible for one multi-country article [53]. In addition, Fig. 2 provides an overview of the characteristics of the identified articles (e.g., study designs, health sectors in which the studies were conducted) and the number of articles per country.

**Fig. 3:**
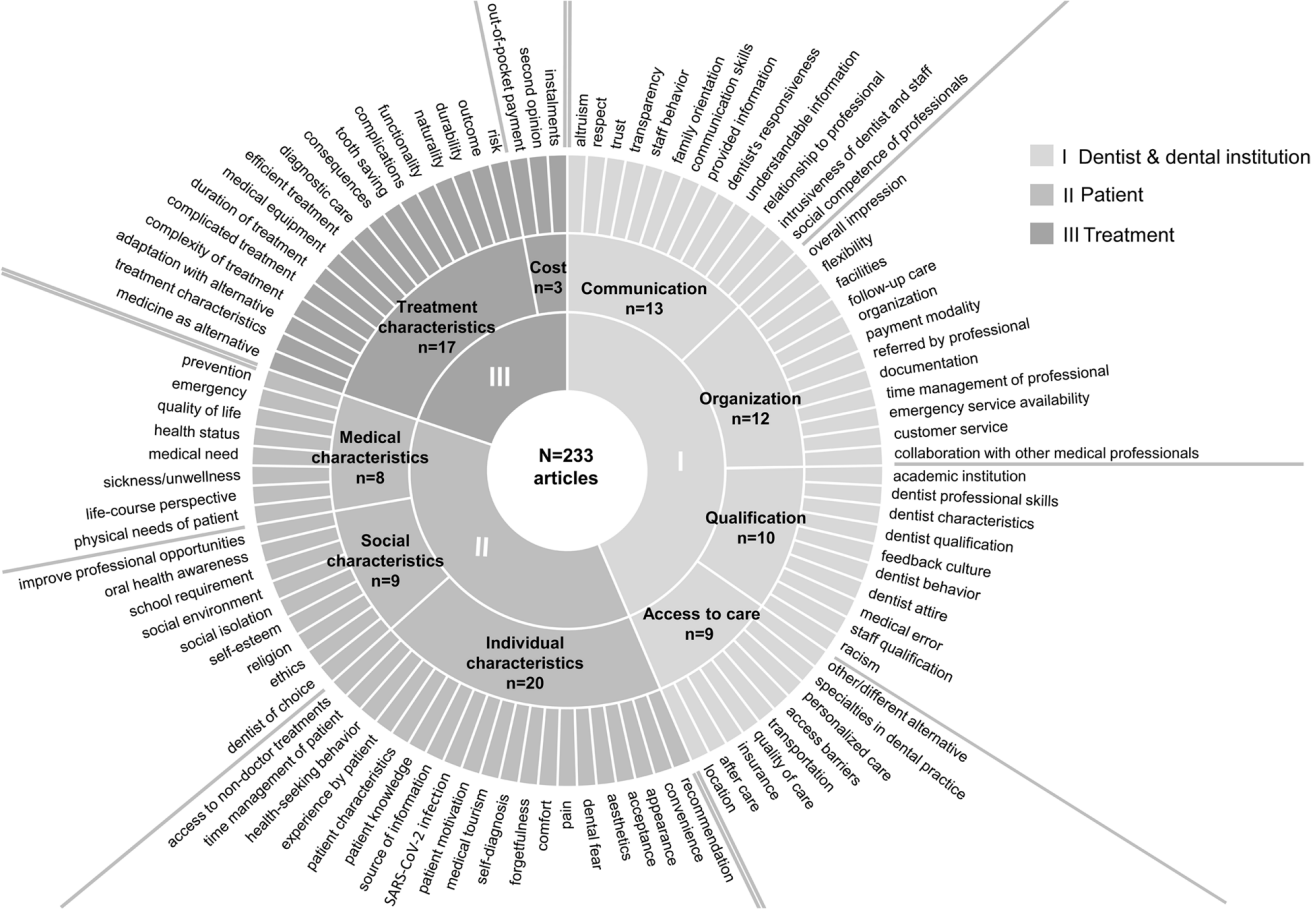
Factors of choice in dental care from patients’ perspective in (sub-)categories, *n* = 101

Fig. 3 shows the factors of choice, subdivided into categories and subcategories. The additional files contain the coding scheme and the codebook, including the framework for coding (A8. Coding scheme, codebook, and framework), and the definitions of the final codes (A9. Code definitions), representing the factors of choice.

#### Willingness-to-pay in dental care

As 'out-of-pocket payment' is the most frequently identified factor, we additionally provide an overview of the *N* = 37 articles analyzing willingness-to-pay (WTP) of patients (*N* = 3 RCTs, *N* = 6 non-RCTs, *N* = 26 descriptive studies (cross-sectional), and *N* = 2 mixed-methods studies). Most of the articles examined WTP for a particular dental treatment (*N* = 29, e.g., dental implant [150], filling replacement [157], fluoride varnish [124]). *N* = 6 articles examined WTP for a "dental service in general" (e.g., dental tourism [112], dental services [158]), and *N* = 2 articles for the category "dentist & dental office" (e.g., osteoporosis risk assessment in primary dental care [159]). Different methodological approaches were used to determine WTP, e.g., the bidding game and the payment card method, with open (e.g., [160]) and closed (e.g., [159]) response options. For example, Re et al. [161–163] reported that patients would be willing to pay an additional fee to receive a particular dental treatment. According to McKenna et al. [164], there is a strong WTP for basic and functional treatments. For more aesthetic treatments, WTP is even higher [96, 148]. Individual (sociodemographic) characteristics of patients (e.g., age [148], gender [150], income [150, 157, 165], education level and number of missing teeth [166]) influence WTP. Widström et al. [157] reported that individuals with higher incomes are willing to pay higher prices than those with lower incomes. However, some articles also reported that patients are not willing to pay any price for dental treatments, particularly when costs are high [167, 168].

#### Impact of COVID-19 behavior

The update search of the databases was conducted during the COVID-19 pandemic. As the pandemic was an extraordinary situation that affected patients’ utilization of medical services (as described in the *Background* section), a short paragraph is dedicated to this topic in the following.

Our analysis showed that COVID-19 influenced patients’ choice regarding dental treatments in the USA, which was reported in one of the articles identified: Papautsky et al. [169] used a convenience-based, non-representative sample of 2,570 US patients in their mixed-methods study to investigate reasons for non-utilization of (dental) medical treatments at the onset of the COVID-19 pandemic via an online survey. They found that fear of SARS-CoV-2 infection led patients to postpone and cancel dental treatments. In fact, dental care respondents were most likely to report delays (38.1%), compared to other medical areas (e.g., preventive care (29.2%) and diagnostic services (16.4%)). Age, gender, education, and self-reported concerns about overall health were significantly associated with the postponement of all (dental) medical services considered.

### Quality of studies

The ICR coefficient for the initial search was moderate but unacceptable (κ = 0.41) [41]. Therefore, a cross-check quality assessment was conducted on a randomly selected minimum sample of *N* = 47 articles from the literature hit pool of the update search (using RStudio (version 1.2.5)). This sample of articles was assessed independently by the two reviewers SF and JFH, using the MMAT. Subsequently, the results of the assessment, including questions S1 and S2, and the five questions regarding study design, were compared, and the ICR was calculated. In the second assessment round, a substantial value of κ = 0.697 was obtained. Following this, SF finalized the quality assessment after discussing and reaching consensus on the MMAT results for the minimum sample articles. The results of the ICR (and ICA), as well as the references of the articles included in the cross-check, can be found in the additional files (A10. Calculation of ICA and ICR; A7. Methodological characteristics of included articles (N = 233), and search details).

The quality of the studies, assessed by MMAT, varied. The quality scores ranged between 0 (*N* = 1) and 1.0 (*N* = 55) with most articles scoring 0.8 (*N* = 86) (0.2: *N* = 10, 0.4: *N* = 25, 0.6: *N* = 40). *N* = 11 articles failed question S1 and *N* = 5 articles failed question S2. These were excluded from the score assessment. In all study designs, articles could be identified with the highest quality score (score 1.0), which was given even most (modus) in qualitative studies and non-RCTs (e.g., [88], [141], [150], [163]). The lowest quality score of 0 was the assessment result of an RCT article [85]. A poor-quality score of 0.2 was found for articles among the quantitative descriptive studies (e.g., [60, 170, 171]) and for one article in the qualitative studies [97]. While MMAT scores were not used for exclusion or weighting, readers should interpret factors of choice primarily identified in studies with lower quality scores with increased caution regarding their methodological rigor and potential for bias, as discussed in the *Discussion* section. An overview of the MMAT assessment of all articles in tables is given in the additional files (A11.–A15. Quality assessment by MMAT: study design I–V). Furthermore, a detailed description of the MMAT assessment results can be found in another additional file (A16. MMAT assessment results description).

## Discussion

### Overview of key factors influencing dental treatment decisions

Our systematic review identified a wide range of factors that influence patients’ decision-making regarding dental treatments. These factors can be grouped into three overarching categories: (I) "Dentist & dental institution", (II) "Patient", and (III) "Treatment", as already identified in a previous study [34]. Among the most frequently reported factors across countries were 'out-of-pocket payment' (i.e., direct costs borne by patients), 'dental fear' (e.g., anxiety related to pain or complications), 'aesthetics' (patients’ desire for visually pleasing results), and 'pain' (experienced before, during, or after treatment). Individual factors in these categories often reflect predetermined, invariant characteristics of dental treatments (category "Treatment", e.g., factor 'durability') and the individual patient (category "Patient", e.g., factor 'health status'). Some factors, on the other hand, can be adapted and might therefore serve patients’ preferences (category "Dentist & dental institution", e.g., factor 'customer service'). However, while this study comprehensively mapped factors of choice, the overall certainty of the synthesized evidence for each factor, as per frameworks like GRADE, was not formally assessed, given the descriptive nature of the synthesis and the focus on factor identification rather than intervention effects.

### Out-of-pocket payments as a central barrier

Out-of-pocket payments have emerged as a key determinant in dental treatment decisions, as recently shown in willingness-to-pay studies from various countries, e.g., Japan [172], England [96], and Germany [173]. In particular, 'out-of-pocket payment' was most frequently mentioned as a factor of choice in the articles from the UK, Saudi Arabia, the USA, India, Brazil, Turkey, Germany, and Canada, the countries with the most articles identified (*N* ≥ 9). In Canada, for example, its relevance is underscored by the fact that patients have to privately pay for up to 94% of dental services [174]. This is similar in other countries, where patients often have to pay for dental treatments out of pocket because public health insurances only cover parts of dental treatment costs, e.g., there are co-payments for defined standard care for dentures in Germany [34]. Only certain dental treatments, e.g., preventive measures, are covered fully by statutory health insurance (SHI) in Germany [175] and, as another example, by the National Health Service (NHS) in the UK [176]. These examples highlight that insurance coverage varies significantly between countries, which helps to explain why out-of-pocket payments remain a central factor of choice in patients’ treatment decisions.

In the USA, dental treatments need to fulfill defined criteria (linked to medical condition) to be covered by the public health insurance programme "Medicare" [177]. These reasons could be decisive for the fact that (preventive) dental care is neglected by patients. Moreover, our results confirm that out-of-pocket payments lead patients to postpone or cancel dental appointments [77, 132]. Overall, this could have a negative impact on population oral health [27, 178], as seen in the USA, with a high proportion of untreated caries in various age groups [177, 179].

Implementing measures such as the "bonus booklet" in Germany, which reduces the financial burden for patients [34], may incentivize patients to access preventive dental care [180]. Health programmes such as those implemented in India may enable access to affordable and quality dental care and are thus a necessary step towards improving oral health [181]. Here, a previous state programme provided only limited coverage for dental treatments, and patients had to pay out of pocket for dental care [182]. In contrast, certain health centers and hospitals provide basic treatments (e.g., dental caries screening and treatment, prosthetic care), covered by "Ayushman Bharat – Pradhan Mantri Jan Arogya Yojana (AB-PMJAY)" [182, 183]. However, due to the limited availability of these facilities, patients are forced to visit general practitioners or private dental clinics, which, in turn, can result in high out-of-pocket payments for dental treatments [184].

In Brazil, dental care has been provided by the "Unified Health System – Sistema Único de Saúde (SUS)" since the national initiative "Smiling Brazil" in 2004 and is accessible to everyone free of charge [185, 186]. Public services include oral hygiene instructions, dental restorations, root canal treatments, tooth extractions, and even dental implants, with the latter being covered by only a few health care systems in the world [185]. However, the provision of these public services is concentrated in large urban regions in Brazil, with populations of good socioeconomic status [187]. In addition, insurance coverage of dental care is inconsistent across the overall population in Brazil [188, 189]. Patients opt for private dentists, where they have to pay for dental services themselves, because public services often mean long waiting times and varying levels of quality [185, 190]. This, and the fact that the identified Brazilian articles primarily focus on high-cost dental treatments (e.g., orthodontic treatment [143, 148, 149], prosthodontics [141, 142, 145]), could explain the high weight of the factor 'out-of-pocket payment' with respect to Brazil, according to our analysis.

In Saudi Arabia, all citizens and residents have free and full access to dental health services funded by initiatives such as the "National Transformation Program (NTP)" [191]. Nevertheless, patients opt for private services and accept additional payments because there is no guarantee of nationwide provision of dental services, and they prefer better quality of care, as private services are perceived to offer better treatment outcomes and a more personalized dentist-patient interaction compared to the public sector [192].

Turkey is an exception to the high weighting of out-of-pocket payments [153, 193]. Until the reform, dental treatments often had to be paid privately by patients. In 2008, the national health insurance scheme "Türkiye Cumhuriyeti Sosyal Güvenlik Kurumu (SGK)" was introduced, which covers dental services, especially certain treatments for children (e.g., orthodontic treatment, root canal treatment). In addition, people with low socioeconomic status who are not members of the SGK are entitled to dental care (except orthodontic treatments and dentures). However, due to the high patient demand, there are long waiting times for dental services, especially in rural areas [194]. Furthermore, there are no protective measures for high-risk and disadvantaged patient groups (e.g., pregnant women, disabled and elderly people) [195]. For these reasons, it is not possible to make a conclusive assessment of the importance of the factor 'out-of-pocket payment' in Turkey. Overall, despite various reforms, out-of-pocket payments remain a significant barrier for patients globally.

It is worth noting that in addition to analyzing WTP, similar approaches have also been used in some studies to determine patients’ "willingness" regarding utilization of dental services, e.g., willingness-to-accept (WTA) [85, 167] and WTT [158]. These studies are suitable for investigating reasons for choosing dental treatments in patients for whom out-of-pocket payments are not the decisive factor, perhaps pointing the way for future research.

### Interactions with other patient-specific factors: dental fear, aesthetics, and pain

The factor 'dental fear' is frequently reported in articles from India [57, 63, 66, 102, 103, 107, 108, 110], Saudi Arabia [16, 68, 134–140], and Brazil [141–145]. In these countries, access to (high-quality) dental care is limited [192], which might contribute to the development of advanced and painful dental conditions. In addition, there may be a lack of patient education on dental care and oral hygiene [182, 185]. This could cause fear in patients regarding pain, poor treatment results, and treatment complications.

In terms of the factor 'aesthetics', Brazil [62, 141–144, 147–149] and Saudi Arabia [81, 86, 91, 135, 137, 150, 151] stand out. Aesthetic aspects seem to be of great importance to the populations of these countries, a trend that is also observable in other medical disciplines such as plastic surgery [196–198]. However, these aesthetic preferences may be shaped by cultural norms, societal ideals of beauty, and the perceived social value of dental appearance [199–201]. There are societies and countries, such as Brazil and Saudi Arabia, where a bright, symmetrical smile is associated with higher social status, attractiveness, and professionalism, which can strongly influence treatment choices [202–204]. In contrast, in other regions, aesthetic considerations may be secondary to functional outcomes or cost, as identified in studies from the UK and India, for example [205, 206]. Understanding such regional differences is essential for tailoring dental health communication and care delivery. Without accounting for these contextual nuances, treatment plans and health interventions may fail to align with patients’ expectations and motivations, ultimately limiting their effectiveness.

Overall, 'pain' is identified as a factor of choice in articles from almost all countries considered (*n* = 34 countries, including articles focusing on *n* > 1 countries). Pain is a patient-specific factor, influenced by patients’ pain perception and the type of dental treatment [207, 208]. Therefore, future studies should address pain in a differentiated manner, particularly regarding its origin. Measures should be developed to reduce patients’ fear of pain, e.g., through (dental) health education, treatment tailored to patients, and enhanced shared decision-making [209, 210].

### Interrelated decision-making factors and their implications

The findings also suggest that individual factors rarely operate in isolation. Rather, they interact in complex ways to influence patients’ decisions. For example, out-of-pocket payments may exacerbate the impact of dental fear: patients already anxious about treatment may be even less willing to access care when confronted with financial burdens [211, 212]. Conversely, trust in the dentist and dental staff can mitigate the negative effects of both fear and cost concerns by enhancing patients’ confidence in the necessity and value of care [213]. These interdependencies highlight the need for multidimensional strategies that address both emotional and structural barriers to dental service utilization.

### COVID-19 and fear of infection

Only one article was found that directly addressed the COVID-19 pandemic. Papautsky et al. [169] identified patients’ fear of SARS-CoV-2 infection as a barrier to seeking dental treatments. The authors emphasized that findings from this pandemic should inform future health system planning and highlight the need for further research regarding the short- and long-term consequences of patients postponing or avoiding medical care. As our update search was conducted in a short time window during the pandemic, we found very few results on the topic. However, more recent publications, such as those by González-Olmo et al. [214] and Nikolić et al. [215], both published in 2022, confirm these findings and underline the importance of addressing this issue. Accordingly, greater attention should be paid to the factor fear of 'SARS-CoV-2 infection', which may reflect broader concerns about contracting respiratory diseases in (dental) medical settings.

### Addressing key barriers to dental care utilization

A thorough understanding of the reasons that prevent patients from seeking dental care, such as dental fear, fear of SARS-CoV-2 infection, concerns about aesthetics, and fear of pain, is essential for reducing barriers to access and utilization, and constitutes a prerequisite for improving oral health. The results of this study contribute significantly to improving this understanding and may help to identify where patients’ concerns could be addressed (e.g., by adapting regulatory measures in health care systems).

In particular, the factor 'out-of-pocket payment' as the most reported reason for patients’ choice regarding dental treatments, highlights the importance of strengthening oral health service delivery as part of an essential benefit package, as called for by the WHO [27]. Furthermore, dentists and other dental professionals could, for example, develop tailored communication and treatment strategies that consider the individual needs and concerns of patients. This patient-centered approach could not only promote the utilization of dental services, especially preventive measures, but might also significantly enhance patient satisfaction.

Creating an open, empathetic, and supportive environment might help dentists gain patients’ trust, which in turn could facilitate regular dental care and utilization of preventive measures. Indeed, one important factor identified is 'trust' in the dentist’s and dental staff’s decisions and actions, which can significantly influence patients’ willingness to undergo dental treatments [213, 216]. Trust is often built through continuity of care, respectful communication, and perceived competence, and can mitigate other barriers such as fear or uncertainty [213, 217].

### Cultural and social influences

Additional insights can be acquired by comprehending the connections between the identified factors of choice. Among other things, the cultural background of patients should be emphasized here, as previously discussed regarding aesthetic aspects. Moreover, it was found that cultural preferences, for example, denture marking [218] and the shade of artificial teeth [152], influence patients’ expectations and thus their satisfaction with dental care. Furthermore, factors of choice may vary depending on cultural backgrounds with respect to the head of the family, society, and the authority of the dentist [82, 219, 220]. Cultural background not only shapes social structures and decision hierarchies but also impacts perceptions of care quality, patient autonomy, and acceptance of health interventions [221]. Therefore, culturally sensitive approaches in dental care are essential to ensure acceptance and effectiveness of treatments across heterogeneous populations. Social influence also plays an important role, as recommendations from family members, friends, and peers were mentioned as guiding reasons in patients’ decisions [14, 128, 220]. These word-of-mouth endorsements can shape perceptions of quality and trustworthiness of dental providers [222].

### Implications for future research

Our findings highlight the need for a personalized approach to health care and emphasize the importance of developing strategies tailored to the specific needs of patients, including patient education. In addition, relationships between factors of choice and other variables (e.g., patient characteristics) should be investigated in follow-up studies. Future research should focus on longitudinal and comparative studies to deepen the understanding of long-term effects and cultural influences on patients’ decision-making in dental care. In addition, exploring the impact of new technologies and information sources on patients’ preferences is recommended to illuminate the role of technological advancements and social media in their decision-making process [223–225]. Finally, a deeper investigation into psychological and emotional factors is essential to identify barriers to health care and to develop targeted interventions to improve patients’ education and motivation. These topic-specific research directions highlight the complexity of patients’ decision-making in dental care. Beyond these thematic aspects, broader methodological and conceptual implications can be drawn from this systematic review. Future research could consider additional sources of information, including grey literature, where appropriate, while remaining mindful of its diverse methodological quality, thereby helping to mitigate the risk of publication bias. To better capture the variety of patient preferences and contextual factors, comparative cross-country studies using standardized survey instruments could be designed. Longitudinal qualitative studies may offer insights into how decision-making factors evolve over time. Moreover, mixed-methods approaches combining qualitative insights with discrete-choice experiments or other stated preference techniques could allow for a deeper understanding of trade-offs patients are willing to make. To address the heterogeneity among existing studies, future (systematic) reviews could employ a meta-ethnographic synthesis or conduct subgroup analyses to better account for variations by country, treatment type, or patient population.

### Methodological considerations, strengths, and limitations of the study

#### Reflection on quality assessment

To our knowledge, this is one of the first studies to apply the current version of the MMAT [38], a tool specifically developed for assessing the quality of studies in (systematic) reviews that include mixed-methods designs. However, we identified some limitations of the MMAT in practice. In particular, the wording of certain questions could be more precise and better tailored to different study designs. This may have introduced subjectivity in the interpretation and application of individual criteria. Moreover, a learning effect over the course of the assessment cannot be ruled out, potentially influencing reviewer behavior over time.

#### Strengths and limitations of the study

This mixed-methods study combines the methodological strengths of a systematic review with those of a qualitative analysis by ensuring a high level of evidence and minimizing bias through rigorously applied inclusion and exclusion criteria, and a systematic search strategy. The transparent presentation of the study’s methodology promotes traceability and reproducibility, while the integration of findings from various articles provides a comprehensive picture of the research field and a solid basis for clinical and policy decisions. In addition, the flexible and open approach of the qualitative component enables the discovery of new, context-specific aspects and unexpected topics beyond the scope of quantitative analysis.

Despite its strengths, this study also has several limitations that should be considered when interpreting the findings. The inherent heterogeneity across included studies in terms of design, research questions, and patient populations, coupled with potential publication bias, may have led to an overemphasis on frequently reported factors like 'out-of-pocket payment' and 'dental fear', thereby limiting the generalizability of our conclusions and direct comparability of findings across countries and contexts. As a result, while our synthesis highlights key patterns and recurrent themes, the relative importance of specific factors should be interpreted with caution and in consideration of context-specific characteristics. Only articles in English and German were considered due to resource constraints, which may have introduced language bias. It is also possible that not all relevant publications were identified in the literature search, for example, due to missing search terms. Other potential limitations include the unavailability of article full-texts (*N* = 34). In addition, the presented study characteristics and their categories (e.g., income) may not be representative. These classifications (e.g., "low" income) reflect country-specific definitions, which may limit the comparability and reliability of cross-country interpretations. Not all articles reported data comprehensively. Consequently, there are gaps in the extraction table for some articles. Furthermore, the change in reviewers for the quality assessment of the update search (from SF and CCS to SF and JFH), which was necessitated by capacity constraints, may have introduced subtle inconsistencies in judgment, despite rigorous ICR checks and consensus discussions. The results of this study may also be biased because we did not focus on a single specific dental treatment. In fact, the articles reported results for different dental treatments, which also occurred at different frequencies.

## Conclusions

Studies from 49 countries with various designs identified a range of factors (e.g., 'out-of-pocket payment', 'dental fear') that influence patients’ decision-making regarding dental treatments. These factors relate to the characteristics of the dental provider and setting, the treatment itself, and the individual patient. Given the relationship between oral health and systemic disease, improving population-level oral health should be considered a public health priority, also due to its potential to reduce long-term health care expenditures. A better understanding of patients’ motivation for seeking dental care can inform the development of targeted interventions, such as awareness campaigns and health literacy initiatives, that encourage proactive dental care behaviors and contribute to improved oral health outcomes. To achieve these outcomes, it is essential to implement tailored regulatory and informational strategies that reduce access barriers and support equitable utilization of dental care.

## Supplementary Information

Additional file 1: A1. Guideline on literature search, selection, and analysis. A2. Search strategy. A3. PRISMA checklist. A4. SWiM checklist. A5. Search strings for databases, including hits. A6. Characteristics, factors of choice, and references of included articles (N = 233), sorted by number of identified articles per country (descending) within study designs I–V. A7. Methodological characteristics of included articles (N = 233), and search details. A8. Coding scheme, codebook, and framework, including definitions of excluded and summarized codes. A9. Code definitions. A10. Calculation of ICA and ICR. A11. Quality assessment by MMAT: study design I. A12. Quality assessment by MMAT: study design II. A13. Quality assessment by MMAT: study design III. A14. Quality assessment by MMAT: study design IV. A15. Quality assessment by MMAT: study design V. A16. MMAT assessment results description.

## Data Availability

The datasets used and/or analyzed during the current study, that are not included in this published article and the additional files, are available from the corresponding author on reasonable request.

## Abbreviations

AB-PMJAY: Ayushman Bharat – Pradhan Mantri Jan Arogya Yojana
COVID-19: Coronavirus disease 2019
Fig.: Figure
GRADE: Grading of Recommendations Assessment, Development and Evaluation
HIV: Human immunodeficiency virus
HTA: Health technology assessment
ICA: Inter-coder agreement
ICR: Inter-coder reliability
MMAT: Mixed Methods Appraisal Tool
n: Number of factors / code frequencies / countries
N: Number of articles
NHS: National Health Service
NTP: National Transformation Program
PICO(ST): Patient / problem, intervention, comparison, outcome (, study design, time period)
PRISMA: Preferred Reporting Items for Systematic reviews and Meta-Analyses
RCT: Randomized controlled trial
SARS-CoV-2: Severe Acute Respiratory Syndrome Coronavirus 2
SGK: Türkiye Cumhuriyeti Sosyal Güvenlik Kurumu
SHI: Statutory health insurance
SUS: Sistema Único de Saúde
SWiM: Synthesis Without Meta-analysis
TiAb: Title/abstract
UK: United Kingdom
US(A): United States (of America)
VAS: Visual Analogue Scale
WHO: World Health Organization
WTA: Willingness-to-accept
WTP: Willingness-to-pay
WTT: Willingness-to-travel
