# Supplementary material for: Decision-making regarding dental treatments – What factors matter from patients’ perspective? A systematic review
Source: BMC Oral Health. 2025 Nov 25;26:289. doi: 10.1186/s12903-025-07032-9 (PMC12903421; doi:10.1186/s12903-025-07032-9)
Supplement: Supplementary file 1 — Additional file 1: A1. Guideline on literature search, selection, and analysis. A2. Search strategy. A3. PRISMA checklist. A4. SWiM checklist. A5. Search strings for databases, including hits. A6. Characteristics, factors of choice, and references of included articles (N = 233), sorted by number of identified articles per country (descending) within study designs I–V. A7. Methodological characteristics of included articles (N = 233), and search details. A8. Coding scheme, codebook, and framework, including definitions of excluded and summarized codes. A9. Code definitions. A10. Calculation of ICA and ICR. A11. Quality assessment by MMAT: study design I. A12. Quality assessment by MMAT: study design II. A13. Quality assessment by MMAT: study design III. A14. Quality assessment by MMAT: study design IV. A15. Quality assessment by MMAT: study design V. A16. MMAT assessment results description. [file 12903_2025_7032_MOESM1_ESM.zip › A12_Quality_assessment_by_MMAT_study_design_II.docx]

**A12.** Quality assessment by MMAT: study design II

| **Quality assessment by Mixed Methods Appraisal Tool (MMAT): study design II – Quantitative randomized controlled trials (RCTs)** | | | | | | | | | | |
| --- | --- | --- | --- | --- | --- | --- | --- | --- | --- | --- |
| Questions to answer:  **S1. Are there clear research questions?**  **S2. Do the collected data allow to address the research questions?**  **2.1. Is randomization appropriately performed?** *(1) description how randomization schedule was generated, e.g., predetermined plan/sequence; (2) appropriate performance of allocation concealement that protects assignment in researchers and participants, e.g., central randomization by third party/ sequentially numbered, opaque, sealed envelopes*  **2.2. Are the groups comparable at baseline?** *Large differences in (1) group size, (2) significant differences in characteristics, (3) key prognostic factors, (4) exessive similarity in charateristics,  (5) surprising absence of key characteristics*  **2.3. Are there complete outcome data?** *Complete outcome data between 80%-95%*  **2.4. Are outcome assessors blinded to the intervention provided?** *Assessors should be unaware of who is receiving which intervention*  **2.5 Did the participants adhere to the assigned intervention?** *Did many participants surprisingly not participate in follow-up?* | | | | | | | | | | |
| **No.** | **Reference^1^: author (year)** | **S1. clear research questions** | **S2. data addresses research questions** | **2.1. appropriate randomization** | **2.2. comparable groups at baseline** | **2.3. complete outcome data** | **2.4. blinded outcome assessors** | **2.5 adherence of participants** | **Number of points** | **Quality score (points)** |
| II.1 | Harris et al. (2020) | yes | yes | 1 | 1 | 1 | 1 | 0 | 4 | 0.8 (****) |
| II.2 | Bender et al. (2007) | yes | yes | 1 | 1 | 1 | 0 | 1 | 4 | 0.8 (****) |
| II.3 | Andrade et al. (2013) | yes | yes | 0 | 1 | 1 | 0 | 1 | 3 | 0.6 (***) |
| II.4 | Esfandiari et al. (2009) | yes | yes | 0 | 0 | 0 | 0 | 0 | 0 | 0.0 |
| II.5 | Heydecke et al. (2008) | yes | yes | 0 | 1 | 1 | 1 | 1 | 4 | 0.8 (****) |
| II.6 | Felice et al. (2009) | yes | yes | 1 | 1 | 1 | 1 | 1 | 5 | 1.0 (*****) |
| II.7 | McKenna et al. (2016) | yes | yes | 1 | 1 | 1 | 0 | 1 | 4 | 0.8 (****) |
| II.8 | Albonni et al. (2021) | yes | yes | 0 | 1 | 1 | 1 | 0 | 3 | 0.6 (***) |
| **Legend:** ^1^ order of references according to Table A6 | | | | | | | | | | |
