# Supplementary material for: Decision-making regarding dental treatments – What factors matter from patients’ perspective? A systematic review
Source: BMC Oral Health. 2025 Nov 25;26:289. doi: 10.1186/s12903-025-07032-9 (PMC12903421; doi:10.1186/s12903-025-07032-9)
Supplement: Supplementary file 1 — Additional file 1: A1. Guideline on literature search, selection, and analysis. A2. Search strategy. A3. PRISMA checklist. A4. SWiM checklist. A5. Search strings for databases, including hits. A6. Characteristics, factors of choice, and references of included articles (N = 233), sorted by number of identified articles per country (descending) within study designs I–V. A7. Methodological characteristics of included articles (N = 233), and search details. A8. Coding scheme, codebook, and framework, including definitions of excluded and summarized codes. A9. Code definitions. A10. Calculation of ICA and ICR. A11. Quality assessment by MMAT: study design I. A12. Quality assessment by MMAT: study design II. A13. Quality assessment by MMAT: study design III. A14. Quality assessment by MMAT: study design IV. A15. Quality assessment by MMAT: study design V. A16. MMAT assessment results description. [file 12903_2025_7032_MOESM1_ESM.zip › A13_Quality_assessment_by_MMAT_study_design_III.docx]

**A13.** Quality assessment by MMAT: study design III

| **Quality assessment by Mixed Methods Appraisal Tool (MMAT): study design III – Quantitative non-randomized controlled trials (non-RCTs)** | | | | | | | | | | |
| --- | --- | --- | --- | --- | --- | --- | --- | --- | --- | --- |
| Questions to answer:  **S1. Are there clear research questions?**  **S2. Do the collected data allow to address the research questions?**  **3.1. Are the participants representative of the target population?** *(1) clear description of target population and sample (inclusion and exclusion criteria), (2) reasons why individuals choose not to participate and (3) attempts to achieve sample*  **3.2. Are measurements appropriate regarding both the outcome and intervention (or exposure)?** *(1) variables are clearly defined and accurately measured, (2) measurements are justified and appropriate answering research question, (3) measurements reflect what supposed to measure and (4) validated and reliability tested or gold standard*  **3.3. Are there complete outcome data?** *(1) data from 80%-95% of participants in follow-up, (2) drop-out-rate 5%/20%/30% of more than 1 year*  **3.4. Are the confounders accounted for in the design and analysis?** *No confounders expected or appropriate methods to control confounders mentioned?*  **3.5. During the study period, is the intervention administered (or exposure occurred) as intended? *Intervention studies:*** *(1) participants treated like planned in intervention, (2) presence of contamination, (3) unplanned co-intervention in one group;* ***observational studies:*** *if changes in exposure status, check if (1) influence to outcome of interest or (2) unplanned co-exposures occured* | | | | | | | | | | |
| **No.** | **Reference^1^: author (year)** | **S1. clear research questions** | **S2. data addresses research questions** | **3.1. representation of target population** | **3.2. appropriate measurements (outcome, intervention / exposure)** | **3.3. complete outcome data** | **3.4. confounders accounted** | **3.5. intervention administered / exposure occurred as intended** | **Number of points** | **Quality score (points)** |
| III.1 | Al Garni et al. (2012) | yes | yes | 1 | 1 | 1 | 1 | 1 | 5 | 1.0 (*****) |
| III.2 | Atchison et al. (2007) | yes | yes | 1 | 1 | 0 | 1 | 1 | 4 | 0.8 (****) |
| III.3 | Eyuboglu et al. (2020) | yes | yes | 0 | 1 | 0 | 0 | 1 | 3 | 0.6 (***) |
| III.4 | Yuzbasioglu et al. (2014) | yes | yes | 0 | 1 | 1 | 1 | 1 | 4 | 0.8 (****) |
| III.5 | Koberlein et al. (2011) | yes | yes | 1 | 1 | 0 | 1 | 1 | 4 | 0.8 (****) |
| III.6 | Al-Dwairi et al. (2014) | yes | yes | 1 | 1 | 1 | 1 | 1 | 5 | 1.0 (*****) |
| III.7 | Al-Quran et al. (2011) | yes | yes | 1 | 1 | 1 | 1 | 1 | 5 | 1.0 (*****) |
| III.8 | Fragouli et al. (2016) | yes | yes | 0 | 1 | 1 | 1 | 1 | 4 | 0.8 (****) |
| III.9 | Re et al. (2018) | yes | yes | 1 | 1 | 1 | 1 | 1 | 5 | 1.0 (*****) |
| **Legend:** ^1^ order of references according to Table A6 | | | | | | | | | | |
