# Supplementary material for: Decision-making regarding dental treatments – What factors matter from patients’ perspective? A systematic review
Source: BMC Oral Health. 2025 Nov 25;26:289. doi: 10.1186/s12903-025-07032-9 (PMC12903421; doi:10.1186/s12903-025-07032-9)
Supplement: Supplementary file 1 — Additional file 1: A1. Guideline on literature search, selection, and analysis. A2. Search strategy. A3. PRISMA checklist. A4. SWiM checklist. A5. Search strings for databases, including hits. A6. Characteristics, factors of choice, and references of included articles (N = 233), sorted by number of identified articles per country (descending) within study designs I–V. A7. Methodological characteristics of included articles (N = 233), and search details. A8. Coding scheme, codebook, and framework, including definitions of excluded and summarized codes. A9. Code definitions. A10. Calculation of ICA and ICR. A11. Quality assessment by MMAT: study design I. A12. Quality assessment by MMAT: study design II. A13. Quality assessment by MMAT: study design III. A14. Quality assessment by MMAT: study design IV. A15. Quality assessment by MMAT: study design V. A16. MMAT assessment results description. [file 12903_2025_7032_MOESM1_ESM.zip › A14_Quality_assessment_by_MMAT_study_design_IV.docx]

**A14.** Quality assessment by MMAT: study design IV

| **Quality assessment by Mixed Methods Appraisal Tool (MMAT): study design IV – Quantitative descriptive studies** | | | | | | | | | | |
| --- | --- | --- | --- | --- | --- | --- | --- | --- | --- | --- |
| Questions to answer:  **S1. Are there clear research questions?**  **S2. Do the collected data allow to address the research questions?**  **4.1. Is the sampling strategy relevant to address the research question?** *Random selection?, if not: source of sample relevant to target population + justification of sample frame + adequate sampling procedure*  **4.2. Is the sample representative of the target population?** *Clear description of target population & sample (sizes, in/exclusion criteria), reasons for not participating, recruitment*  **4.3. Are the measurements appropriate?** *Variables clearly defined & accurately measured, measurements reflect what supposed to measure, test of validity & reliability, gold standard measure, questionnaire: pre-test*  **4.4. Is the risk of nonresponse bias low?** *Low response rate, reasons for nonresponse or statistical compensation for nonresponse (e.g., imputation) reported?*  **4.5. Is the statistical analysis appropriate to answer the research question?** *Statistical analysis clearly stated since appropriate for design & research question or limited interpretation because problems in data analysis?* | | | | | | | | | | |
| **No.** | **Reference^1^: author (year)** | **S1. Clear research questions** | **S2. Data addresses research questions** | **4.1. Relevant sampling strategy** | **4.2. Representation**  **of sample** | **4.3. Appropriate measurements** | **4.4. Low risk of nonresponse bias** | **4.5. Appropriate statistical analysis** | **Number of points** | **Quality score (points)** |
| *a. Cross-sectional studies* | | | | | | | | | | |
| IVa.1 | Austin et al. (2009) | yes | yes | 1 | 1 | 1 | 0 | 1 | 4 | 0.8 (****) |
| IVa.2 | Fenton et al. (2021) | yes | yes | 0 | 1 | 1 | 1 | 1 | 4 | 0.8 (****) |
| IVa.3 | Furnham et al. (2009) | yes | yes | 1 | 1 | 0 | 1 | 1 | 4 | 0.8 (****) |
| IVa.4 | Geoghegan et al. (2019) | no | 0 | 0 | 0 | 0 | 0 | 0 | 0 | none |
| IVa.5 | Goodwin et al. (2011) | yes | yes | 1 | 1 | 0 | 1 | 1 | 4 | 0.8 (****) |
| IVa.6 | Hill et al. (2013) | yes | yes | 1 | 0 | 1 | 1 | 1 | 4 | 0.8 (****) |
| IVa.7 | Marshman et al. (2012) | yes | yes | 1 | 0 | 0 | 1 | 1 | 3 | 0.6 (***) |
| IVa.8 | Swami et al. (2011) | yes | yes | 0 | 0 | 0 | 0 | 1 | 1 | 0.2 (*) |
| IVa.9 | Vernazza et al. (2015a) | yes | yes | 1 | 0 | 1 | 1 | 1 | 4 | 0.8 (****) |
| IVa.10 | Al Shaman et al. (2019) | yes | yes | 1 | 1 | 0 | 1 | 1 | 4 | 0.8 (****) |
| IVa.11 | Aldaij et al. (2018) | yes | yes | 0 | 1 | 1 | 0 | 0 | 2 | 0.4 (**) |
| IVa.12 | Al-Hussyeen et al. (2010) | yes | yes | 1 | 1 | 1 | 1 | 1 | 5 | 1.0 (*****) |
| IVa.13 | Ali et al. (2020) | yes | yes | 1 | 0 | 1 | 1 | 1 | 4 | 0.8 (****) |
| IVa.14 | Al-Johany et al. (2010) | yes | yes | 1 | 0 | 1 | 1 | 1 | 4 | 0.8 (****) |
| IVa.15 | Alsarheed et al. (2011) | yes | yes | 0 | 1 | 1 | 1 | 1 | 4 | 0.8 (****) |
| IVa.16 | Alshukairy et al. (2020) | yes | yes | 1 | 1 | 1 | 1 | 1 | 5 | 1.0 (*****) |
| IVa.17 | AlZarea et al. (2017) | yes | yes | 1 | 1 | 0 | 1 | 1 | 4 | 0.8 (****) |
| IVa.18 | Bagher et al. (2019) | yes | yes | 1 | 1 | 1 | 0 | 1 | 4 | 0.8 (****) |
| IVa.19 | Bahammam et al. (2019) | yes | yes | 1 | 1 | 0 | 1 | 0 | 3 | 0.6 (***) |
| IVa.20 | Fatani et al. (2016) | yes | yes | 0 | 0 | 0 | 1 | 1 | 2 | 0.4 (**) |
| IVa.21 | Fawaz (2015) | yes | yes | 1 | 1 | 0 | 1 | 1 | 4 | 0.8 (****) |
| IVa.22 | Gaffar et al. (2014) | yes | yes | 1 | 0 | 1 | 1 | 1 | 4 | 0.8 (****) |
| IVa.23 | Kakti et al. (2020) | yes | yes | 1 | 1 | 0 | 0 | 1 | 3 | 0.6 (***) |
| IVa.24 | Madarati et al. (2018) | yes | yes | 1 | 0 | 1 | 1 | 1 | 4 | 0.8 (****) |
| IVa.25 | Mubaraki et al. (2017) | yes | yes | 1 | 1 | 0 | 1 | 0 | 3 | 0.6 (***) |
| IVa.26 | Quadri et al. (2018) | yes | yes | 1 | 1 | 1 | 1 | 1 | 5 | 1.0 (*****) |
| IVa.27 | Sabbagh et al. (2020) | yes | yes | 1 | 1 | 1 | 0 | 1 | 4 | 0.8 (****) |
| IVa.28 | Shabbir et al. (2018) | yes | yes | 1 | 1 | 0 | 0 | 1 | 3 | 0.6 (***) |
| IVa.29 | Shahrani et al. (2015) | yes | yes | 1 | 0 | 0 | 0 | 1 | 2 | 0.4 (**) |
| IVa.30 | Taibah et al. (2018) | yes | yes | 1 | 0 | 1 | 1 | 1 | 4 | 0.8 (****) |
| IVa.31 | Chambers et al. (2019) | yes | yes | 0 | 0 | 1 | 0 | 0 | 1 | 0.2 (*) |
| IVa.32 | Cohen et al. (2008) | yes | yes | 1 | 1 | 0 | 1 | 1 | 4 | 0.8 (****) |
| IVa.33 | Crystal et al. (2017) | yes | yes | 0 | 1 | 1 | 0 | 1 | 3 | 0.6 (***) |
| IVa.34 | Flores et al. (2008) | yes | yes | 1 | 1 | 1 | 1 | 0 | 4 | 0.8 (****) |
| IVa.35 | Keerthana et al. (2020) | yes | yes | 0 | 1 | 0 | 1 | 1 | 3 | 0.6 (***) |
| IVa.36 | Kim et al. (2012) | yes | yes | 1 | 0 | 0 | 0 | 1 | 2 | 0.4 (**) |
| IVa.37 | Olson et al. (2020) | yes | yes | 1 | 1 | 0 | 1 | 1 | 4 | 0.8 (****) |
| IVa.38 | Samuels et al. (2015) | yes | yes | 1 | 1 | 1 | 0 | 1 | 4 | 0.8 (****) |
| IVa.39 | Vela et al. (2012) | yes | yes | 1 | 0 | 0 | 0 | 1 | 2 | 0.4 (**) |
| IVa.40 | Asokan et al. (2016) | yes | yes | 0 | 1 | 1 | 1 | 1 | 4 | 0.8 (****) |
| IVa.41 | Bhatia et al. (2018) | yes | yes | 1 | 1 | 1 | 1 | 1 | 5 | 1.0 (*****) |
| IVa.42 | Garcha et al. (2014) | yes | yes | 1 | 1 | 1 | 1 | 1 | 5 | 1.0 (*****) |
| IVa.43 | Jayakaran et al. (2017) | yes | yes | 0 | 0 | 0 | 0 | 1 | 1 | 0.2 (*) |
| IVa.44 | Kamavaram Ellore et al. (2015) | yes | yes | 0 | 0 | 0 | 0 | 1 | 1 | 0.2 (*) |
| IVa.45 | Kelly et al. (2014) | yes | no | 0 | 0 | 0 | 0 | 0 | 0 | none |
| IVa.46 | Mahajan et al. (2021) | yes | no | 0 | 0 | 0 | 0 | 0 | 0 | none |
| IVa.47 | Manickam et al. (2010) | yes | yes | 1 | 0 | 0 | 0 | 1 | 2 | 0.4 (**) |
| IVa.48 | Paul et al. (2019) | yes | yes | 1 | 1 | 1 | 1 | 0 | 4 | 0.8 (****) |
| IVa.49 | Poudyal et al. (2010) | yes | yes | 1 | 1 | 1 | 1 | 1 | 5 | 1.0 (*****) |
| IVa.50 | Pragati (2010) | yes | yes | 0 | 0 | 0 | 0 | 1 | 1 | 0.2 (*) |
| IVa.51 | Raj (2014) | yes | yes | 1 | 1 | 1 | 1 | 1 | 5 | 1.0 (*****) |
| IVa.52 | Ravikumar et al. (2016) | yes | yes | 0 | 1 | 1 | 0 | 0 | 2 | 0.4 (**) |
| IVa.53 | Saha et al. (2013) | yes | yes | 1 | 0 | 1 | 1 | 1 | 4 | 0.8 (****) |
| IVa.54 | Shah et al. (2014) | yes | yes | 1 | 1 | 0 | 0 | 1 | 3 | 0.6 (***) |
| IVa.55 | Shanmugam et al. (2020) | yes | yes | 0 | 1 | 1 | 1 | 1 | 4 | 0.8 (****) |
| IVa.56 | Shrirao et al. (2016) | yes | yes | 1 | 1 | 0 | 1 | 1 | 4 | 0.8 (****) |
| IVa.57 | Suprakash (2013) | yes | yes | 1 | 0 | 1 | 0 | 1 | 3 | 0.6 (***) |
| IVa.58 | Verma et al. (2012) | no | 0 | 0 | 0 | 0 | 0 | 0 | 0 | none |
| IVa.59 | Feldens et al. (2015) | yes | yes | 1 | 1 | 1 | 1 | 1 | 5 | 1.0 (*****) |
| IVa.60 | Feu et al. (2012) | yes | yes | 0 | 1 | 1 | 1 | 1 | 4 | 0.8 (****) |
| IVa.61 | Leles et al. (2009) | yes | yes | 1 | 0 | 1 | 1 | 1 | 4 | 0.8 (****) |
| IVa.62 | Leles et al. (2011) | yes | yes | 1 | 0 | 1 | 1 | 1 | 4 | 0.8 (****) |
| IVa.63 | Matsumoto et al. (2017) | yes | yes | 1 | 1 | 0 | 1 | 1 | 4 | 0.8 (****) |
| IVa.64 | Oliveira et al. (2013) | yes | yes | 0 | 0 | 0 | 1 | 1 | 2 | 0.4 (**) |
| IVa.65 | Souza et al. (2013) | yes | yes | 1 | 0 | 1 | 1 | 1 | 4 | 0.8 (****) |
| IVa.66 | Souza et al. (2016) | yes | yes | 1 | 0 | 0 | 1 | 1 | 3 | 0.6 (***) |
| IVa.67 | Vieira et al. (2015) | yes | yes | 0 | 1 | 0 | 0 | 1 | 2 | 0.4 (**) |
| IVa.68 | Gurler et al. (2018) | yes | yes | 1 | 1 | 0 | 1 | 0 | 3 | 0.6 (***) |
| IVa.69 | Kuscu et al. (2009) | yes | yes | 0 | 0 | 0 | 0 | 1 | 1 | 0.2 (*) |
| IVa.70 | Ozdogan et al. (2019) | yes | yes | 1 | 0 | 1 | 1 | 1 | 4 | 0.8 (****) |
| IVa.71 | Ozveren et al. (2021) | yes | yes | 0 | 1 | 0 | 0 | 1 | 2 | 0.4 (**) |
| IVa.72 | Patır Münevveroğlu et al. (2014) | yes | yes | 0 | 1 | 1 | 1 | 1 | 4 | 0.8 (****) |
| IVa.73 | Tengilimoglu et al. (2017) | yes | yes | 0 | 1 | 0 | 0 | 1 | 2 | 0.4 (**) |
| IVa.74 | Tuncer et al. (2015) | yes | yes | 1 | 0 | 0 | 0 | 1 | 2 | 0.4 (**) |
| IVa.75 | Uslu et al. (2007) | yes | yes | 0 | 0 | 0 | 0 | 1 | 1 | 0.2 (*) |
| IVa.76 | Yahyaoglu et al. (2018) | yes | yes | 0 | 1 | 1 | 1 | 1 | 4 | 0.8 (****) |
| IVa.77 | Azarpazhooh et al. (2015) | yes | yes | 1 | 0 | 1 | 0 | 1 | 3 | 0.6 (***) |
| IVa.78 | Balevi et al. (2007) | yes | yes | 1 | 1 | 1 | 1 | 1 | 5 | 1.0 (*****) |
| IVa.79 | Okuda et al. (2021) | yes | yes | 1 | 1 | 0 | 1 | 1 | 4 | 0.8 (****) |
| IVa.80 | Srivastava et al. (2014) | yes | yes | 0 | 0 | 1 | 1 | 1 | 3 | 0.6 (***) |
| IVa.81 | Srivastava et al. (2020) | yes | yes | 1 | 1 | 1 | 1 | 0 | 4 | 0.8 (****) |
| IVa.82 | Hirschfeld et al. (2019) | yes | yes | 1 | 1 | 1 | 1 | 1 | 5 | 1.0 (*****) |
| IVa.83 | Lamprecht et al. (2020) | yes | yes | 1 | 1 | 1 | 1 | 1 | 5 | 1.0 (*****) |
| IVa.84 | Rustemeyer et al. (2007) | yes | yes | 0 | 1 | 0 | 0 | 0 | 1 | 0.2 (*) |
| IVa.85 | Wang et al. (2021) | yes | yes | 1 | 1 | 1 | 1 | 1 | 5 | 1.0 (*****) |
| IVa.86 | Adedapo et al. (2011) | yes | yes | 1 | 1 | 0 | 1 | 1 | 4 | 0.8 (****) |
| IVa.87 | Ajayi et al. (2012) | yes | yes | 1 | 1 | 1 | 1 | 1 | 5 | 1.0 (*****) |
| IVa.88 | Ajayi et al. (2021) | yes | yes | 0 | 1 | 0 | 0 | 1 | 2 | 0.4 (**) |
| IVa.89 | Gbadebo et al. (2014) | yes | yes | 1 | 0 | 0 | 1 | 1 | 3 | 0.6 (***) |
| IVa.90 | Idowu et al. (2019) | yes | yes | 1 | 1 | 0 | 1 | 0 | 3 | 0.6 (***) |
| IVa.91 | Lawal et al. (2019) | yes | yes | 1 | 1 | 1 | 1 | 1 | 5 | 1.0 (*****) |
| IVa.92 | Christell et al. (2019) | yes | yes | 0 | 0 | 1 | 1 | 1 | 3 | 0.6 (***) |
| IVa.93 | Balenovic et al. (2019) | yes | yes | 0 | 0 | 0 | 1 | 1 | 2 | 0.4 (**) |
| IVa.94 | Sever et al. (2019) | no | 0 | 0 | 0 | 0 | 0 | 0 | 0 | none |
| IVa.95 | Sever et al. (2020) | yes | yes | 0 | 0 | 1 | 1 | 1 | 3 | 0.6 (***) |
| IVa.96 | Spalj et al. (2014) | yes | yes | 1 | 1 | 1 | 1 | 1 | 5 | 1.0 (*****) |
| IVa.97 | Bahadori et al. (2013) | yes | yes | 1 | 1 | 1 | 1 | 1 | 5 | 1.0 (*****) |
| IVa.98 | Moshkelgosha et al. (2013) | yes | yes | 1 | 1 | 1 | 1 | 1 | 5 | 1.0 (*****) |
| IVa.99 | Moshkelgosha et al. (2014) | yes | yes | 1 | 1 | 1 | 0 | 1 | 4 | 0.8 (****) |
| IVa.100 | Moshkelgosha et al. (2015) | yes | yes | 1 | 1 | 1 | 1 | 1 | 5 | 1.0 (*****) |
| IVa.101 | Saadatfar et al. (2021) | yes | yes | 1 | 1 | 1 | 1 | 1 | 5 | 1.0 (*****) |
| IVa.102 | Armfield et al. (2013) | yes | yes | 1 | 1 | 0 | 0 | 1 | 3 | 0.6 (***) |
| IVa.103 | Beresford et al. (2018) | yes | yes | 0 | 1 | 1 | 0 | 1 | 3 | 0.6 (***) |
| IVa.104 | Luzzi et al. (2008) | yes | yes | 1 | 1 | 0 | 1 | 1 | 4 | 0.8 (****) |
| IVa.105 | Angelis et al. (2020) | yes | yes | 0 | 1 | 1 | 1 | 1 | 4 | 0.8 (****) |
| IVa.106 | Augusti et al. (2014) | yes | yes | 1 | 0 | 0 | 1 | 1 | 3 | 0.6 (***) |
| IVa.107 | Re et al. (2017) | yes | yes | 1 | 1 | 0 | 1 | 1 | 4 | 0.8 (****) |
| IVa.108 | Jaafar et al. (2018) | yes | yes | 0 | 0 | 1 | 0 | 1 | 2 | 0.4 (**) |
| IVa.109 | Kohli et al. (2014) | yes | yes | 1 | 1 | 0 | 0 | 1 | 3 | 0.6 (***) |
| IVa.110 | Tin-Oo et al. (2011) | yes | yes | 1 | 1 | 1 | 1 | 1 | 5 | 1.0 (*****) |
| IVa.111 | Hansen Edwards (2013) | yes | yes | 0 | 1 | 1 | 1 | 0 | 3 | 0.6 (***) |
| IVa.112 | Nermo et al. (2019) | yes | yes | 1 | 1 | 1 | 1 | 1 | 5 | 1.0 (*****) |
| IVa.113 | Trovik et al. (2012) | yes | yes | 1 | 1 | 1 | 1 | 1 | 5 | 1.0 (*****) |
| IVa.114 | Vika et al. (2008) | yes | yes | 1 | 1 | 0 | 1 | 1 | 4 | 0.8 (****) |
| IVa.115 | Dudea et al. (2012) | yes | yes | 1 | 1 | 1 | 0 | 1 | 4 | 0.8 (****) |
| IVa.116 | Tâncu et al. (2019) | yes | yes | 0 | 0 | 0 | 1 | 1 | 2 | 0.4 (**) |
| IVa.117 | Tudorici et al. (2017) | yes | yes | 1 | 1 | 0 | 0 | 1 | 3 | 0.6 (***) |
| IVa.118 | Ungureanu et al. (2015) | yes | yes | 1 | 1 | 0 | 0 | 1 | 3 | 0.6 (***) |
| IVa.119 | Gao et al. (2020) | yes | yes | 1 | 1 | 0 | 1 | 1 | 4 | 0.8 (****) |
| IVa.120 | Leung et al. (2010) | yes | yes | 0 | 1 | 1 | 1 | 1 | 4 | 0.8 (****) |
| IVa.121 | Zhu et al. (2019) | no | 0 | 0 | 0 | 0 | 0 | 0 | 0 | none |
| IVa.122 | Shanahan et al. (2017) | yes | yes | 1 | 1 | 1 | 1 | 1 | 5 | 1.0 (*****) |
| IVa.123 | Al-Batayneh et al. (2019) | yes | yes | 0 | 1 | 1 | 1 | 1 | 4 | 0.8 (****) |
| IVa.124 | Vermaire et al. (2012) | yes | yes | 0 | 1 | 1 | 1 | 1 | 4 | 0.8 (****) |
| IVa.125 | Chebib et al. (2020) | yes | yes | 0 | 1 | 1 | 1 | 1 | 4 | 0.8 (****) |
| IVa.126 | Meier et al. (2021) | yes | yes | 0 | 1 | 0 | 1 | 1 | 3 | 0.6 (***) |
| IVa.127 | Sendi et al. (2017) | yes | no | 0 | 0 | 0 | 0 | 0 | 0 | none |
| IVa.128 | Tianviwat et al. (2008) | yes | yes | 1 | 0 | 1 | 1 | 1 | 4 | 0.8 (****) |
| IVa.129 | Tianviwat et al. (2009) | yes | yes | 1 | 1 | 1 | 1 | 1 | 5 | 1.0 (*****) |
| IVa.130 | Hof et al. (2014) | yes | yes | 1 | 0 | 0 | 0 | 1 | 2 | 0.4 (**) |
| IVa.131 | Pommer et al. (2011) | yes | yes | 1 | 1 | 1 | 1 | 1 | 5 | 1.0 (*****) |
| IVa.132 | van den Branden et al. (2013) | yes | yes | 1 | 1 | 0 | 1 | 1 | 4 | 0.8 (****) |
| IVa.133 | Atanasov et al. (2016) | yes | yes | 1 | 0 | 0 | 1 | 1 | 3 | 0.6 (***) |
| IVa.134 | Lalabonova et al. (2015) | yes | yes | 1 | 0 | 0 | 1 | 1 | 3 | 0.6 (***) |
| IVa.135 | Fernandez et al. (2015) | yes | yes | 1 | 0 | 1 | 1 | 1 | 4 | 0.8 (****) |
| IVa.136 | Rojas-Torres et al. (2019) | no | 0 | 0 | 0 | 0 | 0 | 0 | 0 | none |
| IVa.137 | Amjad (2014) | yes | yes | 1 | 1 | 0 | 1 | 1 | 4 | 0.8 (****) |
| IVa.138 | Saleem et al. (2018) | yes | yes | 0 | 0 | 0 | 1 | 1 | 2 | 0.4 (**) |
| IVa.139 | Nair et al. (2016) | yes | yes | 0 | 0 | 1 | 1 | 1 | 3 | 0.6 (***) |
| IVa.140 | Bajrić et al. (2015) | no | 0 | 0 | 0 | 0 | 0 | 0 | 0 | none |
| IVa.141 | Widström et al. (2012) | yes | yes | 1 | 0 | 0 | 0 | 1 | 2 | 0.4 (**) |
| IVa.142 | Chau et al. (2014) | yes | yes | 1 | 1 | 1 | 0 | 1 | 4 | 0.8 (****) |
| IVa.143 | Abdulwahab et al. (2010) | yes | yes | 1 | 1 | 0 | 1 | 1 | 4 | 0.8 (****) |
| IVa.144 | Malak et al. (2021) | yes | yes | 1 | 1 | 1 | 0 | 1 | 4 | 0.8 (****) |
| IVa.145 | Dalanon et al. (2018) | yes | yes | 1 | 0 | 0 | 0 | 0 | 1 | 0.2 (*) |
| IVa.146 | Wedrychowska-Szulc et al. (2010) | yes | yes | 0 | 1 | 0 | 0 | 1 | 2 | 0.4 (**) |
| IVa.147 | Tachalov et al. (2021) | yes | yes | 0 | 1 | 1 | 1 | 1 | 4 | 0.8 (****) |
| IVa.148 | Park et al. (2021) | no | 0 | 0 | 0 | 0 | 0 | 0 | 0 | none |
| IVa.149 | Awooda et al. (2014) | yes | yes | 1 | 0 | 1 | 1 | 1 | 4 | 0.8 (****) |
| IVa.150 | Nyamuryekunge et al. (2018) | yes | yes | 1 | 1 | 1 | 1 | 1 | 5 | 1.0 (*****) |
| IVa.151 | Bucchi et al. (2019) | yes | yes | 0 | 1 | 1 | 1 | 1 | 4 | 0.8 (****) |
| IVa.152 | Laothong et al. (2017) | yes | yes | 1 | 1 | 0 | 1 | 1 | 4 | 0.8 (****) |
| IVa.153 | Vernazza et al. (2015b) | yes | yes | 1 | 1 | 1 | 0 | 1 | 4 | 0.8 (****) |
| IVa.154 | Walshaw et al. (2019) | yes | yes | 1 | 1 | 1 | 1 | 1 | 5 | 1.0 (*****) |
| IVa.155 | Nalbandian et al. (2009) | yes | yes | 1 | 0 | 0 | 0 | 1 | 2 | 0.4 (**) |
| IVa.156 | Re et al. (2016) | yes | yes | 1 | 1 | 1 | 1 | 1 | 5 | 1.0 (*****) |
| *b. Longitudinal studies* | | | | | | | | | | |
| IVb.1 | Tilashalski et al. (2007) | yes | yes | 1 | 1 | 0 | 0 | 1 | 3 | 0.6 (***) |
| IVb.2 | Wall et al. (2015) | yes | yes | 1 | 0 | 1 | 0 | 0 | 2 | 0.4 (**) |
| IVb.3 | Aarabi et al. (2019) | yes | no | 0 | 0 | 0 | 0 | 0 | 0 | none |
| IVb.4 | Narby et al. (2008) | yes | yes | 1 | 1 | 0 | 1 | 1 | 4 | 0.8 (****) |
| **Legend:** ^1^ order of references according to Table A6 | | | | | | | | | | |
