# Supplementary material for: Decision-making regarding dental treatments – What factors matter from patients’ perspective? A systematic review
Source: BMC Oral Health. 2025 Nov 25;26:289. doi: 10.1186/s12903-025-07032-9 (PMC12903421; doi:10.1186/s12903-025-07032-9)
Supplement: Supplementary file 1 — Additional file 1: A1. Guideline on literature search, selection, and analysis. A2. Search strategy. A3. PRISMA checklist. A4. SWiM checklist. A5. Search strings for databases, including hits. A6. Characteristics, factors of choice, and references of included articles (N = 233), sorted by number of identified articles per country (descending) within study designs I–V. A7. Methodological characteristics of included articles (N = 233), and search details. A8. Coding scheme, codebook, and framework, including definitions of excluded and summarized codes. A9. Code definitions. A10. Calculation of ICA and ICR. A11. Quality assessment by MMAT: study design I. A12. Quality assessment by MMAT: study design II. A13. Quality assessment by MMAT: study design III. A14. Quality assessment by MMAT: study design IV. A15. Quality assessment by MMAT: study design V. A16. MMAT assessment results description. [file 12903_2025_7032_MOESM1_ESM.zip › A15_Quality_assessment_by_MMAT_study_design_V.docx]

**A15.** Quality assessment by MMAT: study design V

| **Quality assessment by Mixed Methods Appraisal Tool (MMAT): study design V – Mixed-methods studies** | | | | | | | | | | |
| --- | --- | --- | --- | --- | --- | --- | --- | --- | --- | --- |
| Questions to answer:  **S1. Are there clear research questions?**  **S2. Do the collected data allow to address the research questions?**  **5.1. Is there an adequate rationale for using a mixed methods design to address the research question?** *Reasons for MM cleary explained, e.g., build upon qual. findings with quant. results etc., complete understanding of a phenomenon or develop and test instruments*  **5.2. Are the different components of the study effectively integrated to answer the research question?** *Information how qual. and quant. phases/results/data were integrated, e.g., brought together to form a picture; when integration occured, e.g., during data collection or interpretation*  **5.3. Are the outputs of the integration of qualitative and quantitative components adequately interpreted?** *Meta-inference that occurs in interpretation after integration of quant. and qual. components and shows added value of a MM (rather than having 2 separate studies)*  **5.4. Are divergences and inconsistencies between quantitative and qualitative results adequately addressed?** *"Yes" no divergencies (= conflicts) when integrating findings from quant. and qual. results are reported (even if explained)* | | | | | | | | | | |
| **No.** | **Reference^1^: author (year)** | **S1. Clear research questions** | **S2. Data addresses research questions** | **5.1. Adequate rationale for mixed methods design** | **5.2. Effective integration of components** | **5.3. Adequate interpretation of outputs (qualitative, quantitative components)** | **5.4. Divergences / inconsistencies adequately addressed** | **5.5. Quality criteria correspond to methodological tradition** | **Number of points** | **Quality score (points)** |
| V.1 | Clarkson et al. (2020) | yes | yes | 0 | 0 | 1 | 1 | 1 | 3 | 0.6 (***) |
| V.2 | Harris et al. (2020) | yes | yes | 1 | 0 | 1 | 0 | 1 | 3 | 0.6 (***) |
| V.3 | Nayee et al. (2015) | yes | yes | 1 | 1 | 0 | 0 | 0 | 2 | 0.4 (**) |
| V.4 | Paisi et al. (2020) | yes | yes | 1 | 0 | 1 | 1 | 0 | 3 | 0.6 (***) |
| V.5 | El-Din (2008) | yes | yes | 1 | 1 | 1 | 1 | 1 | 5 | 1.0 (*****) |
| V.6 | Milner et al. (2019) | yes | yes | 1 | 1 | 0 | 1 | 1 | 4 | 0.8 (****) |
| V.7 | Papautsky et al. (2021) | yes | yes | 1 | 1 | 1 | 1 | 0 | 4 | 0.8 (****) |
| V.8 | Da Kfouri et al. (2019) | yes | yes | 0 | 0 | 1 | 1 | 0 | 2 | 0.4 (**) |
| V.9 | Maciel et al. (2017) | yes | yes | 1 | 1 | 1 | 1 | 0 | 4 | 0.8 (****) |
| V.10 | Azarpazhooh et al. (2016) | yes | yes | 1 | 1 | 1 | 0 | 0 | 3 | 0.6 (***) |
| V.11 | Schwendicke et al. (2016) | yes | yes | 1 | 1 | 1 | 1 | 1 | 5 | 1.0 (*****) |
| V.12 | Sever et al. (2018) | yes | yes | 1 | 1 | 1 | 1 | 1 | 5 | 1.0 (*****) |
| V.13 | Sonneveld et al. (2013) | yes | yes | 1 | 1 | 1 | 1 | 1 | 5 | 1.0 (*****) |
| V.14 | Jaapar et al. (2017) | yes | yes | 1 | 1 | 1 | 1 | 1 | 5 | 1.0 (*****) |
| **Legend:** MM – mixed-methods study, qual. – qualitative, quant. – quantitative, ^1^ order of references according to Table A6 | | | | | | | | | | |
