# Supplementary material for: Decision-making regarding dental treatments – What factors matter from patients’ perspective? A systematic review
Source: BMC Oral Health. 2025 Nov 25;26:289. doi: 10.1186/s12903-025-07032-9 (PMC12903421; doi:10.1186/s12903-025-07032-9)
Supplement: Supplementary file 1 — Additional file 1: A1. Guideline on literature search, selection, and analysis. A2. Search strategy. A3. PRISMA checklist. A4. SWiM checklist. A5. Search strings for databases, including hits. A6. Characteristics, factors of choice, and references of included articles (N = 233), sorted by number of identified articles per country (descending) within study designs I–V. A7. Methodological characteristics of included articles (N = 233), and search details. A8. Coding scheme, codebook, and framework, including definitions of excluded and summarized codes. A9. Code definitions. A10. Calculation of ICA and ICR. A11. Quality assessment by MMAT: study design I. A12. Quality assessment by MMAT: study design II. A13. Quality assessment by MMAT: study design III. A14. Quality assessment by MMAT: study design IV. A15. Quality assessment by MMAT: study design V. A16. MMAT assessment results description. [file 12903_2025_7032_MOESM1_ESM.zip › A16_MMAT_assessment_results_description.docx]

**A16.** MMAT assessment results description

The articles of the different study designs were scored using the Mixed Methods Appraisal Tool (MMAT) [1] as follows:

For qualitative studies, for example, no points were given for question 1.5 if the research question was not clearly answered in the articles’ results (e.g., [2]), or if no direct relationship between generated citations and derived results was apparent (e.g., [3]). For randomized controlled trials (RCTs), zero points were given if randomized group assignment was not performed appropriately (question 2.1), such as in Albonni et al. (2021) [4], where only flipping a coin was used during randomization. In addition, no points were given if the studies’ intervention and comparison groups were not comparable at baseline (question 2.2), for example in terms of group size (e.g., [5]). An example of a study receiving zero points for a non-RCT is found in Eyuboglu et al. (2020) [6], where the authors neither stated where the study participants were recruited (question 3.1), nor provided information on follow-up (question 3.3) or confounders (question 3.4). A common reason for zero points in quantitative descriptive studies was the lack of a pre-test when using non-validated questionnaires or guides (question 4.3) (e.g., [7, 8]). Other examples of articles that received zero points for specific questions are Aldaij et al. (2018) [9] and Wall et al. (2015) [10]. In both articles, it is not reported how many participants were considered in the analysis. As another example, Dalanon et al. (2018) [11] do not provide any information on the statistical significance of the results (question 4.5). Reasons for awarding zero points to articles on mixed-methods studies included the fact that it remained unclear how the different study designs fit together or were built on each other (question 5.1) (e.g., [12]). In addition, no points were given if information on pre-tests was missing (question 5.5), as seen in some articles (e.g., [13, 14]). An overview of the MMAT assessment of all articles is given in the additional files (**A11.**-**A15.** Quality assessment by MMAT: study design I-V).

**References**

1. Pluye P, Robert E, Cargo M, Bartlett G, O’Cathain A, Griffiths F et al. Proposal: a mixed methods appraisal tool for systematic mixed studies reviews. 2011. http://mixedmethodsappraisaltoolpublic.pbworks.com/w/page/24607821/FrontPage. Accessed 12 Sep 2023.

2. Johannsen A, Westergren A, Johannsen G. Dental implants from the patients perspective: transition from tooth loss, through amputation to implants - negative and positive trajectories. J Clin Periodontol. 2012;39:681–7. doi:10.1111/j.1600-051X.2012.01893.x.

3. Serban S, Dietrich T, Lopez-Oliva I, Pablo P de, Raza K, Filer A, et al. Attitudes towards oral health in patients with rheumatoid arthritis: a qualitative study nested within a randomized controlled trial. JDR Clinical & Translational Research. 2019;4:360–70. doi:10.1177/2380084419833694.

4. Albonni H, Alseirafi W, Tekleh H, Abo Orabi F, Alhaj M, Almasri D, et al. Clinical outcomes of using Erythritol powder by means of air polishing with ultrasonic debridement in the treatment of initial periodontal pockets in hand of dental students: a split-mouth, randomized, comparative, controlled study. Part I. Int J Dent Hyg 2021. doi:10.1111/idh.12519.

5. Esfandiari S, Lund JP, Penrod JR, Savard A, Mark Thomason J, Feine JS. Implant overdentures for edentulous elders: study of patient preference. Gerodontology. 2009;26:3–10. doi:10.1111/j.1741-2358.2008.00237.x.

6. Eyuboglu TF, Gonenc FI. The effect of pain intensity levels and clinical symptoms on the treatment preferences of patients with endodontically involved teeth: a preliminary cross-sectional study. Eur Oral Res. 2020;54:142–7. doi:10.26650/eor.20200043.

7. Armfield JM. What goes around comes around: revisiting the hypothesized vicious cycle of dental fear and avoidance. Community Dent Oral Epidemiol. 2013;41:279–87. doi:10.1111/cdoe.12005.

8. Gurler G, Delilbasi C, Kacar I. Patients' perceptions and preferences of oral and maxillofacial surgeons in a university dental hospital. Eur Oral Res. 2018;52:137–42. doi:10.26650/eor.2018.483.

9. Aldaij M, Alshehri T, Alzeer A, Alfayez A, Aldrees F, Almuhaya S, et al. Patient patisfaction with dental appearance and treatment desire to improve esthetics. Journal of Oral Health and Community Dentistry. 2018;12:90–5. doi:10.5005/jp-journals-10062-0033.

10. Wall T, Nasseh K, Vujicic M. Most important barriers to dental care are financial, not supply related. 2014. https://silo.tips/download/research-brief-most-important-barriers-to-dental-care-are-financial-not-supply-r. Accessed 12 Sep 2023.

11. Dalanon J, Diano LM, Esguerra R, Belarmino MP, Docor MR, Rodis OM, Locsin R, Matsuka Y. The Cebuano mothers’ willingness to pay for dental healthcare. The Journal of the PDA. 2018;65:33–7.

12. Clarkson JE, Pitts NB, Goulao B, Boyers D, Ramsay CR, Floate R, et al. Risk-based, 6-monthly and 24-monthly dental check-ups for adults: the INTERVAL three-arm RCT: National Institute for Health Research. Health technology assessment 2020. doi:10.3310/hta24600.

13. Paisi M, Baines R, Worle C, Withers L, Witton R. Evaluation of a community dental clinic providing care to people experiencing homelessness: a mixed methods approach. Health Expectations. 2020;23:1289–99. doi:10.1111/hex.13111.

14. Papautsky EL, Rice DR, Ghoneima H, McKowen ALW, Anderson N, Wootton AR, Veldhuis C. Characterizing health care delays and interruptions in the United States during the COVID-19 pandemic: internet-based, cross-sectional survey study. J Med Internet Res. 2021;23:e25446. doi:10.2196/25446.
