# Supplementary material for: Decision-making regarding dental treatments – What factors matter from patients’ perspective? A systematic review
Source: BMC Oral Health. 2025 Nov 25;26:289. doi: 10.1186/s12903-025-07032-9 (PMC12903421; doi:10.1186/s12903-025-07032-9)
Supplement: Supplementary file 1 — Additional file 1: A1. Guideline on literature search, selection, and analysis. A2. Search strategy. A3. PRISMA checklist. A4. SWiM checklist. A5. Search strings for databases, including hits. A6. Characteristics, factors of choice, and references of included articles (N = 233), sorted by number of identified articles per country (descending) within study designs I–V. A7. Methodological characteristics of included articles (N = 233), and search details. A8. Coding scheme, codebook, and framework, including definitions of excluded and summarized codes. A9. Code definitions. A10. Calculation of ICA and ICR. A11. Quality assessment by MMAT: study design I. A12. Quality assessment by MMAT: study design II. A13. Quality assessment by MMAT: study design III. A14. Quality assessment by MMAT: study design IV. A15. Quality assessment by MMAT: study design V. A16. MMAT assessment results description. [file 12903_2025_7032_MOESM1_ESM.zip › A1_Guideline_on_search_selection_and_analysis.docx]

**A1.** Guideline on literature search, selection, and analysis

in accordance with Perleth et al. (2014) [1]

**[1] Work steps of systematic literature search**

1. Transformation of research question into an answerable question using the PICO scheme [1]
2. Definition of research concept
3. Identification of search terms and synonyms
4. Selection of relevant information sources (biomedical databases)
5. Definition of search strategies
6. Conducting literature search in selected biomedical databases
7. Conducting of additional, non-systematic, literature search in identified systematic reviews
8. Final quality control if necessary.

For details on work steps 1.-6., see the additional file A.2.

**[2] Work steps of article selection and analysis**

1. Import of the results ("hits") from databases into literature management program, and definition of further categories according to stage of selection process (i.e., "1 after search", "2 after duplicate removal", "3 after TiAb screening", and "4 after full-text screening")
2. Duplicate removal
3. Definition of inclusion and exclusion criteria for title/abstract (TiAb) screening
4. TiAb screening (by two reviewers independently)
5. Comparison and consensus discussion on the TiAb screening results
6. Identification and loading of full-texts
7. Definition of inclusion and exclusion criteria for full-text screening
8. Full-text screening (by two reviewers independently)
9. Comparison and consensus discussion on the full-text screening results
10. Graphical representation of the literature search results (flow chart)
11. Addition of further relevant studies from any (systematic) reviews identified during the literature search, if applicable
12. Listing of relevant articles (table)
13. Listing of results of the relevant articles (table).

**References**

Perleth M, Zentner A, Gerhardus A, Gibis B, Lühmann D, editors (2014). Health technology assessment: concepts, methods, practice for science and decision making [Health Technology Assessment: Konzepte, Methoden, Praxis für Wissenschaft und Entscheidungsfindung]. 2nd ed. Berlin: Med. Wiss. Verl.-Ges.
