# Supplementary material for: Decision-making regarding dental treatments – What factors matter from patients’ perspective? A systematic review
Source: BMC Oral Health. 2025 Nov 25;26:289. doi: 10.1186/s12903-025-07032-9 (PMC12903421; doi:10.1186/s12903-025-07032-9)
Supplement: Supplementary file 1 — Additional file 1: A1. Guideline on literature search, selection, and analysis. A2. Search strategy. A3. PRISMA checklist. A4. SWiM checklist. A5. Search strings for databases, including hits. A6. Characteristics, factors of choice, and references of included articles (N = 233), sorted by number of identified articles per country (descending) within study designs I–V. A7. Methodological characteristics of included articles (N = 233), and search details. A8. Coding scheme, codebook, and framework, including definitions of excluded and summarized codes. A9. Code definitions. A10. Calculation of ICA and ICR. A11. Quality assessment by MMAT: study design I. A12. Quality assessment by MMAT: study design II. A13. Quality assessment by MMAT: study design III. A14. Quality assessment by MMAT: study design IV. A15. Quality assessment by MMAT: study design V. A16. MMAT assessment results description. [file 12903_2025_7032_MOESM1_ESM.zip › A2_Search_strategy.docx]

**A2.** Search strategy

**I. Search terms for PICO items**

- PICO item P – patients with freedom of choice (factors of choice): patient, patient's, patients, patients', factor*, determinant*, preference*, crucial, decide, deciding, decisive, determine, determining, influence, influencing, preference*
- PICO item I – dental treatment: dental, dentist*, care, device*, diagnostic*, procedure*, product*, service*, technologies, technology, therapeutic*, therapies, therapy, treatment*
- PICO item T – 10 years: *filter applied in database*

Legend: * – truncation

Comment no.1: The potential search term 'method*' (referring to 'dental treatment methods') is not considered, since it might appear in most abstracts (methods section, etc.) resulting in a high number of hits but not being relevant for our search.

Comment no.2: Search terms refer to titles and abstracts via field tags, etc., and are supplemented by keywords (e.g., MeSH terms).

**II. Search strategy adapted to biomedical databases**

***II.1. PubMed (incl. MEDLINE)***

| **#** | **Search string** |
| --- | --- |
| 1 | patient[tiab] OR "patient's"[tiab] OR patients[tiab] OR "patients'"[tiab]" |
| 2 | crucial[tiab] OR decide[tiab] OR deciding[tiab] OR decisive[tiab] OR determine[tiab] OR determining[tiab] OR influence[tiab] OR influencing[tiab] |
| 3 | factor*[tiab] |
| 4 | #2 AND #3 |
| 5 | determinant*[tiab] OR preference*[tiab] |
| 6 | #4 OR #5 |
| 7 | #1 AND #6 |
| 8 | "Patient Preference"[Mesh] |
| 9 | #7 OR #8 |
| 10 | dental[tiab] OR dentist*[tiab] |
| 11 | care[tiab] OR device*[tiab] OR diagnostic*[tiab] OR procedure*[tiab] OR product*[tiab] OR service*[tiab] OR technologies[tiab] OR technology[tiab] OR therapeutic*[tiab] OR therapies[tiab] OR therapy[tiab] OR treatment*[tiab] |
| 12 | #10 AND #11 |
| 13 | "Dental Care"[Mesh] |
| 14 | #12 OR #13 |
| 15 | #9 AND #14 |
| 16 | #9 AND #14 Filters: published in the last *TIME PERIOD* years |

***II.2. The Cochrane Library***

| **#** | **Search string** |
| --- | --- |
| 1 | patient or "patient's" or patients or "patients'":ti,ab,kw (Word variations have been searched) |
| 2 | crucial or decide or deciding or decisive or determine or determining or influence or influencing:ti,ab,kw (Word variations have been searched) |
| 3 | factor*:ti,ab,kw (Word variations have been searched) |
| 4 | #2 and #3 |
| 5 | determinant* or preference*:ti,ab,kw (Word variations have been searched) |
| 6 | #4 or #5 |
| 7 | #1 and #6 |
| 8 | MeSH descriptor: [Patient Preference] explode all trees |
| 9 | #7 or #8 |
| 10 | dental or dentist*:timba (Word variations have been searched) |
| 11 | care or device* or diagnostic* or procedure* or product* or service* or technologies or technology or therapeutic* or therapies or therapy or treatment*:ti,ab,kw (Word variations have been searched) |
| 12 | #10 and #11 |
| 13 | MeSH descriptor: [Dental Care] explode all trees |
| 14 | #12 or #13 |
| 15 | #9 and #14 |
| 16 | Publication Year from *TIME PERIOD* |

***II.3. Web of Science***

| **#** | **Searchstring** |
| --- | --- |
| 1 | TS=(patient OR "patient's" OR patients OR "patients'") |
| 2 | TS= (crucial OR decide OR deciding OR decisive OR determine OR determining OR influence OR influencing) |
| 3 | TS=(factor*) |
| 4 | #3 AND #2 |
| 5 | TS=(determinant* OR preference*) |
| 6 | #5 OR #4 |
| 7 | #6 AND #1 |
| 8 | TS=(dental OR dentist*) |
| 9 | TS=(care OR device* OR diagnostic* OR procedure* OR product* OR service* OR technologies OR technology OR therapeutic* OR therapies OR therapy OR treatment*) |
| 10 | #9 AND #8 |
| 11 | #10 AND #7 |
| 12 | #10 AND #7  Indexes= […], IC Timespan=*TIME PERIOD* |
