# Supplementary material for: Decision-making regarding dental treatments – What factors matter from patients’ perspective? A systematic review
Source: BMC Oral Health. 2025 Nov 25;26:289. doi: 10.1186/s12903-025-07032-9 (PMC12903421; doi:10.1186/s12903-025-07032-9)
Supplement: Supplementary file 1 — Additional file 1: A1. Guideline on literature search, selection, and analysis. A2. Search strategy. A3. PRISMA checklist. A4. SWiM checklist. A5. Search strings for databases, including hits. A6. Characteristics, factors of choice, and references of included articles (N = 233), sorted by number of identified articles per country (descending) within study designs I–V. A7. Methodological characteristics of included articles (N = 233), and search details. A8. Coding scheme, codebook, and framework, including definitions of excluded and summarized codes. A9. Code definitions. A10. Calculation of ICA and ICR. A11. Quality assessment by MMAT: study design I. A12. Quality assessment by MMAT: study design II. A13. Quality assessment by MMAT: study design III. A14. Quality assessment by MMAT: study design IV. A15. Quality assessment by MMAT: study design V. A16. MMAT assessment results description. [file 12903_2025_7032_MOESM1_ESM.zip › A5_Search_strings_for_databases_including_hits.docx]

**A5.** Search strings for databases, including hits

**I Initial search, date: 16.11.2017**

***I.1. PubMed***Time period: 16.11.2007 – 16.11.2017

Following filters were applied: see #16

| **#** | **Search strings** | **Hits** |
| --- | --- | --- |
| 1 | patient[tiab] OR ""patient's""[tiab] OR patients[tiab] OR ""patients'""[tiab]" | 5.711.384 |
| 2 | crucial[tiab] OR decide[tiab] OR deciding[tiab] OR decisive[tiab] OR determine[tiab] OR determining[tiab] OR influence[tiab] OR influencing[tiab] | 2.545.599 |
| 3 | factor*[tiab] | 2.867.391 |
| 4 | #2 AND #3 | 598.084 |
| 5 | determinant*[tiab] OR preference*[tiab] | 326.728 |
| 6 | #4 OR #5 | 898.823 |
| 7 | #1 AND #6 | 287.736 |
| 8 | ""Patient Preference""[Mesh] | 5.639 |
| 9 | #7 OR #8 | 290.740 |
| 10 | dental[tiab] OR dentist*[tiab] | 239.028 |
| 11 | care[tiab] OR device*[tiab] OR diagnostic*[tiab] OR procedure*[tiab] OR product*[tiab] OR service*[tiab] OR technologies[tiab] OR technology[tiab] OR therapeutic*[tiab] OR therapies[tiab] OR therapy[tiab] OR treatment*[tiab] | 8.643.167 |
| 12 | #10 AND #11 | 96.345 |
| 13 | ""Dental Care""[Mesh] | 30.524 |
| 14 | #12 OR #13 | 113.268 |
| 15 | #9 AND #14 | 2.517 |
| 16 | #9 AND #14 Filters: published in the last 10 years | 1.507 |

Additional search, date: 01.10.2018
Time period: 17.11.2017 – 01.10.2018

Following filters were applied: 17.11.2017 – 01.10.2018

| **#** | **Search strings** | **Hits** |
| --- | --- | --- |
| 1 | patient[tiab] OR ""patient's""[tiab] OR patients[tiab] OR "patients'"[tiab] | 335.227 |
| 2 | crucial[tiab] OR decide[tiab] OR deciding[tiab] OR decisive[tiab] OR determine[tiab] OR determining[tiab] OR influence[tiab] OR influencing[tiab] | 159.378 |
| 3 | factor*[tiab] | 185.406 |
| 4 | #2 AND #3 | 42.850 |
| 5 | determinant*[tiab] OR preference*[tiab] | 21.024 |
| 6 | #4 OR #5 | 61.813 |
| 7 | ""Patient Preference""[Mesh] | 150 |
| 8 | #6 OR #7 | 61.849 |
| 9 | dental[tiab] OR dentist*[tiab] | 9.866 |
| 10 | care[tiab] OR device*[tiab] OR diagnostic*[tiab] OR procedure*[tiab] OR product*[tiab] OR service*[tiab] OR technologies[tiab] OR technology[tiab] OR therapeutic*[tiab] OR therapies[tiab] OR therapy[tiab] OR treatment*[tiab] | 507.624 |
| 11 | #9 AND #10 | 5.523 |
| 12 | "Dental Care"[Mesh] | 89 |
| 13 | #11 OR #12 | 5.534 |
| 14 | #1 AND #8 AND #13 | 170 |

***I.2. The Cochrane Library***
Time period: 16.11.2007– 16.11.2017

Following filters were applied: see #16

| **#** | **Search strings** | **Hits** |
| --- | --- | --- |
| 1 | patient or "patient's" or patients or "patients'":ti,ab,kw (Word variations have been searched) | 622.868 |
| 2 | crucial or decide or deciding or decisive or determine or determining or influence or influencing:ti,ab,kw (Word variations have been searched) | 201.654 |
| 3 | factor*:ti,ab,kw (Word variations have been searched) | 198.803 |
| 4 | #2 and #3 | 51.849 |
| 5 | determinant* or preference*:ti,ab,kw (Word variations have been searched) | 15.565 |
| 6 | #4 or #5 | 65.778 |
| 7 | #1 and #6 | 42.569 |
| 8 | MeSH descriptor: [Patient Preference] explode all trees | 629 |
| 9 | #7 or #8 | 42.569 |
| 10 | dental or dentist*:ti,ab,kw (Word variations have been searched) | 21.126 |
| 11 | care or device* or diagnostic* or procedure* or product* or service* or technologies or technology or therapeutic* or therapies or therapy or treatment*:ti,ab,kw (Word variations have been searched) | 769.666 |
| 12 | #10 and #11 | 14.039 |
| 13 | MeSH descriptor: [Dental Care] explode all trees | 620 |
| 14 | #12 or #13 | 14.039 |
| 15 | #9 and #14 | 623 |
| 16 | Publication Year from 2007 to 2017 | 357 |

Additional search, date: 01.10.2018

Time period: 17.11.2017 – 01.10.2018

Following filters were applied: see #16

| **#** | **Search strings** | **Hits** |
| --- | --- | --- |
| 1 | patient or "patient's" or patients or "patients'":ti,ab,kw (Word variations have been searched) | 733.070 |
| 2 | crucial or decide or deciding or decisive or determine or determining or influence or influencing:ti,ab,kw (Word variations have been searched) | 188.136 |
| 3 | factor*:ti,ab,kw (Word variations have been searched) | 212.068 |
| 4 | #2 and #3 | 45.407 |
| 5 | determinant* or preference*:ti,ab,kw (Word variations have been searched) | 18.633 |
| 6 | #4 or #5 | 62.367 |
| 7 | #1 and #6 | 41.233 |
| 8 | MeSH descriptor: [Patient Preference] explode all trees | 638 |
| 9 | #7 or #8 | 41.233 |
| 10 | dental or dentist*:ti,ab,kw (Word variations have been searched) | 30.030 |
| 11 | care or device* or diagnostic* or procedure* or product* or service* or technologies or technology or therapeutic* or therapies or therapy or treatment*:ti,ab,kw (Word variations have been searched) | 924.061 |
| 12 | #10 and #11 | 19.344 |
| 13 | MeSH descriptor: [Dental Care] explode all trees | 586 |
| 14 | #12 or #13 | 19.344 |
| 15 | #9 and #14 | 798 |
| 16 | #15 with Cochrane Library publication date from Nov 2017 to Oct 2018 | 111 |

***I.3. Web of Science***
Time period: 16.11.2007 – 16.11.2017

Following filters were applied: see #12

| **#** | **Search strings** | **Hits** |
| --- | --- | --- |
| 1 | TOPIC: (patient OR "patient's" OR patients OR "patients'") (Indexes=SCI-EXPANDED, SSCI, A&HCI, ESCI Timespan=All years) | 4.944.014 |
| 2 | TOPIC: (crucial OR decide OR deciding OR decisive OR determine OR determining OR influence OR influencing) (Indexes=SCI-EXPANDED, SSCI, A&HCI, ESCI Timespan=All years) | 5.970.572 |
| 3 | TOPIC: (factor*) (Indexes=SCI-EXPANDED, SSCI, A&HCI, ESCI Timespan=All years) | 4.201.779 |
| 4 | #3 AND #2 (Indexes=SCI-EXPANDED, SSCI, A&HCI, ESCI Timespan=All years) | 1.157.013 |
| 5 | TOPIC: (determinant* OR preference*) (Indexes=SCI-EXPANDED, SSCI, A&HCI, ESCI Timespan=All years) | 568.883 |
| 6 | #5 OR #4 (Indexes=SCI-EXPANDED, SSCI, A&HCI, ESCI Timespan=All years) | 1.672.330 |
| 7 | #6 AND #1 (Indexes=SCI-EXPANDED, SSCI, A&HCI, ESCI Timespan=All years) | 345.303 |
| 8 | TOPIC: (dental OR dentist*) (Indexes=SCI-EXPANDED, SSCI, A&HCI, ESCI Timespan=All years) | 164.714 |
| 9 | TOPIC: (care OR device* OR diagnostic* OR procedure* OR product* OR service* OR technologies OR technology OR therapeutic* OR therapies OR therapy OR treatment*) (Indexes=SCI-EXPANDED, SSCI, A&HCI, ESCI Timespan=All years) | 11.326.551 |
| 10 | #9 AND #8 (Indexes=SCI-EXPANDED, SSCI, A&HCI, ESCI Timespan=All years) | 70.572 |
| 11 | #10 AND #7 (Indexes=SCI-EXPANDED, SSCI, A&HCI, ESCI Timespan=All years) | 2.486 |
| 12 | #10 AND #7  (Indexes=SCI-EXPANDED, SSCI, A&HCI, ESCI Timespan=2007-2017) | 1.818 |

Additional search, date: 01.10.2018
Time period: 17.11.2017 – 01.10.2018

Following filters were applied: Timespan=2017 – 2018

| **#** | **Search strings** | **Hits** |
| --- | --- | --- |
| 1 | TOPIC: (patient OR "patient's" OR patients OR "patients'") ( Indexes=[…] Timespan=2017-2018) | 632.702 |
| 2 | TOPIC: (crucial OR decide OR deciding OR decisive OR determine OR determining OR influence OR influencing) ( Indexes=[…] Timespan=2017-2018) | 797.254 |
| 3 | TOPIC: (factor*) ( Indexes=[…] Timespan=2017-2018) | 567.295 |
| 4 | #3 AND #2 ( Indexes=[…] Timespan=2017-2018) | 175.699 |
| 5 | TOPIC: (determinant* OR preference*) ( Indexes=[…] Timespan=2017-2018) | 81.311 |
| 6 | #5 OR #4 ( Indexes=[…] Timespan=2017-2018) | 247.390 |
| 7 | #6 AND #1 ( Indexes=[…] Timespan=2017-2018) | 50.921 |
| 8 | TOPIC: (dental OR dentist*) ( Indexes=[…] Timespan=2017-2018) | 20.823 |
| 9 | TOPIC: (care OR device* OR diagnostic* OR procedure* OR product* OR service* OR technologies OR technology OR therapeutic* OR therapies OR therapy OR treatment*) ( Indexes=[…] Timespan=2017-2018) | 1.605.391 |
| 10 | #9 AND #8 ( Indexes=[…] Timespan=2017-2018) | 11.571 |
| 11 | #10 AND #7 ( Indexes=[…] Timespan=2017-2018) | 453 |

**II Update search, date: 19.01.2021**

***II.1. PubMed***
Time period: 01.10.2018 – 19.01.2021

Following filters were applied: see #16

| **#** | **Search strings** | **Hits** |
| --- | --- | --- |
| 1 | patient[tiab] OR "patient's"[tiab] OR patients[tiab] OR "patients'"[tiab] | 6.942.275 |
| 2 | crucial[tiab] OR decide[tiab] OR deciding[tiab] OR decisive[tiab] OR determine[tiab] OR determining[tiab] OR influence[tiab] OR influencing[tiab] | 3.117.135 |
| 3 | factor*[tiab] | 3.538.633 |
| 4 | #2 AND #3 | 753.196 |
| 5 | determinant*[tiab] OR preference*[tiab] | 401.861 |
| 6 | #4 OR #5 | 1.121.565 |
| 7 | #1 AND #6 | 367.067 |
| 8 | "Patient Preference"[Mesh] | 9.034 |
| 9 | #7 OR #8 | 371.562 |
| 10 | dental[tiab] OR dentist*[tiab] | 278.522 |
| 11 | care[tiab] OR device*[tiab] OR diagnostic*[tiab] OR procedure*[tiab] OR product*[tiab] OR service*[tiab] OR technologies[tiab] OR technology[tiab] OR therapeutic*[tiab] OR therapies[tiab] OR therapy[tiab] OR treatment*[tiab] | 10.541.835 |
| 12 | #10 AND #11 | 118.419 |
| 13 | "Dental Care"[Mesh] | 33.374 |
| 14 | #12 OR #13 | 136.196 |
| 15 | #9 AND #14 | 3.308 |
| 16 | #9 AND #14 Filters: from 2018/10/01 – 2021/01/19 | 615 |

***II.2. Cochrane Library***
Time period: 10.2018 – 01.2021

Following filters were applied: see #16

| **#** | **Search strings** | **Hits** |
| --- | --- | --- |
| 1 | patient or "patient's" or patients or "patients'":ti,ab,kw (Word variations have been searched) | 989.967 |
| 2 | crucial or decide or deciding or decisive or determine or determining or influence or influencing:ti,ab,kw (Word variations have been searched) | 355.617 |
| 3 | factor*:ti,ab,kw (Word variations have been searched) | 256.377 |
| 4 | #2 and #3 | 58.364 |
| 5 | determinant* or preference*:ti,ab,kw (Word variations have been searched) | 24.746 |
| 6 | #4 or #5 | 2.386 |
| 7 | #1 and #6 | 1.564 |
| 8 | MeSH descriptor: [Patient Preference] explode all trees | 765 |
| 9 | #7 or #8 | 2.260 |
| 10 | dental or dentist*:ti,ab,kw (Word variations have been searched) | 37.747 |
| 11 | care or device* or diagnostic* or procedure* or product* or service* or technologies or technology or therapeutic* or therapies or therapy or treatment*:ti,ab,kw (Word variations have been searched) | 1.252.319 |
| 12 | #10 and #11 | 25.257 |
| 13 | MeSH descriptor: [Dental Care] explode all trees | 634 |
| 14 | #12 or #13 | 25.257 |
| 15 | #9 and #14 | 82 |
| 16 | Publication Year from 2018 to 2021 | 19 |

***II.3. Web of Science***
Time period: 2018 – 2021

Following filters were applied: see #12

Indexes: Indexes=SCI-EXPANDED, SSCI, A&HCI, CPCI-S, CPCI-SSH, BKCI-S, BKCI-SSH, ESCI, CCR-EXPANDED, IC Timespan=All years

| **#** | **Search strings** | **Hits** |
| --- | --- | --- |
| 1 | TS=(patient OR "patient's" OR patients OR "patients'") | 6.428.807 |
| 2 | TS= (crucial OR decide OR deciding OR decisive OR determine OR determining OR influence OR influencing) | 8.041.993 |
| 3 | TS=(factor*) | 5.601.571 |
| 4 | #3 AND #2 | 1.600.392 |
| 5 | TS=(determinant* OR preference*) | 785.156 |
| 6 | #5 OR #4 | 2.306.034 |
| 7 | #6 AND #1 | 457.688 |
| 8 | TS=(dental OR dentist*) | 212.962 |
| 9 | TS=(care OR device* OR diagnostic* OR procedure* OR product* OR service* OR technologies OR technology OR therapeutic* OR therapies OR therapy OR treatment*) | 15.513.341 |
| 10 | #9 AND #8 | 97.602 |
| 11 | #10 AND #7 | 3.536 |
| 12 | #10 AND #7  Indexes= […], IC Timespan=2018-2021 | 961 |

TS=Topic
