# Supplementary material for: Decision-making regarding dental treatments – What factors matter from patients’ perspective? A systematic review
Source: BMC Oral Health. 2025 Nov 25;26:289. doi: 10.1186/s12903-025-07032-9 (PMC12903421; doi:10.1186/s12903-025-07032-9)
Supplement: Supplementary file 1 — Additional file 1: A1. Guideline on literature search, selection, and analysis. A2. Search strategy. A3. PRISMA checklist. A4. SWiM checklist. A5. Search strings for databases, including hits. A6. Characteristics, factors of choice, and references of included articles (N = 233), sorted by number of identified articles per country (descending) within study designs I–V. A7. Methodological characteristics of included articles (N = 233), and search details. A8. Coding scheme, codebook, and framework, including definitions of excluded and summarized codes. A9. Code definitions. A10. Calculation of ICA and ICR. A11. Quality assessment by MMAT: study design I. A12. Quality assessment by MMAT: study design II. A13. Quality assessment by MMAT: study design III. A14. Quality assessment by MMAT: study design IV. A15. Quality assessment by MMAT: study design V. A16. MMAT assessment results description. [file 12903_2025_7032_MOESM1_ESM.zip › A6_Characteristics_factors_of_choice_and_references.docx]

**A6.** Characteristics, factors of choice, and references of included articles (N = 233), sorted by number of identified articles per country (descending) within study designs I-V

| **No.** | **Author (year)** | **Country^1^ / health sector^2^ /**  **institutional setting^3^ [location^4^]** | **Study population (no.)^5^** | **Sociodemographic characteristics** | | | **Treatment category^12^: dental treatment or service** | **Factors of choice & framework codes^13^** |
| --- | --- | --- | --- | --- | --- | --- | --- | --- |
|  |  |  |  | **Age^6, 7^ / range^7, 8^ [years]** | **Female (%)** | **Income^9^ / education^10^ / health status^11^** |  |  |
| **I Qualitative studies** | | | | | | | | |
| I.1 | Al-Moghrabi et al. (2019) [1] | UK / ns / dental service institution [non-clinical area] | patients (n=15) | ns / 19-30 | n=10 (67%) | ns / ns / ns | dental treatment: vacuum-formed retainers | negative results afterwards, quality of life, self-directed wear |
| I.2 | Borreani et al. (2010) [2] | UK / ns / ns | patients (n=39) | group: 75-84 / 65-85+ | ns | ns / ns / ns | dental care in general: oral health services care for older people | health perceptions |
| I.3 | Ellis et al. (2011) [3] | UK, Canada / ns / non-private [non-clinical meeting room] | patients (n=21) | ns / 55-80 | n=17 (81%) | ns / ns / ns | dental treatment: implant supported mandibular overdentures | age-related concerns, early complications, later complications, pain, pain experience, wearing result |
| I.4 | Exley et al. (2012) [4] | UK / private / private [home], non-private [university, work] | patients (n=27) | ns / 23-84 | ns | ns / ns / ns | dental treatment: dental implant | aesthetics, cost, functional, perceived status of treatment, self-perceived need |
| I.5 | Grey et al. (2013) [5] | UK / private / dental practice | patients (n=9) | mean: 60 / 49-69 | n=6 (67%) | ns / ns / ns | dental treatment: dental implants | appearance of social self, appearance of teeth, functionality, limitations in eating and speaking |
| I.6 | Hanefeld et al. (2015) [6] | UK / private / non-private [research location] | patients (n=77) | ns / ns | ns | ns / ns / ns | dental care in general: medical tourism | availability, cost, culture, dissatisfaction, distrust in care, expertise, holiday, low quality, personal networks |
| I.7 | Kashbour et al. (2018) [7] | UK / ns / ns | patients (n=34) | group: >40 / groups: <40->40 | n=22 (65%) | unclear / ns / unclear | dental treatment: dental implant treatment | expectation, family and friends, function, information on hygiene and restorative care, information on internet, longevity, successfulness |
| I.8 | Ke et al. (2013) [8] | UK / ns / in-private | caregivers (of children) (n=16) | group: 30-34, 35-39 / groups: 30-54 | n=12 (75%) | ns / ns / ns | dental treatment: centralized cleft services | continuity of care, cost, facilities at medical centers, information, staff attitudes |
| I.9 | Scott et al. (2009) [9] | UK / ns / in-private | patients (n=57) | mean: 54 / 18+ | n=46 (81%) | ns / unclear / ns | dental treatment: detection interventions (dental cancer) | access, appropriate timing, duration, early diagnosis, negative attitudes, pain, resolve uncertainty, self-diagnosis, symptom interpretations, worry/concern about symptoms |
| I.10 | Serban et al. (2019) [10] | UK / public / (dental) clinic/hospital | patients (n=21) | mean: 61 / 37-86 | n=15 (71%) | ns / ns / unclear | dental care in general: oral health services | acceptance, coping strategies, cost, delay of treatment, experiences, flare-ups, frustration and depression, impact on social life, impact on work life, interactions with other medications and side effects, mobility, self-decision |
| I.11 | Thompson et al. (2020) [11] | UK / ns / dental practice | patients (n=28) | ns / ns | n=37 (49%) (FU: n=10) (ns) | ns / ns / ns | dental care in general: urgent dental care | access, antibiotic beliefs, antibiotic goal, beliefs about procedures, communication/negotiation, costs, delaying tactic, emotional attachment, engagement in consent, family, friends and colleagues, fear about outcome, feelings about dentistry, information and advice, medicines knowledge, minimize disruption, pain relief goal, previous experience, trust, understanding the condition |
| I.12 | van der Zande et al. (2021) [12] | UK / public / (dental) clinic/hospital, dental practice | patients (n=97 (FU: 19)) | group: <29 (FU: <29) / <29-60+ (FU: <29-60+) | n=41 (42%) | ns / ns / ns | dental care in general: dental visiting | available resources, dental anxiety and fear, embarrassment, importance of oral health, trust in dentists, afraid to ask the dentist what will happen, atmosphere of dental waiting rooms and offices |
| I.13 | Bohn et al. (2018) [13] | USA / ns / academic institution (dental department) | patients (n=25) | group: 50-64 / 22-89 | n=19 (76%) | high / college or university degree / ns | dental treatment: mobile apps as dental patient educational aids | appearance, presentation of treatment, usability |
| I.14 | Cohen et al. (2007) [14] | USA / ns / non-private [research location] | patients (n=66) | ns / <25-65+ | n=44 (67%) | low / college or university degree / fair or poor | dental care in general: toothache pain | dental problem, fear of needles, financial reasons, intense pain, lack of access, language barriers, not have the ability to take time off from work, not seeking dental care, pain, problems with transportation, racism |
| I.15 | Dodd et al. (2014) [15] | USA / ns / ns | patients (n=100) | ns / 10-18 | n=48 (48%) | unclear / ns / ns | dental treatment: preventive care, care-seeking behaviors | availability of caregiver, cost, fear, insurance, self-perception of health |
| I.16 | Gatten et al. (2011) [16] | USA / ns / ns | patients (n=37) | 57 / ns | n=17 (46%) | ns / ns / ns | dental treatment: endodontic treatment, prosthesis | additional costs, aesthetics, cleaning/maintenance, comparison to other teeth, cost, family/peer influence, follow-up dental visits, functionality, importance of overall health, insurance, keeping teeth in general, length of treatment, open mouth in chair process, oral hygiene prevention, permanency of treatment, physical disability, physical pain (after treatment), physical pain of procedure, psychological discomfort, reduction of prior pain |
| I.17 | Hoeft et al. (2011) [17] | USA / ns / in-private | parents (of children) (n=48) | 31 / ns | n=48 (100%) | low / unclear / ns | dental care in general: dental visit of children | check-up, other, pain, pediatrician advice, prevention, problem (reactive), school requirement |
| I.18 | Horton et al. (2009) [18] | USA / ns / ns | caregivers (of children) (n=26) | 30 / 19-47 | n=26 (100%) | low / ns / ns | dental treatment: dental treatment of children | recognizing a problem, self-perceived need |
| I.19 | Siegel et al. (2012) [19] | USA / public, private / non-private [research location] | patients (n=118) | 46 / 18-79 | n=69 (58%) | low / unclear / ns | dental care in general: oral healthcare | fear, mistreatment, quality care |
| I.20 | Brown et al. (2020) [20] | India / ns / ns | patients (n=53) | group: 50-64 / groups: 20-70 | n=21 (ns) | ns / ns / ns | dental care in general: health traveling | after care, appearance, cost, facility conditions, follow-up care, former experiences, issue worsened, quality of care, results, satisfaction, surrounding conditions, unavailability, unresolved problem |
| I.21 | Nogueria et al. (2019) [21] | Brazil / ns / non-private [meeting room] | patients (n=13) | mean: 66 /  54-71 | n=9 (69%) | ns / ns / ns | dental treatment: single‐implant mandibular overdentures | comfort/feeling natural/aesthetics, comorbidities/age, costs, eating habits, fear, hygiene/maintenance, implant surgery experience, impression about care, initial oral condition, interpersonal aspects, lack of information, minor inconveniences, perception of treatment outcomes, post‐surgical perceptions, pre‐surgical feelings/perceptions, satisfaction/gratitude, sense of opportunity, social life changes, trans‐surgical perceptions |
| I.22 | Mostajer Haqiqi et al. (2016) [22] | Canada / ns / (dental) clinic [ED]/ hospital | parents (of children) (n=15) | group: 30-49 (parents), 6-9 (children) / groups: 18-59 (parents), 3-9 (children) | n=10 (67%) (parents), n=6 (40%) (children) | middle / college or university degree (parent/s) / ns | dental care in general: emergency department treatment | access, lack of competency, quality of care, satisfaction, socioeconomic challenges, unavailability of dentist, understanding of health |
| I.23 | Atieh et al. (2016) [23] | New Zealand / ns / academic institution (dental department) | patients (n=15) | group: 56-65 / 36-77 | n=9 (60%) | ns / ns / ns | dental treatment: oral implants | aesthetics, appearance, former experiences, functionality, future treatment needs, longevity, overall impression, self-esteem, type of anesthesia |
| I.24 | Giddings et al. (2008) [24] | New Zealand / ns / ns | patients (n=19) | ns / 65-87 | n=14 (74%) | middle / ns / ns | dental care in general: oral healthcare for older people | access, aesthetics, age, cheewing, comfort, consideration of own preference, cost, experiences, insurance, medical need, quality of care, relationship to dentist, relationship to staff, social isolation, transportation |
| I.25 | Gregory et al. (2012) [25] | New Zealand / ns / ns | patients (n=24) | mean: 71 /  60-93 | ns (63%) | ns / ns / ns | dental care in general: oral health care | access, cost, experiences of success, trust in staff |
| I.26 | McKenzie-Green et al. (2009) [26] | New Zealand / public / dental service institution | patients (n=19) | ns / 65-87 | n=14 (74%) | unclear / ns / ns | dental care in general: oral health care behavior | cost, dental calling ritual, dental going ritual, experiences, fear, prevention, self-weight, social weight |
| I.27 | Osman et al. (2014) [27] | New Zealand / ns / (dental) clinic/hospital | patients (n=16) | mean: 62 /  46-80 | n=3 (19%) | ns / ns / ns | dental treatment: dental implant | experience, implant color, implant material, length of treatment, longevity, metal sensitivity |
| I.28 | Sussex et al. (2010) [28] | New Zealand / ns / ns | patients (n=20) | mean: 84 /  75-101 | n=12 (60%) | ns / ns / ns | dental care in general: dental healthcare | acceptance of treatment, cost, dentist's or doctor's recommendation, economic and social disadvantage, fear, health status, impact of war, importance of professional authority, powerlessness of young women, problem-orientated attendance, role models (parents and community), rural isolation, substitute for technological innovation |
| I.29 | Abrahamsson et al. (2017) [29] | Sweden / public / (dental) clinic, hospital | patients (n=15) | mean: 62 /  27-87 | n=10 (67%) | unclear / school education / unclear | dental treatment: dental implant therapy | cost, excellent work, natural (like own teeth), progress of health, progress of health, unsecure results, worry-free work |
| I.30 | Johannsen et al. (2012) [30] | Sweden / public / (dental) clinic/hospital | patients (n=17) | ns / 46-81 | n=9 (53%) | ns / ns / unclear | dental treatment: dental implants | acceptance, appearance, cost, eating problems, fear, frustration, functionality, new hygiene time consuming, quality of life, shame, social isolation, trist in dentist, unnatural look, worthwhile |
| I.31 | Narby et al. (2012) [31] | Sweden / ns / ns | patients (n=10) | mean: 69 /  54-84 | n=6 (60%) | ns / ns / ns | dental treatment: dental implant | cost, families, fear, feeling of loose dentures, information, lack of taste, possibility to discuss treatment options, skills, trust |
| I.32 | Ostberg et al. (2013) [32] | Sweden / public, private / non-private [research location] | patients (n=20) | ns / 22-70 | n=10 (50%) | ns / ns / ns | dentist & dental practice: dental payment system (fee-for-service, capitation plan) | cost, risk, self-diagnosis, social context |
| I.33 | Canuto et al. (2018) [33] | Australia / public / ns | patients (n=19) | ns / 19-65 | n=0 | ns / ns / ns | dental care in general: primary health care services | cultural inappropriateness, family encouragement, fear of bad news, invincible, limited availability, limited self-knowledge, perceptions, prevention, quality of care, relationship to staff, shame, sickness/unwellness, uncomfortable, undertrained staff, waiting time at clinic, waiting time getting appointment |
| I.34 | Slack-Smith et al. (2010) [34] | Australia / public, private / ns | patients (n=63) | median: 72 (female), 70 (male) / 59-92 | n=46 (73%) | unclear / ns / ns | dental care in general: dental care | accessibility of dental care services, affordability of services, costs, fear, information, knowledge, relationships with service providers |
| I.35 | Azhar et al. (2018) [35] | Malaysia / ns / (dental) clinic/hospital | patients (n=35) | ns / 30-81 | n=17 (49%) | ns / unclear / ns | dental treatment: oral cancer treatment | self-diagnosis, self-medication |
| I.36 | Cronin et al. (2009) [36] | Ireland / public, private / ns | patients (n=22) | ns / 45-75 | n=11 (50%) | ns / ns / ns | dental treatment: partially dentate | aesthetics, cost-benefit, improved appearance, instability, pain, physical function |
| I.37 | Niesten et al. (2013) [37] | Netherlands / ns / in-private [home], dental service institution | patients (n=51) | ns / 65-80+ | n=35 (69%) | ns / ns / unclear | dental care in general: dental visit, oral health care | dentist too far away, experiences, information, motivation, old age, pain, scarce energy, social support |
| I.38 | Vanobbergen (2007) [38] | Belgium / public, private combination (health system) / ns | patients (n=150) | ns / 10-36 | ns | ns / ns / ns | dental care in general: oral healthcare | afraid to ask the dentist what will happen, atmosphere of dental waiting rooms and offices, attractiveness, available dental care services, costs, dentists' lack of responsiveness to patients' concern, fear, finding an appropriate dental office/requesting dental care, importance of oral hygiene, inappropriate transport, knowledge, lack of awareness, lack of information concerning availability of dental care, lack of time, language barriers, limited information gained from health care providers,, motivation, pain, poor communication, quality of access, social rejection, travel and journey time, uncertainty about dental treatments |
| I.39 | Mittal et al. (2019) [39] | Singapore / ns / academic institution (dental department) | patients (n=25) | group: 14 / groups: 65->75 | n=15 (60%) | ns / ns / unclear | dental care in general: dental care service | experiences, fear, life-course perspective, no need perceived, oral health awareness, utilization behaviors |
| I.40 | Munira Hernandez-Santos et al. (2021) [40] | Mexico / public / academic institution (dental department) | patients (n=2) | group: 64, 70 / 64-70 | n=1 (50%) | unclear / unclear / ns | dental care in general: oral health care behavior | alimentation, beliefs, benefits of treatment, cost, education, functionality, habits acquired in childhood, importance, lack of time, meaning of oral health, pre-dental attention, self-diagnosis, self-esteem, social relationship |
| I.41 | Naidu (2012) [41] | Trinidad / public, private / non-private [school] | parents, caregivers (of children) (n=18) | mean: 28 /  23-49 | n=16 (89%) | ns / ns / ns | dental care in general: oral health care of children | chair-side manner of the dentist, environment of the dental clinic, experiences, fear, insurance, pain, time point |
| I.42 | Abd Mutalib et al. (2017) [42] | different English-speaking countries (e.g., Australia) / ns / ns | patients, parents, caregivers of children (n=43) | ns / ns | ns | ns / ns / ns | dental care in general: dental tourism (crown, implant, braces, etc.) | attractions, availability of facilitators, bandwagon effect, cost, insurance, physician’s background, physicians’ expertise |
| **II Quantitative randomized controlled trials - RCTs** | | | | | | | | |
| II.1 | Harris et al. (2020) [43] | UK / public / dental practice | patients (n=138 vs. n=134 vs. n=140 (FU: n=51 vs. n=52 vs. n=50)) | group: 35-64 (FU: 35-64) / groups: 18-65+ (FU: groups: 18-65+) | n=246 (60%) (FU: n=87 (57%)) | middle / school education / unclear | dental treatment: dental check-ups | cost, self-perceived health |
| II.2 | Bender et al. (2007) [44] | USA / private / (dental) clinic/hospital | patients^14, 15^ (n=120) | ns / 18-70+ | n=60 (50%) | ns / ns / ns | dentist & dental practice: dentist | dentist's racial concordance, gender concordance |
| II.3 | Andrade et al. (2013) [45] | Brazil / public / (dental) clinic/hospital | patients (n=31 vs. n=31) | (1) 47; (2) 47 / ns | n=31 (50%) | middle / school education / ns | dental treatment: dental treatment | pain during treatment |
| II.4 | Esfandiari et al. (2009) [46] | Canada / ns / ns | patients (n=13 vs. n=23) | mean: (1) 72, (2) 73 / 68-78, 69-79 | n=17 (47%) | low / school education / ns | dental treatment: implant overdenture | cost, instalments |
| II.5 | Heydecke et al. (2008) [47] | Germany / public / (dental) clinic/hospital | patients^16^ (n=20) | mean: 69 /  50-85 | n=12 (60%) | low / school education / ns | dental treatment: complete dentures | aesthetics, chewing ability, comfort |
| II.6 | Felice et al. (2009) [48] | unclear / ns / dental practice | patients (n=15 vs. n=15) | mean: 56 /  (1) 37-69,  (2) 45-70 | n=17 (57%) | ns / ns / ns | dental treatment: dental implant | pain, treatment duration, unpleasant treatment (instrument) |
| II.7 | McKenna et al. (2016) [49] | Ireland / ns / ns | patients (n=27 vs. n=28) | ns / 65+ | n=31 (56%) | middle / ns / ns | dental treatment: tooth replacement | cost |
| II.8 | Albonni et al. (2021) [50] | Syria / ns / ns | patients^17^ (n=13) | mean: 25 /  16-32 | n=5 (39%) | ns / ns / ns | dental treatment: air polishing | comfort, duration, pain, relief, sensitivity, tooth saving |
| **III Quantitative non-randomized controlled trials – Non-RCTs** | | | | | | | | |
| III.1 | Al Garni et al. (2012) [51] | Saudi Arabia / public, private / ns | patients (n=50 vs. n=50) | ns / ns | n=62 (62%) | high / school education / ns | dental treatment: dental implant | area of the missing tooth, cost, desire to replace the missing teeth, desire to want an implant, self-perceived oral health, time elapsed since extraction |
| III.2 | Atchison et al. (2007) [52] | USA / public / (dental) clinic/hospital | patients (n=98 vs. n=105) | mean: (1) 28, (2) 35 / ns | n=138 (68%) | low / ns / ns | dental treatment: oral surgery | cost, eating difficulties, pain, swelling, temporary nerve problems |
| III.3 | Eyuboglu et al. (2020) [53] | Turkey / ns / ns | patients (n=10 vs. n=1 vs. n=19) | mean: 36 /  18-57 | n=17 (58%) | ns / college or university degree / unclear | dental treatment: endodontic treatment | pain intensity |
| III.4 | Yuzbasioglu et al. (2014) [54] | Turkey / ns / (dental) clinic/hospital | patients^18^ (n=24) | mean: 22 / ns | n=12 (50%) | ns / ns / ns | dental treatment: dental impression technique | difficulty in breathing during impression procedure, feeling taste/smell or voice/heat during impression procedure, gagging reflex during impression procedure, more comfortable, sensitivity during impression procedure, size of instrument, time for procedure |
| III.5 | Köberlein et al. (2011) [55] | Germany / ns / ns | patients (n=704 vs. n=664) | mean: 52 /  30-75 | n=693 (51%) | ns / ns / ns | dental care in general: medical tourism (dentures) | care of staff, cost, dentist relationship, quality of work, self-perceived benefit, self-perceived importance, self-perceived need, waiting time |
| III.6 | Al-Dwairi et al. (2014) [56] | Jordan / ns / ns | patients (n=150 vs. n=150) | ns / 20-70+ | n=119 (40%) | low / unclear / ns | dental treatment: dental Implant | aesthetics, avoidance of damaging adjacent teeth, complicated treatments, cost, function, lengthy treatments, postinsertion complaints, scared of unknown side effects, sources of patient information (relatives and friends, dentists, media, internet), surgical risks |
| III.7 | Al-Quran et al. (2011) [57] | Jordan / ns / ns | patients (n=150 vs. n=50) | mean: 44 /  19-67 | n=121 (61%) | low / school education / ns | dental treatment: tooth replacement | aesthetic reason, attention of others, bad oral hygiene, cost, functional reason, missing tooth should be replaced, neighboring teeth, pain and suffer, phobia, replacement will do periodontal trauma, surgery duration |
| III.8 | Fragouli et al. (2016) [58] | Greece / ns / (dental) clinic/hospital [waiting area] | patients (n=65 vs. n=51) | mean: 47 /  18-85 | n=71 (61%) | ns / ns / ns | dental treatment: rubber dam | duration, experience, painful, uncomfortable |
| III.9 | Re et al. (2018) [59] | unclear / private / (dental) clinic/hospital | patients (n=25 vs. n=25) | mean: 44 /  25-60 | n=20 (40%) | high / ns / ns | dental treatment: dental anesthesia | WTP |
| **IV Quantitative descriptive studies** | | | | | | | | |
| *a. Cross-sectional studies* | | | | | | | | |
| IVa.1 | Austin et al. (2009) [60] | UK / ns / (dental) clinic/hospital [waiting room] | patients (n=1,527) | ns / 18-65+ | n=712 (50%) | ns / ns / ns | dental care in general: dental healthcare | appointment not available, cost, easier to get to than my dentist, for second opinion, insurance, treatment offer, visiting friends/relatives in area |
| IVa.2 | Fenton et al. (2021) [61] | UK / ns / dental practice | patients (n=206) | ns / ns | n=132 (64%) | high / college or university degree / ns | dental treatment: orthodontic treatment | aesthetics, color, cost, experiences, specialist, type of dentist |
| IVa.3 | Furnham et al. (2009) [62] | UK / ns / in-private | patients (n=257) | mean: 43 / ns | n=134 (52%) | low / school education / ns | dentist & dental practice: dentist | dentist age, dentist gender, dentist training location |
| IVa.4 | Geoghegan et al. (2019) [63] | UK / ns / dental practice | patients (=children), parents (n=466) | group: 11-13 / groups: 8->20 | ns | ns / ns / ns | dental treatment: orthodontic treatment | bite, color, crookedness, experiences, gaps, sticking out |
| IVa.5 | Goodwin et al. (2011) [64] | UK / public, private / in-private | patients (n=11,925) | mean: (1) 46, (2) 49 / (1), (2) groups: 16-60+ | n=98 (49%) | ns / ns / ns | dental treatment: dental treatment | been too busy, cost, don’t like dentists, experience, fear, haven’t got round to it, haven’t had the time, insurance, no longer have any natural teeth, no need, not able to find NHS dentist |
| IVa.6 | Hill et al. (2013) [65] | UK / ns / ns | patients (n=10,090) | group: 45-54 / groups: 16-85+ | n=5,590 (55%) | ns / ns / ns | dental care in general: dental care | availability of evening/weekend appointments, convenience of dental practice location, explanation of NHS changes, fear, length of time waiting for a routine appointment, length of time waiting for an urgent appointment, relationship with dentist at last visit, reputation of dentists, standard and quality of care, transport facilities and access |
| IVa.7 | Marshman et al. (2012) [66] | UK / ns / in-private | patients (n=10,864) | mean: 53 / 16-104 | n=6,032 (56%) | ns / ns / (very) good (overall health) | dental care in general: dental services | access, need, prevention |
| IVa.8 | Swami et al. (2011) [67] | UK / ns / academic institution (dental department) | patients (n=161) | mean: 21 / 19-24 | n=92 (57%) | ns / ns / ns | dentist & dental practice: dentist | dentist experience, dentist gender, dentist nationality |
| IVa.9 | Vernazza et al. (2015a) [68] | UK / public, private / ns | patients (n=503) | ns / groups: 16-75+ | n=276 (55%) | low / unclear / ns | dental treatment: treatment of molar teeth with nonvital pulps | cost, experience |
| IVa.10 | Al Shaman et al. (2019) [69] | Saudi Arabia / ns / (dental) clinic/hospital | patients (n=ns) | group: 18-25 / groups: 18->40 | n=243 (58%) | ns / school education / ns | dental treatment: dental implants | aesthetics, age of patient, chewing function, cost, dentist, friends/family, television/internet, newspaper/magazine/articles, satisfaction |
| IVa.11 | Aldaij et al. (2018) [70] | Saudi Arabia / ns / (dental) clinic/hospital | patients (n=100) | group: 31-40 / groups: 31->50 | n=53 (53%) | ns / ns / ns | dental treatment: teeth whitening treatment and others | aesthetics, appearance, teeth color |
| IVa.12 | Al-Hussyeen et al. (2010) [71] | Saudi Arabia / public, private / non-private [school] | patients (n=531) | mean: 14 / 12-15 | n=531 (100%) | ns / school education / (very) good | dental care in general: dental healthcare | convenient appointments, cost, dental pain, far from home, friendly staff, lack of treatment alternative, modern and up-to-date dental clinic, post operative complications, quality, recommended by friend or relative, routine, treatment issues |
| IVa.13 | Ali et al. (2020) [72] | Saudi Arabia / ns / ns | patients (n=250) | ns / ns | n=159 (49%) | ns / college or university degree / ns | dental care in general: dental health care services | attitude of staff, cost, difficulties finding appointments, emergency, fear, lack of time, medical need, no need, past experiences, quality standard of care, transportation |
| IVa.14 | Al-Johany et al. (2010) [73] | Saudi Arabia / ns / (dental) clinic/hospital | patients (n=379) | ns / <30->50 | n=98 (26%) | ns / school education / ns | dental treatment: dental implant | acceptance, alternatives, comfortable feeling, cost, duration, fear, functionality, lack of information, looking more natural, medical need, sources of information |
| IVa.15 | Alsarheed et al. (2011) [74] | Saudi Arabia / ns / non-private [school] | patients (n=583) | ns / 9-12 | n=289 (50%) | ns / school education / ns | dentist & dental practice: dentist appearance | experience, fear, gender of dentist, outfit of dentist |
| IVa.16 | Alshukairy et al. (2020) [75] | Saudi Arabia / ns / (dental) clinic/hospital | caregivers (of children) (n=452) | group: 31-40 / groups: 18->50, groups: 2->12 (children) | n=290 (64%) | ns / college or university degree / ns | dental treatment: general anesthesia during dental procedure | treatment under anesthesia |
| IVa.17 | AlZarea et al. (2017) [76] | Saudi Arabia / public, private / (dental) clinic/hospital | patients (n=892) | ns / 60-80+ | n=430 (48%) | ns / ns / ns | dental care in general: oral healthcare | accessibility, experience, nature of complaint, quality of service |
| IVa.18 | Bagher et al. (2019) [77] | Saudi Arabia / ns / academic institution (dental department) | caregivers (of children) (n=104) | mean: 7 (children) / groups: 2-12 (children) | n=61 (59%) | high / school education (parent/s) / unclear | dental treatment: silver diamine fluoride | cosmetic, pain, teeth location |
| IVa.19 | Bahammam et al. (2019) [78] | Saudi Arabia / ns / (dental) clinic/hospital | patients (n=202) | ns / 9-12 | n=ns (25%) | ns / ns / ns | dentist & dental practice: dentist appearance | dentist attire, dentist professional outfit |
| IVa.20 | Fatani et al. (2016) [79] | Saudi Arabia / ns / (dental) clinic/hospital | patients (n=168) | mean: 30 / ns | n=94 (56%) | high / college or university degree / ns | dental treatment: orthodontic treatment | appearance, cost, social isolation, tooth positioning |
| IVa.21 | Fawaz (2015) [80] | Saudi Arabia / public / (dental) clinic/hospital | patients (n=355) | ns / 20+ | n=121 (34%) | ns / college or university degree / ns | dental treatment: dental implant | aesthetics, cost, difficulty to place, fear, function, not useful, replace missing teeth, risk, risk of surgery, sources of information, success rate |
| IVa.22 | Gaffar et al. (2014) [81] | Saudi Arabia / public [ED] / (dental) clinic/hospital [waiting area] | patients (n=875) | mean: 22 /  18-41+ | n=377 (45%) | ns / ns / fair or poor | dental care in general: dental visit | cost, distance, fear, time |
| IVa.23 | Kakti et al. (2020) [82] | Saudi Arabia / ns / (dental) clinic/hospital | caregivers (of children) (n=523) | ns / groups:  3-12 | ns | ns / ns / ns | dental treatment: dental treatment under general anesthesia | cost effective, dental fear, doctor recommendation, extensive dental work, fear, general health or psychological, lesser waiting, limited resources, others, painful previous, past negative experiences, recommended, trust facilities, younger age oft he child |
| IVa.24 | Madarati et al. (2018) [83] | Saudi Arabia / ns / (dental) clinic/hospital | patients (n=278) | ns / 15-50+ | n=141 (53%) | ns / school education / ns | dental treatment: dental dam | better treatment outcome, easy work, experience, infection control, safe use, time duration of session |
| IVa.25 | Mubaraki et al. (2017) [84] | Saudi Arabia / ns / academic institution (dental department) | patients, caregivers (of children) (n=155) | ns / ns | n=97 (mothers) (ns) | unclear / ns / ns | dental treatment: space maintainer therapy | WTP |
| IVa.26 | Quadri et al. (2018) [85] | Saudi Arabia / ns / (dental) clinic/hospital | patients (n=395) | ns / groups: <15-30+ | ns (55%) | ns / college or university degree / ns | dental care in general: dental healthcare | absence of pain, delay in appointment, fear, not necessary, pain |
| IVa.27 | Sabbagh et al. (2020) [86] | Saudi Arabia / ns / (dental) clinic/hospital | parents (of children) (n=283) | group: 31-40 (parents), mean: 9 (children) / groups: 21-50+ (parents) | n=146 (52%) | middle / college or university degree (parent/s) / ns | dental treatment*: parental separation, dental treatment | comfort, doctor comfort and support dental management, eating, fear, satisfactory, sleeping, social improvement, speed, support dental management |
| IVa.28 | Shabbir et al. (2018) [87] | Saudi Arabia / ns (military hospital) / (dental) clinic/hospital | patients (n=136) | ns / groups: 12-20+ | n=87 (64%) | ns / ns / ns | dental treatment: dental treatment | dislike of treatment, fear of treatment, fear of treatment, forgetfulness, inconvenient timing, school, sickness, unable to get off work/school, unable to get time off work, unable to get transport, unable to get transport |
| IVa.29 | Shahrani et al. (2015) [88] | Saudi Arabia / ns / (dental) clinic/hospital | patients (n=72) | mean: 21 / ns | ns (31%) | ns / ns / ns | dental treatment: orthodontic treatment | academic facilities, advice from others, cost, referral from local dentist, self-motivated |
| IVa.30 | Taibah et al. (2018) [89] | Saudi Arabia / public, private / (dental) clinic/hospital | patients (n=504) | mean: 34 / ns | n=303 (60%) | ns / college or university degree / ns | dentist & dental practice: dentist appearance | altruism, clean professional attire, communication skills, continuous professional development, diagnostic and clinical judgment, documentation, efficient alternative, ethics, hygiene, legal rights information, organization, positive caring attitude, professionalism, proper nonverbal responses, respect to the patient and his/her family, treatment information, trust, understandable easy language, work collaboratively with other medical professionals |
| IVa.31 | Chambers et al. (2019) [90] | USA / ns / academic institution (dental department) | patients (n=352) | mean: 42 / ns | n=ns (ns%) | unclear / college or university degree / unclear | dental treatment: orthodontic care | appearance concerns, availability, can I get an appointment, busy, other things going on in my life, certainty complications will be handled, dental society, specialty recommendation, family dentist recommendation, finding good person to give care, functional concern, physical problems, having own dentist do the work, insurance, good payment plan, online (web) information, other advertising, out-of-pocket cost, pictures of beautiful smiles, ads, possible correction without braces, referral from own dentist, sources of influence, stories, experiences of friends, time involved with treatment |
| IVa.32 | Cohen et al. (2008) [91] | USA / public [ED] / non-private | patients (n=15,394) | ns / 21-65+ | n=10,987 (71%) | low / ns / ns | dental care in general: dental service preference having toothache pain | cost, could not get appointment, did not know dentist to call, fear, insurance, medical need, medicine as alternative, pain |
| IVa.33 | Crystal et al. (2017) [92] | USA / ns / academic institution (dental department), (dental) clinic/hospital | caregivers (of children) (n=120) | group: 41-50 / groups: 20-51+ | n=98 (82%) | high / college or university degree / ns | dental treatment: silver diamine fluoride staining | aesthetics |
| IVa.34 | Flores et al. (2008) [93] | USA / ns / in-private [home] | patients (=children), parents, guardians (of children) (n=102,353) | ns / ns | ns | ns / ns / ns | dental care in general: oral health services | access / knowledge, appointment problems, cost, dentist knowledge, insurance, opening hours, transport |
| IVa.35 | Kelly et al. (2014) [94] | USA / ns / dental practice | parents (of children) (n=77) | ns / groups: 41-45 | n=62 (81%) | ns / ns / ns | dental treatment: orthodontic treatment | dentist age, dentist dress, dentist gender, dentist hair, dentist nametag |

| IVa.36 | Kim et al. (2012) [95] | USA / public, private / in-private [home] | patients (n=221) | mean: 43 / 18-91 | n=136 (63%) | ns / college or university degree / ns | dentist & dental practice: dentist | ability to get appointments at convenient times, accepts credit cards or provides credit, assigned dentist, atmosphere/appearance of the office, attitude/helpfulness of staff, convenient office hours, costs, dental school attended, dentist is a member of a dental association, dentist provides personal attention to diagnosis and aftercare, dentist provides you estimates of fees, dentist uses most up-to-date techniques, dentist’s concern for patients/sensitivity, explanation of treatment, information sources, insurance, location, number of years in experience, office will prepare insurance forms, parking, personal appearance of dentist, professional competence of dentist, quality of care, reputation of the dentist (recommendation), waiting in office, waiting time to get appointments, you participate in the treatment decision |
| --- | --- | --- | --- | --- | --- | --- | --- | --- |
| IVa.37 | Olson et al. (2020) [96] | USA / ns / ns | patients (n=249) | group: 25-34 / groups: 18-65 | n=126 (50%) | unclear / college or university degree / ns | dentist & dental practice: dentist appearance | convenience, cost, customer service, length of treatment, quality of treatment, recommendation from family/friends, relationship with provider |
| IVa.38 | Samuels et al. (2015) [97] | USA / ns / in-private [home] | caregivers (of children) (n=386) | mean: 6 / ns | ns | ns / ns / ns | dental care in general: dental visit of children | conflicting appointments, cost, couldn’t get time off work, didn’t have time, forgot about the appointment, getting to the office, got better, insurance, not feeling well, think was not necessary, took too long, transportation |
| IVa.39 | Vela et al. (2012) [98] | USA / ns / in-private | patients (n=124) | ns / groups: 18+ | ns | ns / ns / ns | dental treatment: root canal treatment | recommendation by dentist, risk |
| IVa.40 | Asokan et al. (2016) [99] | India / ns / non-private [school] | caregivers (of children) (n=1,155) | ns / 9-12 | n=799 (69%) | ns / ns / ns | dentist & dental practice: dentist appearance | experience, fear |
| IVa.41 | Bhatia et al. (2018) [100] | India / ns / (dental) clinic/hospital [waiting area] | parents (of children) (n=294) | ns / 1-18 | n=130 (44%) | ns / ns / ns | dental care in general: dental visit of children | appointment was not with the doctor of choice, cost, fear, forgot, motivation, problem with scheduling the appointment, stuck in traffic, symptoms were better, too ill, waiting time |
| IVa.42 | Garcha et al. (2014) [101] | India / ns / in-private (home), non-private [school, at work] | patients (n=250) | mean: 40 / 35-45 | n=94 (38%) | ns / ns / ns | dental care in general: oral healthcare | cleanliness of the dental clinic, cost, don’t like to go by appointments, don't like get my teeth cleaned, don't like to got to dentist, extracting the teeth will lead to loss of vision, fear, first go to traditional healers, insurance, location of the dental clinic, not available on Sundays, not go alone, only expert, pain, professional advice, specialist, trust, try self-care and home remedy |
| IVa.43 | Jayakaran et al. (2017) [102] | India / ns / academic institution (dental department), (dental) clinic/hospital | patients (n=50) | mean: 8 / 6-10 | n=21 (42%) | ns / ns / ns | dentist & dental practice: dental operatory environment | color of dental operatory, dentist gender, dentist outfit, music in operatory, presence of caregiver, scented operatory, toys in dental operatory, wall design |
| IVa.44 | Kamavaram Ellore et al. (2015) [103] | India / ns / academic institution (dental department) | patients, caregivers (of children) (n=300) | mean: 12 (children), 43 (parents) / 9-13 (children), 29-63 (parents) | ns | ns / ns / ns | dentist & dental practice: dentist appearance | dentist attire, dentist gender, dentist protective gear |
| IVa.45 | Keerthana et al. (2020) [104] | India / ns / academic institution (dental department) | patients (=children) (n=200) | mean: 9 (children) /  6-12 (children) | n=98 (50%) | ns / ns / ns | dentist & dental practice: dentist appearance | dentist’s gender, dentist’s outfit color, dentist's protection looks, experience |
| IVa.46 | Mahajan et al. (2021) [105] | Indien / ns / academic institution (dental department), (dental) clinic/hospital | patients (n=824) | mean: 64, 65 / ns | n=412 (50%) | ns / ns / ns | dental treatment: artificial teeth and denture wearing | color of teeth |
| IVa.47 | Manickam et al. (2010) [106] | India / ns / (dental) clinic/hospital | patients (n=400) | mean: 34 /  18-50 | n=198 (50%) | unclear / unclear / ns | dental treatment: root canal treatment | cost, knowledge, motivation, prevention |
| IVa.48 | Paul et al. (2019) [107] | India / ns / academic institution (dental department) | patients (n=60) | group: 36-59 / groups: 18-60+ | n=30 (50%) | middle / unclear / ns | dental treatment: dental prosthetic treatment | cost, dependent on someone for travel/money, do not feel fit to come for the required number of appointments, do not feel the need for the treatment, do not want to undergo pre-prosthetic treatment, need urgent treatment, not convinced about the treatment plan |
| IVa.49 | Poudyal et al. (2010) [108] | India / public / in-private [home] | patients (n=182) | mean: 37 /  18-70 | ns (66%) | ns / ns / ns | dental care in general: dental healthcare | dental disease will recur, dental diseases are not very serious, dental diseases cannot be treated by anybody, experiences, fear, long distance, no problems in my teeth, no time, painful, unpleasant |
| IVa.50 | Pragati (2010) [109] | India / private / (dental) clinic/hospital | patients (n=200) | ns / groups: 16-80+ | n=80 (40%) | ns / school education / ns | dental treatment: dental implant | cost, not clear about the procedure, sources of information, surgical procedure |
| IVa.51 | Raj (2014) [110] | India / ns / (dental) clinic/hospital | patients (n=249) | mean: 54 / 18-84 | n=110 (44%) | ns / ns / ns | dental treatment: prosthodontics | cost, inadequate, knowledge, not motivated |
| IVa.52 | Ravikumar et al. (2016) [111] | India / ns / (dental) clinic/hospital, non-private [school] | patients (=children) (n=534) | mean: 8 / 6-11 | n=264 (49%) | ns / ns / ns | dentist & dental practice: dentist appearance | dentist attire, dentist gender |
| IVa.53 | Saha et al. (2013) [112] | India / ns / ns | patients (n=483) | ns / groups: 20-40+ | n=193 (40%) | ns / school education / ns | dental treatment: dental implant | complicated treatment, cost, fear, others/different alternative, source of information (dental surgeon, family and friends, media, others), time consuming |
| IVa.54 | Shah et al. (2014) [113] | India / ns / (dental) clinic/hospital | patients (n=300) | ns / 16-60 | n=124 (41%) | ns / school education / ns | dental treatment: dental implant | cost, fear, procedure |
| IVa.55 | Shanmugam et al. (2020) [114] | India / ns / academic institution (dental department) | patients (n=239) | group: 18-27 / groups: 18-47 | n=105 (44%) | ns / ns / ns | dentist & dental practice: orthodontic therapy management, prosthodontic management | attitude towards the tooth reduction, cost, treatment period |
| IVa.56 | Shrirao et al. (2016) [115] | India / ns / ns | patients (n=200) | mean: 44 / 18-88 | n=116 (58%) | ns / school education / ns | dental treatment: prosthetic treatment | cost, do not want, experience, far from my house, fear, feel no need, feel not fit, need urgent/quick treatment, not convinced about the treatment, not have confidence in the dentist, not have time, transportation |
| IVa.57 | Suprakash et al. (2013) [116] | India / ns / ns | patients (n=440) | ns / groups: 15-46+ | n=177 (40%) | ns / school education / ns | dental treatment: dental implant | complexity procedure, cost, duration of treatment, fear |
| IVa.58 | Verma et al. (2012) [117] | India / public/private combination (health system) / in-private | patients (n=203) | mean: 43 / ns | n=104 (51%) | ns / school education / ns | dental care in general: dental healthcare | access, appointments availability, cost, dentist and staff behavior, distance from home, doctors available, emergency service availability, lack of time, motivation, self-medication, waiting time |
| IVa.59 | Feldens et al. (2015) [118] | Brazil / ns / non-private [school] | parents, caregivers (of adolescent children) (n=704) | mean: 13 /  12-13 | n=330 (47%) | ns / school education (parent/s) / ns | dental treatment: orthodontic treatment | appearance, chewing, speaking |
| IVa.60 | Feu et al. (2012) [119] | Brazil / ns / academic institution (dental department) | patients (n=252) | mean: 31 /  17-63 | n=100 (40%) | unclear / ns / ns | dental treatment: orthodontic appliances | aesthetics, cost |
| IVa.61 | Leles et al. (2009) [120] | Brazil / ns / ns | patients (n=165) | mean: 45 /  18-71 | ns (69%) | ns / ns / ns | dental treatment: treatments for partial edentulism | chewing, complexity of treatment, confidence, cost, damage to the remaining teeth, fear of dislodgement, feel regret about treatment, feeling younger, future hygiene, future treatment needs, impacts on general health, improve professional oportunities, natural appearance, need of early replacement, need of periodical follow-up, medical need, mastication, pain, risk, similarity to natural teeth, social relationships, time for completing treatment |
| IVa.62 | Leles et al. (2011) [121] | Brazil / ns / (dental) clinic/hospital | patients (n=112) | mean: 56 /  33-79 | n=56 (50%) | middle / ns / ns | dental treatment: prosthodontic treatment (dentures, overdentures, prostheses) | chewing, complexity of treatment, confidence, cost, damage to the remaining teeth, fear of dislodgement, feel regret about treatment, feeling younger, future hygiene, future treatment needs, impacts on general health, improve professional opportunities, mastication, medical need, natural appearance, need of early replacement, need of periodical follow-up, pain, risk, similarity to natural teeth, social relationships, time for completing treatment |
| IVa.63 | Matsumoto et al. (2017) [122] | Brazil / ns / dental service institution [waiting room, dental office anteroom] | patients (n=521) | ns / 13-60+ | n=249 (48%) | ns / school education / ns | dental care in general: emergency dental care | access, always goes to emergency dental center, appearance and clothing of staff, could not find vacancy, difficult vacancy/delay in care, extraction, incompatible time, information provided, lack of dentist, operational causes, pain, problem not solved at (dental) health unit, service facilities, signs of service areas, site cleanup, unaware of treatment, waited too long and gave up |
| IVa.64 | Oliveira et al. (2013) [123] | Brazil / private, other / dental practice | patients (n=54) | ns / groups: 20-50+ | n=40 (74%) | ns / ns / ns | dental treatment: orthodontic treatment | aesthetics, cost, dentist indication, dentist orientation, doubts on the treatment efficiency, fear, information from dentist, joint discomfort, knowledge, long duration, opinion and support from others, pain, preparation for dental implants, specialist recommendation |
| IVa.65 | Souza et al. (2013) [124] | Brazil / ns / ns | patients (n=75) | ns / 45-67 | n=46 (61%) | ns / ns / ns | dental treatment: orthodontic treatment | aesthetics, occlusal disturbances, pain |
| IVa.66 | Souza et al. (2016) [125] | Brazil / ns / ns | patients (n=60) | ns / 18-25 | n=43 (74%) | ns / unclear / ns | dental treatment: prosthodontic treatment (protheses) | absence of support, adaption with alternative, aesthetics, better chewing, clinical indication, comfort, confidence to chew, cost, cost, disease, facility sanitation, fear, fear of more complex procedure, indication of family, low self-esteem, medical need, most appropriate treatment, other treatment not successful, pain, quality of life, safety, satisfaction, self-esteem, shorter treatment, simplicity, stability of the prosthesis and safety |
| IVa.67 | Vieira et al. (2015) [126] | Brazil / ns / ns | patients (n=225) | mean: 43 /  18-84 | n=117 (52%) | ns / ns / ns | dental treatment: prosthodontic treatment | access, costs, experiences, fear, interpersonal interaction, lack of time, motivation, normative need, professional skills, quality of care, risks, self-perceived need, source of information, specialist, technical qualifications |
| IVa.68 | Gurler et al. (2018) [127] | Turkey / ns / academic institution (dental department), (dental) clinic/hospital | patients (n=506) | group: 20-29 / groups: 14 ->50 | n=313 (62%) | ns / college or university degree / ns | dentist & dental practice: oral and maxillofacial surgeons (dentist appearance) | dentist physically strong, middle aged competent, practitioner’s age (years), practitioner’s ethnicity, practitioner’s gender, practitioner’s religion, suggested by the family or friends, university staff |
| IVa.69 | Kuscu et al. (2009) [128] | Turkey / ns / non-private [school] | patients (n=827) | mean: 11 /  9-14 | n=407 (49%) | ns / ns / ns | dentist & dental practice: dentist appearance | dentist’s attire, fear |
| IVa.70 | Ozdogan et al. (2019) [129] | Turkey / ns / (dental) clinic/hospital | patients (n=132) | mean: 34 / groups: 18-41+ | n=77 (58%) | ns / college or university degree / ns | dental treatment: dental implant treatment | cost, information by dentist, knowledge, source of information |
| IVa.71 | Ozveren et al. (2021) [130] | Turkey / ns / ns | patients (answers by parents) (n=325) | mean: 7 / 0-13 | n=166 (51%) | middle / school education (parent/s) / ns | dental treatment: child's first dental visit | abscess, aphthous ulcers, bruxism, check-up, deciduous teeth delay, dental anomaly, dental caries, dental Trauma, gingival problems, loose teeth, malocclusion, pain, permanent teeth delay |
| IVa.72 | Patır Münevveroğlu et al. (2014) [131] | Turkey / ns / academic institution (dental department) | patients (=children) (n=200) | mean: 9 /  6–12 | n=98 (49%) | ns / ns / ns | dentist & dental practice: dentist appearance | clinic perception (Undecorated or Decorated clinic), dentist gender, dentist outfit, dentist type, fear |
| IVa.73 | Tengilimoglu et al. (2017) [132] | Turkey / ns / ns | patients (n=947) | ns / groups: <21-61+ | n=571 (60%) | low / school education / ns | dentist & dental practice: dentist, dental practice | source of information |
| IVa.74 | Tuncer et al. (2015) [133] | Turkey / ns / (dental) clinic/hospital | patients; parents, guardians, caregiver (if patient <18 years of age) (n=890) | ns / 14-22 | n=274 (patients=children, adolescents) (56%), n=245 (parents) (61%) | ns / school education (parent/s) / ns | dental treatment: orthodontic treatment | academic institution, aesthetics, better alignment, cost, functionality, information, social security, source of information |
| IVa.75 | Uslu et al. (2007) [134] | Turkey / public, private / in-private | patients (n=40) | ns / ns | n=27 (68%) | ns / ns / ns | dental treatment: orthodontic treatment | academic institution, cost, insurance |
| IVa.76 | Yahyaoglu et al. (2018) [135] | Turkey / ns / (dental) clinic/hospital | patients (=children) (n=810) | mean: 9 / 9-12 | n=402 (50%) | ns / ns / ns | dentist & dental practice: dentist appearance | features of treatment clinic, physician’s appearance, protective equipment |
| IVa.77 | Azarpazhooh et al. (2015) [136] | Canada / public, private / in-private | patients (n=1,049) | ns / 18-64 | n=518 (59%) | low / school education / (very) good | dental treatment: treatment for toothache pain | pain |
| IVa.78 | Balevi et al. (2007) [137] | Canada / ns / ns | patients (n=40) | mean: 49 / ns | n=30 (75%) | ns / unclear / ns | dental treatment: abscessed tooth treatment | aesthetics, cost, durability, intrusiveness of the dental treatment option |
| IVa.79 | Okuda et al. (2021) [138] | Canada / ns / in-private | patients (questions on themselves and their children) (n=1,574) | group: 18-26 / groups: 18-60+ | n=1,173 (75%) | high / college or university degree / ns | dental treatment: orthodontic treatment | convenience, cost, quality of care, risk, specialist, trained dentist |
| IVa.80 | Srivastava et al. (2014) [139] | Canada / ns / ns | patients (n=317) | mean: 41 / ns | n=145 (46%) | middle / college or university degree / (very) good | dental treatment: mandibular overdentures | cost, insurance |
| IVa.81 | Srivastava et al. (2020) [140] | Canada / public, private / ns | patients (n=39) | mean: 34 /  23-54 | n=24 (62%) | middle / college or university degree / ns | dental treatment: overdentures | cost, risk |
| IVa.82 | Hirschfeld et al. (2019) [141] | Germany / public / academic institution (dental department), (dental) clinic/hospital | patients (n=115) | mean: 57 / ns | n=69 (60%) | middle / ns / unclear | dental treatment: orthodontic tooth alignment | cost, information source (Dentist, Orthodontist, Own children, Friends/family, Media/internet), therapy duration |
| IVa.83 | Lamprecht et al. (2020) [142] | Germany / public / academic institution (dental department) | patients (n=189) | mean: 47 / ns | n=103 (55%) | middle / unclear / ns | dentist & dental practice: dentist appearance | additional qualifications, alternative treatment methods, appointment allocation, close to place of residence, comfortable furnishing, connection to public transport, convenient opening hours, dentist answers questions, dentist explains necessary treatments, dentist has mainly positive reviews, dentist is empathetic, dentist offers home visits, dentist offers inexpensive alternative, doctorate, extensive offer of prophylaxis, feeling comfortable in dental practice, freedom of barriers, friendly reception staff, good cooperation with other doctors/specialists, good interaction with children, internet presence, knowledge of foreign languages, modern technical equipment, photograph, practice-related criteria, professional experience, proof/certificate of advanced and further education, social class, sufficient parking facility |
| IVa.84 | Rustemeyer et al. (2007) [143] | Germany / ns / (dental) clinic/hospital, dental practice | patients (n=315) | mean: 55 / ns | n=184 (58%) | ns / ns / ns | dental treatment: dental implants | aesthetics, cost, experiences of others, functional outcomes, longevity, sources of information |
| IVa.85 | Wang et al. (2021) [144] | Germany / private / dental practice | patients (n=95) | mean: 63 /  28-86 | n=57 (60%) | ns / college or university degree / ns | dental treatment: implant placement | satisfaction |
| IVa.86 | Adedapo et al. (2011) [145] | Nigeria / public / (dental) clinic/hospital | patients (n=284) | mean: 36 /  13-78 | n=176 (62%) | low / school education / ns | dental treatment: dental treatment | cost, easy access to modes of non-doctor treatments (e.g., drugs), fear, prompted by family members and friends |
| IVa.87 | Ajayi et al. (2012) [146] | Nigeria / ns / (dental) clinic/hospital [waiting hall] | patients (n=400) | mean: 38 /  16-78 | n=178 (45%) | ns / unclear / ns | dental care in general: oral healthcare | cost, fear of contracting infection, fear of injection, fear of instruments, fear of pain, feel insecure, no dentist around, no time, noise from dental instruments, transport problems, uncomplimentary remarks about dental instrument/ treatment |
| IVa.88 | Ajayi et al. (2021) [147] | Nigeria / ns / (dental) clinic/hospital | patients (n=410) | mean: 37 / groups: <=20->=61 | n=263 (64%) | unclear / college or university degree / ns | dental treatment: dental appearance and tooth color | appearance, satisfaction, tooth color |
| IVa.89 | Gbadebo et al. (2014) [148] | Nigeria / ns / ns | patients (n=199) | mean: 38 /  18-84 | n=106 (53%) | ns / college or university degree / ns | dental treatment: dental implant | cost, knowledge, no reason, takes time |
| IVa.90 | Idowu et al. (2019) [149] | Nigeria / ns / (dental) clinic/hospital | patients (n=468) | mean: 35 / groups: <20->=60 | n=248 (53%) | high / school education / ns | dental treatment: self-medication and dental service | I don’t know where to receive dental treatment, fear of (surgical) dental treatment, I believe the ailment is simple and I need not see a dentist, my house is far to hospital/dental clinic, no money for dental treatment, no time for dental consultation, others, (self-)medication: confidence in chemist and pharmacy |
| IVa.91 | Lawal et al. (2019) [150] | Nigeria / ns / academic institution (dental department), (dental) clinic/hospital | patients (n=292) | mean: 38 / groups: <=34->=65 | n=147 (50%) | ns / college or university degree / ns | dental care in general: oral health-care Information | sources of oral health-care information (dental clinic, school, mass media, church, market, place of work) |
| IVa.92 | Christell et al. (2019) [151] | Sweden / public, private / in-private, clinic | patients (n=144) | group: 50-54 / groups: 50->85 | n=144 (100%) | low / college or university degree / unclear | dentist & dental practice: osteoporosis risk assessment in primary dental care | cost |
| IVa.93 | Balenovic et al. (2019) [152] | Croatia / ns / (dental) clinic/hospital | patients (n=1,730) | group: 21-25 / groups: 1-86+ | n=ns (ns%) | unclear / ns / ns | dental care in general: emergency dental service | medical need, pain |
| IVa.94 | Sever et al. (2019) [153] | Croatia / ns / academic institution (dental department) | patients (n=265) | mean: 37 / ns | ns (61%) | ns / ns / ns | dental treatment: dental treatment | care provider, cost, dental staff behavior, explanation of dental treatment, waiting time in the office |
| IVa.95 | Sever et al. (2020) [154] | Croatia / ns / academic institution (dental department) | patients (n=517) | ns / ns | ns | ns / ns / ns | dental treatment: dental treatment | care provider, cost, dental staff behavior, explanation of dental treatment, waiting time in the office |
| IVa.96 | Spalj et al. (2014) [155] | Croatia / public / ns | patients (n=1,042) | mean: (1) 12, (2) 18 / 12-18 | n=514 (49%) | ns / ns / ns | dental treatment: orthodontic treatment | laughing, referred by professional, self-perceived need, social isolation, speaking |
| IVa.97 | Bahadori et al. (2013) [156] | Iran / ns / ns | patients (n=100) | 32 / 20-50+ | n=35 (35%) | ns / school education / ns | dental treatment: preventive dental service | cost, fear, inconvenience, organization, patient-provider relationship |
| IVa.98 | Moshkelgosha et al. (2013) [157] | Iran / ns / (dental) clinic/hospital | patients (n=190) | group: 15-24 / groups: 15-54 | n=132 (70%) | ns / school education; college or university degree / ns | dental treatment: orthodontic treatments | aligned teeth and elegant smile, appearance of wires and brackets, attracting face, comfortable chewing, speaking, and breathing, fear of side effects, healthy mouth and teeth, high costs, long duration, other reasons |
| IVa.99 | Moshkelgosha et al. (2014) [158] | Iran / ns / (dental) clinic/hospital | patients (n=348) | ns / ns | n=225 (65%) | ns / ns / ns | dental treatment: orthodontic brackets | aesthetics, convenient appointment times, costs, hygienic concerns, poor reputation, quality of treatment, recommended by friends or relatives, short distance from home, unfavorable working hours |
| IVa.100 | Moshkelgosha et al. (2015) [159] | Iran / public / in-private | patients (n=400) | ns / groups: <30->50 | n=297 (74%) | unclear / school education / ns | dental care in general: dental healthcare | WTP |
| IVa.101 | Saadatfar et al. (2021) [160] | Iran / public / (dental) clinic/hospital | parents (of children) (n=290) | group 36-45 / groups: 25-55+ | n=224 (77%) | middle / unclear / ns | dental treatment: preventive and curative dental services | cost, medical need, pain, prevention |
| IVa.102 | Armfield et al. (2013) [161] | Australia / ns / in-private [home] | patients (n=1,036) | mean: 44 /  15-65+ | n=528 (51%) | high / ns / ns | dental care in general: dental visit | cost, fear, inconvenient to get to, lack of time, motivation, self-diagnosis, treatment need |
| IVa.103 | Beresford et al. (2018) [162] | Australia / private / dental practice | patients (n=12) | mean: 69 /  60-81 | n=7 (58%) | ns / ns / unclear | dental treatment: implant overdenture, fixed prothesis | aesthetics, comfort, ease of chewing, ease of cleaning, ease of speaking, functional limitation, handicap, physical disability, physical pain, psychological discomfort, psychological disability, retention, social disability, stability |
| IVa.104 | Luzzi et al. (2008) [163] | Australia / public, private / in-private | patients (n=517) | mean: 55 / ns | ns (60%) | ns / ns / ns | dental care in general: dental healthcare | advice from a dental professional, aesthetics, cost, experience, fear, have teeth cleaned, inconvenient location of dental clinic, long waiting lists, not having choice of dentist, pain, pain, prevent loss of teeth, prevention, receive unnecessary extractions, subjective norms, teeth healthy, wait a long time in the waiting room |
| IVa.105 | Angelis et al. (2020) [164] | Italy / ns / ns | patients (n=122) | mean: 58 /  37-64 | n=81 (66%) | ns / ns / ns | dental treatment: implant dentistry | digital approach, satisfaction, time consuming treatment |
| IVa.106 | Augusti et al. (2014) [165] | Italy / private / in-private | patients (n=107) | mean: 47 /  18-86 | n=59 (55%) | high / school education / (very) health | dental treatment: implant, partial dentures | cost, experiences, fear, information |
| IVa.107 | Re et al. (2017) [166] | Italy / private / (dental) clinic/hospital [comfortable room] | patients (n=103) | groups: 44 / 23-65 | n=51 (50%) | middle / college or university degree / ns | dental treatment: crown, implant | cost |
| IVa.108 | Jaafar et al. (2018) [167] | Malaysia / ns / (dental) clinic/hospital | patients (n=344) | mean: 40 /  18-77 | n=193 (57%) | ns / ns / ns | dental care in general: dental care | check-up, discomfort, pain, special treatment necessary |
| IVa.109 | Kohli et al. (2014) [168] | Malaysia / public, private / (dental) clinic/hospital | patients (n=772) | ns / groups: <20-80+ | n=466 (60%) | ns / college or university degree / ns | dental treatment: dental implant | costs, duration, knowledge, medical need of surgery, source of information |
| IVa.110 | Tin-Oo et al. (2011) [169] | Malaysia / ns / ns | patients (n=235) | mean: 32 /  18-62 | n=165 (70%) | ns / school education / ns | dental treatment: dental treatment to improve aesthetics | appearance |
| IVa.111 | Hansen Edwards (2013) [170] | Norway / public, private / in-private [home], non-private [grocery store] | patients (n=6,465) | group: 45-66 / groups: 16-80+ | n=3,293 (51%) | unclear / school education / ns | dental care in general: dental health care | cost |
| IVa.112 | Nermo et al. (2019) [171] | Norway / public / non-private [school] | patient (n=986) | group: 16 /  15-18 | n=509 (52%) | ns / unclear / ns | dental treatment: dental treatment | anticipated pain, avoidance, control belief, dental status, fear, knowledge, psychological distress, self-motivation, sex, social motivation |
| IVa.113 | Trovik et al. (2012) [172] | Norway / public, private / ns | patients (n=36) | mean: 46 /  29-62 | n=20 (56%) | ns / ns / ns | dental treatment: dental surgery | appearance, eating, functionality |
| IVa.114 | Vika et al. (2008) [173] | Norway / ns / non-private [classroom] | patients (n=1,385) | mean: 18 / groups: 18 | n=770 (56%) | ns / school education / ns | dental treatment: dental treatment | treatment characteristics |
| IVa.115 | Dudea et al. (2012) [174] | Romania / private / dental practice | patients (n=540) | ns / teenagers-60+ | n=325 (60%) | ns / ns / ns | dental treatment: whitening treatment | aesthetics |
| IVa.116 | Tâncu et al. (2019) [175] | Romania / private / (dental) clinic/hospital | patients (n=117) | mean: 41 /  18-81 | n=84 (72%) | ns / college or university degree / ns | dental care in general: dental services | cleanliness, communication availability of the medical staff, cost, courtesy of medical staff, dentist reputation, hygiene and optimal sterilization, location of the dental office, method of payment, professional degree, sources of information, use of state-of-the-art medical devices, website for the dental office |
| IVa.117 | Tudorici et al. (2017) [176] | Romania / ns / ns | patients (n=214) | ns / 21-62 | ns (50%) | ns / unclear / ns | dental treatment: partial edentulism | aesthetics, functionality, speaking |
| IVa.118 | Ungureanu et al. (2015) [177] | Romania / public, private / in-private | patients (n=724) | mean: 47 / groups: 18-60+ | n=453 (63%) | ns / unclear / ns | dentist & dental practice: dentist | dentist’s competence, dentist’s notoriety, dentist’s patience, dentist’s respect for patients, distance from home, pain, quality of equipment in dental office, quality of the service, recommendation by others, respect, schedules are respected |
| IVa.119 | Gao et al. (2020) [178] | China / ns / non-private [school] | patients (n=40,305) | group: 5 / groups: 3-5 | ns (50%) | middle / school education / fair or poor (overall health) | dental care in general: dental services | dental disease was not severe, difficulty of registration, economic issue, fear of infectious diseases, fear of pain, inconvenience, no dental diseases, no dentists nearby, no need to cure primary teeth, no reliable dentists, no time, other reasons, seeing dentists in kindergarten |
| IVa.120 | Leung et al. (2010) [179] | China / ns / (dental) clinic/hospital [quiet room] | patients (n=51) | mean: 39 /  17-71 | n=35 (69%) | low / school education / fair or poor | dental treatment: dental implant | WTP |
| IVa.121 | Zhu et al. (2019) [180] | China / ns / ns | patients (n=576) | mean: 35 / ns | n=282 (49%) | middle / school education / ns | dental care in general: healthcare services | dentist smell |
| IVa.122 | Shanahan et al. (2017) [181] | Ireland / ns / (dental) clinic/hospital | patients, relatives, caregivers (of older people) (n=105) | mean: 80 / ns | n=58 (55%) | ns / school education / unclear | dental treatment: dental treatment | cost, have no problem or need, have no teeth, no access to downstairs surgeries |
| IVa.123 | Al-Batayneh et al. (2019) [182] | Jordan / ns / academic institution (dental department) | patients (=children) (n=476) | mean: 7 /  2–12 | n=221 (46%) | ns / school education; college or university degree / ns | dental treatment: primary teeth treatment | acceptance, pain, patient knowledge, prevention |
| IVa.124 | Vermaire et al. (2012) [183] | Netherlands / ns / (dental) clinic/hospital | parents (of children) (n=290) | ns / 6-12 (children) | n=147 (51%) | unclear / unclear / ns | dental care in general: dental care | WTP |
| IVa.125 | Chebib et al. (2020) [184] | Switzerland / ns / non-private [research institution] | patients (n=89) | median: 73 / 64-91 | ns [(1) 55%, (2) 51%] | ns / ns / ns | dental care in general: dental services | clinic setting, cost, location, specialist, staff, WTT |
| IVa.126 | Meier et al. (2021) [185] | Switzerland / private / dental practice | patients (n=372) | group: 31-40 / 18-79 | n=222 (60%) | ns / ns / ns | dental treatment: dental trauma splint | aesthetics, color of teeth, experience, surface finish |
| IVa.127 | Sendi et al. (2017) [186] | Switzerland / ns / in-private | patients (n=16) | mean: 74 /  58-87 | n=8 (50%) | ns / ns / ns | dental treatment: dental implant | cost |
| IVa.128 | Tianviwat et al. (2008) [187] | Thailand / ns / ns | parents, caregivers (of children) (n=205) | ns / groups: <30-50+ | n=162 (79%) | ns / school education / (very) good - fair or poor (child) | dental care in general: dental care | cost |
| IVa.129 | Tianviwat et al. (2009) [188] | Thailand / ns / ns | parents, caregivers (of children) (n=210) | ns / childrens’ age | ns | ns / ns / ns | dental care in general: dental visit of children | cost |
| IVa.130 | Hof et al. (2014) [189] | Austria / ns / ns | patients (n=150) | mean: 45 /  18-84 | n=84 (56%) | ns / ns / ns | dental treatment: dental implant, bone graft surgery | cost, duration, healing time, medical reasons, risk, time efficiency of treatment, treatment characteristics, treatment success |
| IVa.131 | Pommer et al. (2011) [190] | Austria / ns / in-private [home] | patients (n=1000) | group: 50+ / groups: <30-50+ | n=522 (52%) | low / ns / unclear | dental treatment: dental implants | aesthetics, costs, functionality, less annoying in the mouth, long treatment time, naturality, need of surgery, sources of information |
| IVa.132 | van den Branden et al. (2013) [191] | Belgium / ns / ns | parents (of children) (n=1,157) | mean: 34 /  22-51 | ns (49%) (children) | ns / school education / ns | dental care in general: dental visit of children | age, comfort, fear, lack of time, motivation, recommendation by professional, risk, self-perceived need, source of information |
| IVa.133 | Atanasov et al. (2016) [192] | Bulgaria / public, private / in-private | patients (n=111) | mean: 45 / 18+ | n=64 (58%) | ns / college or university degree / ns | dental treatment: treatment of gap, dentures, bridge, implant | cost |
| IVa.134 | Lalabonova et al. (2015) [193] | Bulgaria / ns / ns | patients (n=174) | mean: 48 /  24-79 | n=86 (49%) | ns / ns / ns | dental treatment: dental implant | cost, duration, fear |
| IVa.135 | Fernandez et al. (2015) [194] | Chile / ns / (dental) clinic/hospital [waiting room] | patients (n=100) | ns / 18-61+ | n=77 (77%) | ns / school education / ns | dental treatment: bone graft | animal testing, discomfort, ethics, natural materials, overall health, pain, religious reasons, risk of disease, risk of infection, simple preference |
| IVa.136 | Rojas-Torres et al. (2019) [195] | Chile / ns / (dental) clinic/hospital | patients (n=53) | mean: 60 / 4  0-85 | n=40 (75%) | ns / ns / ns | dental treatment: denture marking | accuracy, aesthetics, confidentiality, inaccessibility, simplicity, storage of information |
| IVa.137 | Amjad (2014) [196] | Pakistan / ns / (dental) clinic/hospital | patients (n=240) | mean: 42 /  20-65 | n=114 (47%) | ns / ns / ns | dental treatment: dentures | appearance, combination of appearance and function, cost, felt need, function, lack of knowledge, lack of time, source of information, waiting |
| IVa.138 | Saleem et al. (2018) [197] | Pakistan / ns / academic institution (dental department) | patients (n=126) | mean: 40 /  15-80 | n=47 (38%) | ns / ns / ns | dental treatment: dental treatment | alternative treatments, asymptomatic, conveyance issue, cost, delayed appointments, dental negligence, fear, fear of infections, lack of knowledge, lack of time, no importance, self-medication, unsatisfactory |
| IVa.139 | Nair et al. (2016) [198] | Singapore / ns / ns | patients (n=83) | mean: 73 /  60-91 | ns | low / ns / ns | dental treatment: extraction, filling, and cleaning teeth | WTP |
| IVa.140 | Bajrić et al. (2015) [199] | Bosnia and Herzegovina / ns / (dental) clinic/hospital | patients (n=120) | ns / groups: 8, 12, 15 | n=54 (45%) | ns / ns / ns | dental treatment: local anaesthetic application devices | experience, feeling, material, pain, treatment characteristics |
| IVa.141 | Widström et al. (2012) [200] | Finland / public, private / in-private | patients (n=704) | ns / 47-59 | n=430 (61%) | ns / unclear / unclear | dental treatment: filling replacement, urgent dental care | cost |
| IVa.142 | Chau et al. (2014) [201] | Hong Kong / ns / dental service institution [school] | patients (n=1,500) | ns / ns | n=865 (58%) | high / school education (parent/s) / ns | dental care in general: dental care program | cost, perceived need for care |
| IVa.143 | Abdulwahab et al. (2010) [202] | Kuwait / ns / in-private (home) | patients (n=403) | ns / ns | n=242 (60%) | ns / school education / ns | dental care in general: oral healthcare | availability of drug to reduce anxiety/fear, fear |
| IVa.144 | Malak et al. (2021) [203] | Lebanon / public, private / non-private [school] | patients (n=7,902) | mean: 12 /  12-15 | n=4,096 (52%) | ns / ns / ns | dentist & dental practice: dentist appearance | age of dentist, dentist appearance, fear, gender of dentist, noise, open mouth, time consuming |
| IVa.145 | Dalanon et al. (2018) [204] | Philippines / ns / in-private | patients (n=140) | group: 20-30 / groups: <20->40 | n=140 (100%) | middle / college or university degree / ns | dental care in general: dental healthcare | cost |
| IVa.146 | Wedrychowska-Szulc et al. (2010) [205] | Poland / ns / ns | patients (children, adults); parents or caregivers (of children) (n=1,434) | ns / 7-42 (patients), ns (caregiver) | (1) n=422 (56%), (2) n=365 (54%) | ns / ns / ns | dental treatment: orthodontic treatment | appearance, find a better job, functionality, pain, physical, referred by professional, self-diagnosis, social isolation |
| IVa.147 | Tachalov et al. (2021) [206] | Russia / ns / ns | patients (n=706) | group: 18-24 / 18-75 | n=529 (75%) | ns / ns / ns | dental care in general: dental care | acute pain, preventive check-up, routine oral cavity treatment |
| IVa.148 | Park et al. (2021) [207] | South Korea / ns / (dental) clinic/hospital | patients (n=570) | group: 20-29 / groups: <20-60+ | n=339 (59%) | high / school education / ns | dental care in general: dental medical services | accessibility, communication by assistant, communication by doctor, expertise, reliability, satisfaction, tangibility |
| IVa.149 | Awooda et al. (2014) [208] | Sudan / ns / (dental) clinic/hospital [waiting hall] | patients (n=384) | ns / 17-50+ | n=203 (53%) | ns / college or university degree / ns | dental treatment: dental implant | cost, duration, fear, information, medical need |
| IVa.150 | Nyamuryekung’e et al. (2018) [209] | Tanzania / ns (government hospital) / (dental) clinic/hospital | patients (n=1,511) | group: 25-34 / groups: 18-45+ | n=866 (57%) | middle / school education / (very) good (teeth status) | dental treatment: tooth extraction, tooth filling | cost |
| IVa.151 | Bucchi et al. (2019) [210] | different (Chile, France, Italy, Portugal, Spain) / ns / (dental) clinic/hospital | patients (n=330) | group: 46-60 / groups: 18-60+ | n=202 (61%) | ns / unclear / ns | dental treatment: bone grafts | afraid of disease transmission, afraid of pain or discomfort, against the use of animals for human benefit, ethical or moral motivations, preference for natural materials, religious motivations, simple preference |
| IVa.152 | Laothong et al. (2017) [211] | different (Taiwan, Thailand) / ns / ns | patients (n=500) | mean: (1) 26, (2) 25 / ns | (1) n=131 (52%), (2) n=136 (54%) | low / college or university degree / ns | dental treatment: orthodontic treatment | aesthetics, appearance, cost, discomfort, functionality, idol, make brushing difficult, medical need, pain, suggestion (dentist, family), time, way to clinic |
| IVa.153 | Vernazza et al. (2015b) [212] | different (Germany, UK) / public, private / dental practice | patients (n=105) | ns / groups: 40-71+ | ns (66%) | ns / ns / ns | dental treatment: caries prevention | cost |
| IVa.154 | Walshaw et al. (2019) [213] | different (Brazil, UK) / public / (dental) clinic/hospital, dental service institution | patients (n=200) | group: 26-40 / groups: 18-80 | n=137 (69%) | middle / ns / ns | dental treatment: fluoride varnish | WTP |
| IVa.155 | Nalbandian et al. (2009) [214] | unclear / ns / ns | patients (n=96) | ns / ns | ns | ns / ns / ns | dental treatment: veneers | favored composite, repair costs, replacement costs, time, tooth conservation |
| IVa.156 | Re et al. (2016) [215] | unclear / private / (dental) clinic/hospital | patients (n=50) | mean: 44 /  18-73 | n=25 (50%) | high / college or university degree / ns | dental treatment: computerized anesthesia | WTP |
| *b. Longitudinal studies* | | | | | | | | |
| IVb.1 | Tilashalski et al. (2007) [216] | USA / ns / in-private | patients (n=873, FU: n=872) | ns / 45-65+ (FU: ns) | n=489 (56%) | ns / school education / ns | dental treatment: dental treatment | costs, cynicism toward dentists, effectiveness of dental care, importance of dental visits, prevention, quality of recent dental care |
| IVb.2 | Wall et al. (2015) [217] | USA / ns / in-private [home] | ns [n=approx. 10,000; FU: n=approx. 10,000 (each two years)] | ns / groups: 2-65+ | ns | ns / ns / ns | dental care in general: dental care | another dentist recommended not doing, cost, dental office is too far away, did not want to spend the money, expected problem to go away, fear, Insurance, office not open at convenient time, too busy, unable to take time off |
| IVb.3 | Aarabi et al. (2019) [218] | Germany / ns / in-private [home] | patients, caregivers (of older people) (n=362, FU: ns) | mean: 64 /  50-91 | n=210 (58%) | unclear / school education / (very) good - fair or poor | dental care in general: dental visits | cost |
| IVb.4 | Narby et al. (2008) [219] | Sweden / ns / in-private | patients (n=2,383; FU: n=911) | ns / 45-69 (FU: +10) | ns | ns / ns / ns | dental treatment: dental implant | cost, fear, invasiveness, risk |
| **V Mixed-methods studies** | | | | | | | | |
| V.1 | Clarkson et al. (2020) [220] | UK / ns / dental practice | patients [(1) n=648, (2) n=597] | (1) mean: 40, (2) mean: 50 / unclear | (1) n=9 (30%)-70 (63%), (2) n=305 (51%) | low / unclear / (very) good | dental treatment: dental check-ups | cost, medical need, prevention, self-diagnosis |
| V.2 | Harris et al. (2020) [221] | UK / public / dental practice | patients [(1) not relevant; (2.1) n=412 w/ FU: n=153, (2.2) n=391; (3.1) n=368, (3.2) n=30] | (1) not relevant; (2.1) group: 35-64 (FU: 35-64), (2.2) ns; (3.1) group: 40-49, (3.2) median: 50 / (1) not relevant; (2.1) groups: 18->=65, (2.2) ns; (3.1) groups: <5-89, (3.2) 18-70 | (1) not relevant, (2) BL: n=246, FU 12 months n=87; (3) observations: ns, interviews: 19 ((2) BL: 60%, FU 12 months: 57%; (3) observations: ns, interviews: ns%) | middle / (2) school education / unclear | dentist & dental practice: information on oral health risk | understandable information |
| V.3 | Nayee et al. (2015) [222] | UK / public [ED] / (dental) clinic/hospital | patients [(1) n=1,058; (2) n=1,058] | ns / groups: (1), (2) 16-85+ | (1) ns (51%), (2) ns (51%) | ns / ns / ns | dental care in general: emergency dental care | access, appointments availability, cost, fear, pain, patient lack of knowledge, self-perceived need |
| V.4 | Paisi et al. (2020) [223] | UK / public / dental service institution | patients [(1) n=89, (2) n=11] | (1) mean: 38, (2) ns / (1) 20-65, (2) ns | (1) n=27 (30%), (2) ns | ns / ns / ns | dental care in general: dental service | accessing clinic, accessing dental care, cost, flexibility, location of clinic, longer appointments, social competence of dentist, social competence of staff, supportive management, telephone call and SMS appointment reminders |
| V.5 | El-Din (2008) [224] | Saudi Arabia / ns / (dental) clinic/hospital | patients [(1) n=196,761; (2) n=300] | ns / groups: (1) 0-60+, (2) 18-60+ | (1) n=110,876 (56%); (2) ns | ns / ns / ns | dental care in general: dental visit | death in family, diagnose condition, felt better, forgetting the appointment, inconvenient appointment time, Inconvenient day of week, long distance travel, long waiting time at outpatient, post-operative follow-up, unavailable transportation, unwell to come, work commitment |
| V.6 | Milner et al. (2019) [225] | USA / ns / in-private | patients [(1) n=203, (2) n=502) | (1) mean: 37, (2) mean: 35 / ns | (1) n=110 (ns), (2) n=260 (ns) | ns / ns / ns | dental treatment: new robotic technologies in clinical restorative dentistry | WTP |
| V.7 | Papautsky et al. (2021) [226] | USA / ns / in private | patients [(1) n=2,570; (2) n=50] | (1), (2) mean: 37 / (1), (2) 18-84 | n=2,456 (96%), other^19^: n=118 (6%) | ns / unclear / ns | dental care in general: health care services | diagnostic care, fear of SARS-CoV-2 infectionx, health care delays, preventive |
| V.8 | Da Kfouri et al. (2019) [227] | Brazil / ns / ns | patients [(1) n=900, (2) ns] | ns / ns | ns | ns / ns / ns | dentist & dental practice: dentist appearance | accessibility, allows enough time for me to talk about my concerns or problems, care integration, clinical comprehensiveness, community orientation, continuity of care, cultural competence, detailed knowledge of all my health problems, extended comprehensiveness, family orientation, first contact, gender of dentist, information system, person than just as a patient |
| V.9 | Maciel et al. (2017) [228] | Brazil / public / dental service institution | patients, parents [(1) n=1,045; (2) n=29] | ns / (1) 4-8 (children), ns (parents); (2) ns | ns | ns / ns / ns | dental treatment: dental restorations | aesthetics, appearance, material |
| V.10 | Azarpazhooh et al. (2016) [229] | Canada / ns / ns | (1) dental professionals, (2) patients [(1) n=434, (2) n=500] | ns / ns | ns | ns / ns / ns | dental treatment: root canal treatment, tooth extraction, implant, partial prostheses | aesthetic outcome of the treatment, chance of having pain after treatment, communication with dentist, cost, duration of treatment, experience, experiences of others, insurance, longevity, need for surgery to receive the treatment, number of treatment sessions required, pain before seeing a dentist, pain during treatment, retaining your own natural tooth, time off work required, trust |
| V.11 | Schwendicke et al. (2016) [230] | Germany / ns / (dental) clinic/hospital, dental practice | patients [(1) n=12, (2) n=150] | mean: (1) 31, (2) 44 / (1) 21-64, (2) 18-85 | (1) n=5 (42%), (2) n=84 (56%) | ns / (1) unclear; (2) ns / ns | dental treatment: caries treatment | complications, experiences, fear, need, provided information, reliability, risk, trust |
| V.12 | Sever et al. (2018) [231] | Croatia / public, private / (dental) clinic/hospital | patients [(1) n=592, (2) n=265] | mean: (1) ns, (2) 37 / (1) ns, (2) 17-84 | (1) ns, (2) ns (61%) | middle / college or university degree / ns | dental care in general: dental healthcare | complications, cost, fear, longevity, medical need, patient experiences, provided information, reliability, risk, safety, staff behavior, trust, waiting time in-office |
| V.13 | Sonneveld et al. (2013) [232] | Netherlands / ns / ns, in-private [home] | patients [(1) ns; (2) n=3,127] | ns / groups: (1) ns, (2) 16-65+ | (1) ns, (2) ns (59%) | ns / (1) ns, (2) school education / ns | dentist & dental practice: dental practice | access, accessibility by telephone, appointments availability, clarity of responsibilities, cost, dentist rotation, feedback culture, guideline-orientated working, information on dental services, infrastructure, language, patient-orientated opening hours, payment modality, quality assessment culture, quality of care, reminder of check-ups, risk, source of information, specialties in dental practice, staff education, transparency, waiting room facilities, waiting time in-office, working according to professional standard |
| V.14 | Jaapar et al. (2017) [233] | different / private / (dental) clinic/hospital | patients [(1) n=196, (2) n=196] | mean: (1), (2) 42 / (1), (2) 18-73 | (1) n=99 (52%), (2) n=99 (52%) | ns / ns / ns | dental care in general: medical tourism | attractiveness travel/tourism package, availability of information on dental clinics, certification/accreditation scheme, cost, distance to home, ease of payment, family/relatives/friends are here, good reviews by others, high quality/standard of dental care, insurance, language, location of dental clinic, personalized care, qualified and competent dental professionals, referred by professionals, source of information, source of information, specialist services under one dental clinic, use of high technology for dental care |
| **Legend:** ^1^ ranked in order of number of identified articles per country overall, articles focusing "different" countries and leaving country "unclear" given at the end of each study type section; ^2^ sorted into categories by authors: "public", "private", or both; ^3^ sorted into categories by authors: "in-private", "non-private", "dental practice", "(dental) clinic/hospital", "academic institution (dental department)", and "dental service institution";  ^4^ if stated in article; ^5^ differentiation of intervention and control group for study types II, and III; ^6^ mean, median, or majority of study participants in (single value) age group;  ^7^ several numbers mean different groups of study participants or same majority value; ^8^ range of single or age groups values (min.-max.); ^9^ sorted into categories by authors: "low", "middle" and "high" according to stated characteristics of majority of or all study population; ^10^ sorted into categories by authors: with or in "school education", "college or university degree", and "no school education" ~,  ^11^ sorted into categories by authors: "(very) good", and "fair or poor" ~, ^12^  treatment sorted into categories by authors: "dental treatment", "dental care in general", and "dentist & dental practice";  ^13^ extrated from articles, alphabetic order; ^14^ number of participants in intervention groups unclear; ^15^ n=51 participants reported reason of coice; ^16^ conducted as randomized within-subject crossover trial, i.e., no control group; ^17^ conducted as randomized split-mouth clinical trial, i.e., no control group; ^18^ conducted as within-subject trial, i.e., no control group; ^19^ transgender or non-binary individuals (independent from cisgender); BL – baseline; ED – emergency department; FU – follow-up; ns – not stated or unclear; WTT – willingness-to-travel; sorted into treatment categories by authors:  dental treatment, dental care in general, and dentist & dental practice; **other:** decimal places in values were rounded; references in alphabetic order of author names per study design | | | | | | | | |

**References**

1. Al-Moghrabi D, Salazar FBC, Johall A, Fleming PS. Factors influencing adherence to vacuum-formed retainer wear: a qualitative study. Journal of Orthodontics. 2019;46:212–9. doi:10.1177/1465312519851196.

2. Borreani E, Jones K, Scambler S, Gallagher JE. Informing the debate on oral health care for older people: a qualitative study of older people's views on oral health and oral health care. Gerodontology. 2010;27:11–8. doi:10.1111/j.1741-2358.2009.00274.x.

3. Ellis JS, Levine A, Bedos C, Mojon P, Rosberger Z, Feine J, Thomason JM. Refusal of implant supported mandibular overdentures by elderly patients. Gerodontology. 2011;28:62–8. doi:10.1111/j.1741-2358.2009.00348.x.

4. Exley C, Rousseau N, Donaldson C, Steele JG. Beyond price: individuals' accounts of deciding to pay for private healthcare treatment in the UK. BMC Health Serv Res 2012. doi:10.1186/1472-6963-12-53.

5. Grey EB, Harcourt D, O'Sullivan D, Buchanan H, Kilpatrick NM. A qualitative study of patients' motivations and expectations for dental implants. Br Dent J. 2013;214:E1. doi:10.1038/sj.bdj.2012.1178.

6. Hanefeld J, Lunt N, Smith R, Horsfall D. Why do medical tourists travel to where they do? The role of networks in determining medical travel. Social Science & Medicine. 2015;124:356–63. doi:10.1016/j.socscimed.2014.05.016.

7. Kashbour WA, Rousseau NS, Thomason JM, Ellis JS. Provision of information on dental implant treatment: patients' thoughts and experiences. Clin Oral Impl Res. 2018;29:309–19. doi:10.1111/clr.13118.

8. Ke KM, Mackichan F, Sandy JR, Ness AR, Hollingworth W. Parents' perspectives on centralized cleft services for children: the development of a DCE questionnaire. Oral Dis. 2013;19:185–92. doi:10.1111/j.1601-0825.2012.01969.x.

9. Scott SE, Grunfeld EA, Auyeung V, McGurk M. Barriers and triggers to seeking help for potentially malignant oral symptoms: implications for interventions. J Public Health Dent. 2009;69:34–40. doi:10.1111/j.1752-7325.2008.00095.x.

10. Serban S, Dietrich T, Lopez-Oliva I, Pablo P de, Raza K, Filer A, et al. Attitudes towards oral health in patients with rheumatoid arthritis: a qualitative study nested within a randomized controlled trial. JDR Clinical & Translational Research. 2019;4:360–70. doi:10.1177/2380084419833694.

11. Thompson W, McEachan R, Pavitt S, Douglas G, Bowman M, Boards J, Sandoe J. Clinician and patient factors influencing treatment decisions: ethnographic study of Antibiotic prescribing and operative procedures in out-of-hours and general dental practices. Antibiotics 2020. doi:10.3390/antibiotics9090575.

12. van der Zande MM, Exley C, Wilson SA, Harris RV. Disentangling a web of causation: an ethnographic study of interlinked patient barriers to planned dental visiting, and strategies to overcome them. Community Dent Oral Epidemiol. 2021;49:144–57. doi:10.1111/cdoe.12586.

13. Bohn CE, McQuistan MR, McKernan SC, Askelson NM. Preferences related to the use of mobile apps as dental patient educational aids: a pilot study. Journal of Prosthodontics. 2018;27:329–34. doi:10.1111/jopr.12667.

14. Cohen LA, Harris SL, Bonito AJ, Manski RJ, Macek MD, Edwards RR, Cornelius LJ. Coping with toothache pain: a qualitative study of low-income persons and minorities. J Public Health Dent. 2007;67:28–35. doi:10.1111/j.1752-7325.2007.00005.x.

15. Dodd VJ, Logan H, Brown CD, Calderon A, Catalanotto F. Perceptions of oral health, preventive care, and care-seeking behaviors among rural adolescents. J Sch Health. 2014;84:802–9. doi:10.1111/josh.12215.

16. Gatten DL, Riedy CA, Hong SK, Johnson JD, Cohenca N. Quality of life of endodontically treated versus implant treated patients: a university-based qualitative research study. J Endod. 2011;37:903–9. doi:10.1016/j.joen.2011.03.026.

17. Hoeft KS, Barker JC, Masterson EE. Maternal beliefs and motivations for first dental visit by low-income Mexican-American children in California. Pediatr Dent. 2011;33:392–8.

18. Horton S, Barker JC. Rural Mexican immigrant parents' interpretation of children's dental symptoms and decisions to seek treatment. Community Dent Health. 2009;26:216–21.

19. Siegel K, Schrimshaw EW, Kunzel C, Wolfson NH, Moon-Howard J, Moats HL, Mitchell DA. Types of dental fear as barriers to dental care among African American adults with oral health symptoms in Harlem. Journal of Health Care for the Poor and Underserved. 2012;23:1294–309. doi:10.1353/hpu.2012.0088.

20. Brown J, Johnson J, Ozan-Rafferty ME, Sharma M, Barbera S. Internet narratives focused on health travelers' experiences in India: qualitative analysis. J Med Internet Res 2020. doi:10.2196/15665.

21. Nogueira TE, Dias DR, Rios LF, Silva ALM, Jordão LMR, Leles CR. Perceptions and experiences of patients following treatment with single-implant mandibular overdentures: a qualitative study. Clin Oral Implants Res. 2019;30:79–89. doi:10.1111/clr.13394.

22. Mostajer Haqiqi A, Bedos C, Macdonald ME. The emergency department as a 'last resort': why parents seek care for their child's nontraumatic dental problems in the emergency room. Community Dent Oral Epidemiol. 2016;44:493–503. doi:10.1111/cdoe.12239.

23. Atieh MA, Morgaine KC, Duncan WJ. A qualitative analysis on participants' perspectives on oral implants. Clin Oral Implants Res. 2016;27:383–91. doi:10.1111/clr.12558.

24. Giddings L, McKenzie-Green B, Buttle L, Tahana K. Oral healthcare for older people: 'I can't afford not to go to the dentist, but can I afford it?'. N Z Med J. 2008;121:72–9.

25. Gregory J, Thomson WM, Broughton JR, Cullinan MP, Seymour GJ, Kieser JA, et al. Experiences and perceptions of oral health and oral health care among a sample of older New Zealanders. Gerodontology. 2012;29:54–63. doi:10.1111/j.1741-2358.2010.00402.x.

26. McKenzie-Green B, Giddings LS, Buttle L, Tahana K. Older peoples' perceptions of oral health: 'it's just not that simple'. Int J Dent Hyg. 2009;7:31–8. doi:10.1111/j.1601-5037.2008.00328.x.

27. Osman RB, Morgaine KC, Duncan W, Swain MV, Ma S. Patients' perspectives on zirconia and titanium implants with a novel distribution supporting maxillary and mandibular overdentures: a qualitative study. Clin Oral Implants Res. 2014;25:587–97. doi:10.1111/clr.12106.

28. Sussex PV, Thomson WM, Fitzgerald RP. Understanding the 'epidemic' of complete tooth loss among older New Zealanders. Gerodontology. 2010;27:85–95. doi:10.1111/j.1741-2358.2009.00306.x.

29. Abrahamsson KH, Wennström JL, Berglundh T, Abrahamsson I. Altered expectations on dental implant therapy; views of patients referred for treatment of peri-implantitis. Clin Oral Implants Res. 2017;28:437–42. doi:10.1111/clr.12817.

30. Johannsen A, Westergren A, Johannsen G. Dental implants from the patients perspective: transition from tooth loss, through amputation to implants - negative and positive trajectories. J Clin Periodontol. 2012;39:681–7. doi:10.1111/j.1600-051X.2012.01893.x.

31. Narby B, Hallberg U, Bagewitz IC, Soderfeldt B. Grounded theory on factors involved in the decision-making processes of patients treated with implant therapy. Int J Prosthodont. 2012;25:270–8.

32. Ostberg A-L, Ahlstrom B, Hakeberg M. Patients' choice of payment system in the Swedish public dental service. Views on dental care and oral health. Swed Dent J. 2013;37:131–42.

33. Canuto K, Wittert G, Harfield S, Brown A. "I feel more comfortable speaking to a male": Aboriginal and Torres Strait Islander men's discourse on utilizing primary health care services. International journal for equity in health. 2018;17:185. doi:10.1186/s12939-018-0902-1.

34. Slack-Smith L, Lange A, Paley G, O'Grady M, French D, Short L. Oral health and access to dental care: a qualitative investigation among older people in the community. Gerodontology. 2010;27:104–13. doi:10.1111/j.1741-2358.2009.00320.x.

35. Azhar N, Doss JG. Health-seeking behaviour and delayed presentation of oral cancer patients in a developing country: a qualitative study based on the self-regulatory model. Asian Pacific Journal of Cancer Prevention. 2018;19:2935–41. doi:10.22034/apjcp.2018.19.10.2935.

36. Cronin M, Meaney S, Jepson NJA, Allen PF. A qualitative study of trends in patient preferences for the management of the partially dentate state. Gerodontology. 2009;26:137–42. doi:10.1111/j.1741-2358.2008.00239.x.

37. Niesten D, van Mourik K, van der Sanden W. The impact of frailty on oral care behavior of older people: a qualitative study. BMC Oral Health. 2013;13:1–12. doi:10.1186/1472-6831-13-61.

38. Vanobbergen J, van der Beken R, Gyssels L, Roo L de, Willems S. Barriers to oral health care access among socially vulnerable groups: a qualitative study. Journal of Disability and Oral Health. 2007;8:63–9.

39. Mittal R, Wong ML, Koh GC-H, Ong DLS, Lee YH, Tan MN, Allen PF. Factors affecting dental service utilisation among older Singaporeans eligible for subsidized dental care - a qualitative study. BMC Public Health. 2019;19:1075. doi:10.1186/s12889-019-7422-9.

40. Munira Hernandez-Santos D, Fabiola Diaz-Garcia I. Perceptions of two older adults regarding the factors and barriers that influence their oral health care: a case study. The Qualitative Report. 2021;26:246–61. doi:10.46743/2160-3715/2021.4321.

41. Naidu R, Nuun J, Forde M. Oral healthcare of preschool children in Trinidad: a qualitative study of parents and caregivers. BMC Oral Health. 2012;12.

42. Abd Mutalib NS, Soh YC, Wong TW, Yee SM, Yang Q, Murugiah MK, Ming LC. Online narratives about medical tourism in Malaysia and Thailand: a qualitative content analysis. Journal of Travel & Tourism Marketing. 2017;34:821–32. doi:10.1080/10548408.2016.1250697.

43. Harris R, Lowers V, Laverty L, Vernazza C, Burnside G, Brown S, Ternent L. Comparing how patients value and respond to information on risk given in three different forms during dental check-ups: the PREFER randomised controlled trial. BMC. 2020;21:21. doi:10.1186/s13063-019-3824-3.

44. Bender DJ. Patient preference for a racially or gender-concordant student dentist. J Dent Educ. 2007;71:726–45.

45. Andrade EB, Bianchini MA, Lucchiari N, JR. Combine or separate future pain? The impact of current pain on decisions about future dental treatments. PLoS ONE 2013. doi:10.1371/journal.pone.0064057.

46. Esfandiari S, Lund JP, Penrod JR, Savard A, Mark Thomason J, Feine JS. Implant overdentures for edentulous elders: study of patient preference. Gerodontology. 2009;26:3–10. doi:10.1111/j.1741-2358.2008.00237.x.

47. Heydecke G, Vogeler M, Wolkewitz M, Türp JC, Strub JR. Simplified versus comprehensive fabrication of complete dentures: patient ratings of denture satisfaction from a randomized crossover trial. Quintessence Int. 2008;39:107–16.

48. Felice P, Checchi V, Pistilli R, Scarano A, Pellegrino G, Esposito M. Bone augmentation versus 5-mm dental implants in posterior atrophic jaws. Four-month post-loading results from a randomised controlled clinical trial. Eur J Oral Implantol. 2009;2:267–81.

49. McKenna G, Tada S, Woods N, Hayes M, DaMata C, Allen PF. Tooth replacement for partially dentate elders: a willingness-to-pay analysis. J Dent. 2016;53:51–6. doi:10.1016/j.jdent.2016.07.006.

50. Albonni H, Alseirafi W, Tekleh H, Abo Orabi F, Alhaj M, Almasri D, et al. Clinical outcomes of using Erythritol powder by means of air polishing with ultrasonic debridement in the treatment of initial periodontal pockets in hand of dental students: a split-mouth, randomized, comparative, controlled study. Part I. Int J Dent Hyg 2021. doi:10.1111/idh.12519.

51. Al Garni B, Pani SC, Almaaz A, Al Qeshtaini E, Abu-Haimed H, Al Sharif K. Factors affecting the willingness to pay for implants: a study of patients in Riyadh, Saudi Arabia. Dent Res J (Isfahan). 2012;9:719–24.

52. Atchison KA, Gironda MW, Black EE, Schweitzer S, Der-Martirosian C, Felsenfeld A, et al. Baseline characteristics and treatment preferences of oral surgery patients. J Oral Maxillofac Surg. 2007;65:2430–7. doi:10.1016/j.joms.2007.04.011.

53. Eyuboglu TF, Gonenc FI. The effect of pain intensity levels and clinical symptoms on the treatment preferences of patients with endodontically involved teeth: a preliminary cross-sectional study. Eur Oral Res. 2020;54:142–7. doi:10.26650/eor.20200043.

54. Yuzbasioglu E, Kurt H, Turunc R, Bilir H. Comparison of digital and conventional impression techniques: evaluation of patients' perception, treatment comfort, effectiveness and clinical outcomes. BMC Oral Health. 2014;14:10. doi:10.1186/1472-6831-14-10.

55. Köberlein J, Klingenberger D. Foreign dentures and dental tourism – willingness-to-pay and factors influencing the demand for foreign dental prosthesis in Germany [Auslandszahnersatz und Dentaltourismus – Zahlungsbereitschaft und Einflussfaktoren auf die Nachfrage nach ausländischem Zahnersatz in Deutschland]. [Auslandszahnersatz und Dentaltourismus--Zahlungsbereitschaft und Einflussfaktoren auf die Nachfrage nach auslandischem Zahnersatz in Deutschland]. Gesundheitswesen. 2011;73:e111-8. doi:10.1055/s-0030-1254174.

56. Al-Dwairi ZN, El Masoud BM, Al-Afifi SA, Borzabadi-Farahani A, Lynch E. Awareness, attitude, and expectations toward dental implants among removable prostheses wearers. J Prosthodont. 2014;23:192–7. doi:10.1111/jopr.12095.

57. Al-Quran FA, Al-Ghalayini RF, Al-Zu'bi BN. Single-tooth replacement: factors affecting different prosthetic treatment modalities. BMC Oral Health 2011. doi:10.1186/1472-6831-11-34.

58. Fragouli EP, Petridis XM, Georgopoulou MK. Rubber dam acceptance by patients treated by undergraduate and postgraduate students. Endo-Endodontic Practice Today. 2016;10:95–103.

59. Re D, Del Fabbro M, Karanxha L, Augusti G, Augusti D, Fessi S, Taschieri S. Minimally-invasive dental anesthesia: patients' preferences and analysis of the willingness-to-pay index. Journal of Investigative and Clinical Dentistry 2018. doi:10.1111/jicd.12275.

60. Austin R, Jones K, Wright D, Donaldson N, Gallagher JE. Use of the out-of-hours emergency dental service at two south-east London hospitals. BMC Oral Health. 2009;9:19. doi:10.1186/1472-6831-9-19.

61. Fenton GD, Cazaly MHM, Rolland SL, Vernazza CR. Eliciting preferences for adult orthodontic treatment: a discrete choice experiment. Economic Research. 2021:23800844211012670. doi:10.1177/23800844211012670.

62. Furnham A, Swami V. Patient preferences for dentists. Psychology Health & Medicine. 2009;14:143–9. doi:10.1080/13548500802282690.

63. Geoghegan F, Birjandi AA, Xavier GM, DiBiase AT. Motivation, expectations and understanding of patients and their parents seeking orthodontic treatment in specialist practice. Journal of Orthodontics. 2019;46:46–50. doi:10.1177/1465312518820330.

64. Goodwin M, Pretty IA. Estimating the need for dental sedation. 3. Analysis of factors contributing to non-attendance for dental treatment in the general population, across 12 English primary care trusts. Br Dent J. 2011;211:599–603. doi:10.1038/sj.bdj.2011.1053.

65. Hill KB, Chadwick B, Freeman R, O'Sullivan I, Murray JJ. Adult Dental Health Survey 2009: relationships between dental attendance patterns, oral health behaviour and the current barriers to dental care. Br Dent J. 2013;214:25–32. doi:10.1038/sj.bdj.2012.1176.

66. Marshman Z, Porritt J, Dyer T, Wyborn C, Godson J, Baker S. What influences the use of dental services by adults in the UK? Community Dent Oral Epidemiol. 2012;40:306–14. doi:10.1111/j.1600-0528.2012.00675.x.

67. Swami V, McClelland A, Bedi R, Furnham A. The influence of practitioner nationality, experience, and sex in shaping patient preferences for dentists. Int Dent J. 2011;61:193–8. doi:10.1111/j.1875-595X.2011.00056.x.

68. Vernazza CR, Steele JG, Whitworth JM, Wildman JR, Donaldson C. Factors affecting direction and strength of patient preferences for treatment of molar teeth with nonvital pulps. Int Endod J. 2015;48:1137–46. doi:10.1111/iej.12413.

69. Al Shaman LA, Al Shaman RA, Haralur SB. Knowledge and awareness of dental implants among the patients visiting dental hospitals in the Asir region of Saudi Arabia. International Journal of Medical Dentistry. 2019;23:553–64.

70. Aldaij M, Alshehri T, Alzeer A, Alfayez A, Aldrees F, Almuhaya S, et al. Patient patisfaction with dental appearance and treatment desire to improve esthetics. Journal of Oral Health and Community Dentistry. 2018;12:90–5. doi:10.5005/jp-journals-10062-0033.

71. Al-Hussyeen AJA. Factors affecting utilization of dental health services and satisfaction among adolescent females in Riyadh City. Saudi Dent J. 2010;22:19–25. doi:10.1016/j.sdentj.2009.12.004.

72. Ali SAA, Sadatullah S, Ali AB, Elmahdi AE, Ibrahim WSA. Determinants of dental health care seeking behaviour in Aseer province, Kingdom of Saudi Arabia. Annals of Medical and Health Sciences Research. 2020;10:1034–9.

73. Al-Johany S, Al Zoman HA, Al Juhaini M, Al Refeai M. Dental patients’ awareness and knowledge in using dental implants as an option in replacing missing teeth: a survey in Riyadh, Saudi Arabia. Saudi Dent J. 2010;22:183–8. doi:10.1016/j.sdentj.2010.07.006.

74. Alsarheed M. Children's perception of their dentists. Eur J Dent. 2011;5:186–90.

75. Alshukairy H, Alsahwan B, Alkhabbaz M, Alkhwajah M, Bumajdad Z, Alradwan Z. The extent of parents' acceptance with regard to usage of general anaesthesia for their children during dental procedure in Riyadh City. J Evolution Med Dent Sci. 2020;9:3289–93. doi:10.14260/jemds/2020/723.

76. AlZarea BK. Dental and oral problem patterns and treatment seeking behavior of geriatric population. Open Dentistry Journal. 2017;11:230–6. doi:10.2174/1874210601711010230.

77. Bagher SM, Sabbagh HJ, AlJohani SM, Alharbi G, Aldajani M, Elkhodary H. Parental acceptance of the utilization of silver diamine fluoride on their child's primary and permanent teeth. Patient Prefer Adherence. 2019;13:829–35. doi:10.2147/ppa.S205686.

78. Bahammam S. Children's preferences toward dentist attire in Al Madinah Al Munawarah. Patient Prefer Adherence. 2019;13:601–7. doi:10.2147/ppa.S196373.

79. Fatani E, Al-Yousef S. Willingness to pay for orthodontic treatment in Kingdom of Saudi Arabia, Riyadh Province. Saudi J Oral Sci. 2016;3:104. doi:10.4103/1658-6816.188078.

80. Fawaz A. Patients’ awareness of a dental implant as an option for tooth replacement: a survey in Alkharj Province, Saudi Arabia. Int J Dent Oral Health 2015. doi:10.16966/2378-7090.113.

81. Gaffar BO, Alagl AS, Al-Ansari AA. The prevalence, causes, and relativity of dental anxiety in adult patients to irregular dental visits. Saudi Med J. 2014;35:598–603.

82. Kakti A, Almutawa YK, AL-Amoudi SE, Alsulaiman SF, Alghofaily RA. Commonly reported factors influencing the parent's decision to accept dental treatment under general anesthesia for their children. Medical science. 2020;24:4808–12.

83. Madarati A, Abid S, Tamimi F, Ezzi A, Sammani A, Shaar, Mohamad Bachar Abou Al, Zafar M. Dental-dam for infection control and patient safety during clinical endodontic treatment: preferences of dental patients. Int J Environ Res Public Health 2018. doi:10.3390/ijerph15092012.

84. Mubaraki S. Willingness of parents to pay for space maintainer therapy for their children. Advances in Dentistry & Oral Health 2017. doi:10.19080/ADOH.2017.05.5555665.

85. Quadri FA, Am Jafari F, Albeshri AT, Zailai AM. Factors influencing patients' utilization of dental health services in Jazan, Kingdom of Saudi Arabia. Int J Clin Pediatr Dent. 2018;11:29–33. doi:10.5005/jp-journals-10005-1479.

86. Sabbagh HJ, Sijini OT. Parental preference for parental separation and their satisfaction regarding their children dental treatment in pediatric dental clinics in Saudi Arabia. Journal of International Society of Preventive and Community Dentistry. 2020;10:116–23. doi:10.4103/jispcd.JISPCD_280_19.

87. Shabbir A, Alzahrani M, Abu Khalid A. Why do patients miss dental appointments in Eastern Province military hospitals, Kingdom of Saudi Arabia? Cureus. 2018;10:e2355. doi:10.7759/cureus.2355.

88. Shahrani I, Tikare S, Togoo RA, Qahtani F, Assiri K, Meshari A. Patient's satisfaction with orthodontic treatment at King Khalid University, college of dentistry, Saudi Arabia. Bangladesh Journal of Medical Science. 2015;14:146–50. doi:10.3329/bjms.v14i2.17837.

89. Taibah SM. Dental professionalism and influencing factors: patients' perception. Patient Prefer Adherence. 2018;12:1649–58. doi:10.2147/PPA.S172788.

90. Chambers DW, Zitterkopf JG. How people make decisions about whether or not to seek orthodontic care: upstream in the treatment chain. American Journal ofOrthodontics and Dentofacial Orthopedics. 2019;155:826–31. doi:10.1016/j.ajodo.2018.07.019.

91. Cohen LA, Bonito AJ, Akin DR, Manski RJ, Macek MD, Edwards RR, Cornelius LJ. Toothache pain - a comparison of visits to physicians, emergency departments and dentists. Journal of the American Dental Association. 2008;139:1205–16.

92. Crystal YO, Janal MN, Hamilton DS, Niederman R. Parental perceptions and acceptance of silver diamine fluoride staining. J Am Dent Assoc. 2017;148:510-518.e4. doi:10.1016/j.adaj.2017.03.013.

93. Flores G, Tomany-Korman SC. Racial and ethnic disparities in medical and dental health, access to care, and use of services in US children. Pediatrics. 2008;121:e286-98. doi:10.1542/peds.2007-1243.

94. Kelly GR, Shroff B, Best AM, Tufekci E, Lindauer SJ. Parents' preferences regarding appearance and attire of orthodontists. Angle Orthod. 2014;84:404–9. doi:10.2319/071113-510.1.

95. Kim MJ, Damiano PC, Hand J, Denehy GE, Cobb DS, Qian F. Consumers' choice of dentists: how and why people choose dental school faculty members as their oral health care providers. J Dent Educ. 2012;76:695–704.

96. Olson JC, Shroff B, Carrico C, Boyle J, Lindauer SJ. Comparison of patient factors influencing the selection of an orthodontist, general dentist, or direct-to-consumer aligners. American Journal ofOrthodontics and Dentofacial Orthopedics. 2020;157:526-532.e2. doi:10.1016/j.ajodo.2019.11.010.

97. Samuels RC, Ward VL, Melvin P, Macht-Greenberg M, Wenren LM, Yi J, et al. Missed appointments: factors contributing to high no-show rates in an urban pediatrics primary care clinic. Clinical Pediatrics. 2015;54:976–82. doi:10.1177/0009922815570613.

98. Vela KC, Walton RE, Trope M, Windschitl P, Caplan DJ. Patient preferences regarding 1-visit versus 2-visit root canal therapy. J Endod. 2012;38:1322–5. doi:10.1016/j.joen.2012.06.038.

99. Asokan A, Kambalimath HV, Patil RU, Maran S, Bharath KP. A survey of the dentist attire and gender preferences in dentally anxious children. J Indian Soc Pedod Prev Dent. 2016;34:30–5. doi:10.4103/0970-4388.175507.

100. Bhatia R, C Vora E, Panda A. Pediatric dental appointments no-show: rates and reasons. Int J Clin Pediatr Dent. 2018;11:171–6. doi:10.5005/jp-journals-10005-1506.

101. Garcha V, Shetiya SH, Kakodkar P. Barriers to oral health care amongst different social classes in India. Community Dent Health. 2010;27:158–62. doi:10.1922/CDH_2426Garcha05.

102. Jayakaran TG, Rekha CV, Annamalai S, Baghkomeh PN, Sharmin DD. Preferences and choices of a child concerning the environment in a pediatric dental operatory. Dent Res J (Isfahan). 2017;14:183–7. doi:10.4103/1735-3327.208767.

103. Kamavaram Ellore VP, Mohammed M, Taranath M, Ramagoni NK, Kumar V, Gunjalli G. Children and parent's attitude and preferences of dentist's attire in pediatric dental practice. Int J Clin Pediatr Dent. 2015;8:102–7. doi:10.5005/jp-journals-10005-1293.

104. Keerthana R GJ. Assessment of the feelings and attitude of the children towards the dentist. Journal of Research in Medical and Dental Science. 2020;8:84–92.

105. Mahajan N, Kaur S, Suman N. Shade preference of artificial teeth in denture wearing local population. A cross-sectional study. Journal of Clinical and Diagnostic Research 2021. doi:10.7860/jcdr/2021/47447.14449.

106. Manickam S, Selvakumar R, Devadason P. An economic analysis of willingness to pay for root canal treatment for dental caries in Thoothukudi District, Tamilnadu. Journal of the Indian association of public health dentistry. 2010;8:208–14.

107. Paul N, Dhakshaini MR, Swamy RKN, Sowmya S, Ravi MB. An evaluation of factors affecting patient's decision making regarding dental prosthetic treatment. J. Evolution Med. Dent. Sci. 2019;8:3683–7. doi:10.14260/jemds/2019/797.

108. Poudyal S, Rao A, Shenoy R, Priya H. Utilization of dental services in a field practice area in Mangalore, Karnataka. Indian J Community Med. 2010;35:424–5. doi:10.4103/0970-0218.69278.

109. Pragati K, Mayank K. Awareness of dental implants as a treatment modality amongst people residing in Jaipur (Rajasthan). Journal of Clinical and Diagnostic Research. 2010;4:3622–6.

110. Raj N, Reddy N, Japatti S, Thomas M, Uthappa R. Knowledge, attitudes towards prosthodontics rehabilitation and utilization of dental services among Songadh and Amargadh population. J Dent. 2014;3:1–6. doi:10.14303/jdmms.2014.001.

111. Ravikumar D, Gurunathan D, Karthikeyan S, Subbramanian E, Samuel VA. Age and environment determined children's preference towards dentist attire - a Cross - sectional study. J Clin Diagn Res. 2016;10:ZC16-ZC19. doi:10.7860/JCDR/2016/22566.8632.

112. Saha A, Dutta S, Vijaya V, Rajnikant N. Awareness among patients regarding Implants as a treatment option for replacement of missing teeth in Chattisgarh. Journal of International Oral Health. 2013;5:48–52.

113. Shah RJ, Chaturvedi A, Agarwal H. Dental implants as a treatment modality: awareness survey among people of Ahmedabad. International Journal of Prosthodontics and Restorative Dentistry. 2014;4:35–8. doi:10.5005/jp-journals-10019-1103.

114. Shanmugam J, Varghese RM, Suresh V. A retrospective study on the patient's preference on the management of the anterior space by orthodontic therapy or prosthodontic management. Journal of Complementary Medicine Research. 2020;11:203–11. doi:10.5455/jcmr.2020.11.02.28.

115. Shrirao ND, Deshmukh SP, Pande NA, Radke UM. An evaluation of patient's decisions regarding dental prosthetic treatment. J Indian Prosthodont Soc. 2016;16:366–71. doi:10.4103/0972-4052.191287.

116. Suprakash B, Ahammed ARY, Thareja A, Kandaswamy R, Nilesh K, Bhondwe Mahajan S. Knowledge and attitude of patients toward dental implants as an option for replacement of missing teeth. J Contemp Dent Pract. 2013;14:115–8. doi:10.5005/jp-journals-10024-1282.

117. Verma H, Aggarwal AK, Rattan V, Mohanty U. Access to public dental care facilities in Chandigarh. Indian journal of dental research. 2012;23:121. doi:10.4103/0970-9290.99057.

118. Feldens CA, Nakamura EK, Tessarollo FR, Closs LQ. Desire for orthodontic treatment and associated factors among adolescents in southern Brazil. Angle orthodontist. 2015;85:224–32. doi:10.2319/021014-105.1.

119. Feu D, Catharino F, Duplat CB, Capelli Junior J. Esthetic perception and economic value of orthodontic appliances by lay Brazilian adults. Dental Press J. Orthod. 2012;17:102–14. doi:10.1590/S2176-94512012000500015.

120. Leles CR, Martins RR, Silva ET, Nunes MF. Discriminant analysis of patients' reasons for choosing or refusing treatments for partial edentulism. J Oral Rehabil. 2009;36:909–15. doi:10.1111/j.1365-2842.2009.02018.x.

121. Leles CR, Ferreira NP, Vieira AH, Campos ACV, Silva ET. Factors influencing edentulous patients' preferences for prosthodontic treatment. J Oral Rehabil. 2011;38:333–9. doi:10.1111/j.1365-2842.2010.02158.x.

122. Matsumoto MS, Gatti MA, Conti MH de, Ap Simeao SF de, Oliveira Braga Franzolin S de, Marta SN. Determinants of demand in the public dental emergency service. J Contemp Dent Pract. 2017;18:156–61.

123. Oliveira PGdSA, Tavares RR, Freitas JC. Assessment of motivation, expectations and satisfaction of adult patients submitted to orthodontic treatment. Dental Press J Orthod. 2013;18:81–7. doi:10.1590/s2176-94512013000200018.

124. Souza RA de, Oliveira AF de, Pinheiro SMS, Cardoso JP, Magnani MBBdA. Expectations of orthodontic treatment in adults: the conduct in orthodontist/patient relationship. Dental Press J Orthod. 2013;18:88–94. doi:10.1590/s2176-94512013000200019.

125. Souza FI de, Souza Costa A de, Dos Santos Pereira R, Dos Santos PH, Brito RB de, JR, Rocha EP. Assessment of satisfaction level of edentulous patients rehabilitated with implant-supported prostheses. Int J Oral Maxillofac Implants. 2016;31:884–90. doi:10.11607/jomi.4267.

126. Vieira AH, Castro e Silva D, Nogueira TE, Leles CR. Exploring motivations to seek and undergo prosthodontic care: a cross-sectional study in a Brazilian adult sample. Patient Prefer Adherence. 2015;9:803–9. doi:10.2147/PPA.S81645.

127. Gurler G, Delilbasi C, Kacar I. Patients' perceptions and preferences of oral and maxillofacial surgeons in a university dental hospital. Eur Oral Res. 2018;52:137–42. doi:10.26650/eor.2018.483.

128. Kuscu OO, Caglar E, Kayabasoglu N, Sandalli N. Short communication: preferences of dentist's attire in a group of Istanbul school children related with dental anxiety. Eur Arch Paediatr Dent. 2009;10:38–41. doi:10.1007/BF03262666.

129. Ozdogan MS, Gumusok M, Ertem YS, Omeroglu A, Erdem M. Assessment of expectation, attitude and preconceptions in a group patient applying for dental implant treatment. Clinical and Experimental Health Sciences. 2019;9:334–9. doi:10.33808/clinexphealthsci.599952.

130. Ozveren N, Serindere G, Baltaci E. Evaluation of pediatric patient's age, behaviors, and reasons for the first dental visit in Edirne, Turkey: a cross-sectional study. International journal of dental sciences. 2021;23:196–205. doi:10.15517/ijds.2021.45847.

131. Patır Münevveroğlu A, Ballı Akgöl B, Erol T. Assessment of the feelings and attitudes of children towards their dentist and their association with oral health. ISRN Dent. 2014;2014:867234. doi:10.1155/2014/867234.

132. Tengilimoglu D, Sarp N, Yar CE, Bektas M, Hidir MN, Korkmaz E. The consumers' social media use in choosing physicians and hospitals: the case study of the province of Izmir. Int J Health Plann Manage. 2017;32:19–35. doi:10.1002/hpm.2296.

133. Tuncer C, Bavbek CN, Tuncer BB, Bani AA, Celik B. How do patients and parents decide for orthodontic treatment-effects of malocclusion, personal expectations, education and media. Journal of clinical pediatric dentistry. 2015;39:392–9.

134. Uslu O, Akcam MO. Evaluation of long-term satisfaction with orthodontic treatment for skeletal class III individuals. J Oral Sci. 2007;49:31–9. doi:10.2334/josnusd.49.31.

135. Yahyaoglu O, Baygin O, Yahyaoglu G, Tuzuner T. Effect of dentists' appearance related with dental fear and caries status in 6-12 years old children. J Clin Pediatr Dent. 2018;42:262–8. doi:10.17796/1053-4628-42.4.4.

136. Azarpazhooh A, Quinonez C. Treatment preferences for toothache among working poor canadians. J Endod. 2015;41:1985–90. doi:10.1016/j.joen.2015.08.025.

137. Balevi B, Shepperd S. The management of an endodontically abscessed tooth: patient health state utility, decision-tree and economic analysis. BMC Oral Health. 2007;7:17. doi:10.1186/1472-6831-7-17.

138. Okuda BC, Tabbaa S, Edmonds M, Toubouti Y, Saltaji H. Direct to consumer orthodontics: Exploring patient demographic trends and preferences. American Journal ofOrthodontics and Dentofacial Orthopedics. 2021;159:210-216.e2. doi:10.1016/j.ajodo.2019.12.024.

139. Srivastava A, Feine JS, Esfandiari S. Are people who still have their natural teeth willing to pay for mandibular two-implant overdentures? Journal of Investigative and Clinical Dentistry. 2014;5:117–24. doi:10.1111/jicd.12057.

140. Srivastava A, Esfandiari S, Madathil SA, Birch S, Feine JS. Willingness to pay for mandibular overdentures: a societal perspective. JDR Clin Trans Res. 2020;5:30–9. doi:10.1177/2380084419849870.

141. Hirschfeld J, Reichardt E, Sharma P, Hilber A, Meyer-Marcotty P, Stellzig-Eisenhauer A, et al. Interest in orthodontic tooth alignment in adult patients affected by periodontitis: a questionnaire-based cross-sectional pilot study. Journal of peridontology. 2019;90:957–65. doi:10.1002/JPER.18-0578.

142. Lamprecht R, Struppek J, Heydecke G, Reissmann DR. Patients' criteria for choosing a dentist: comparison between a university-based setting and private dental practices. J Oral Rehabil. 2020;47:1023–30. doi:10.1111/joor.12995.

143. Rustemeyer J, Bremerich A. Patients' knowledge and expectations regarding dental implants: assessment by questionnaire. Int J Oral Maxillofac Surg. 2007;36:814–7. doi:10.1016/j.ijom.2007.05.003.

144. Wang Y, Bäumer D, Ozga A-K, Körner G, Bäumer A. Patient satisfaction and oral health-related quality of life 10 years after implant placement. BMC Oral Health. 2021;21:30. doi:10.1186/s12903-020-01381-3.

145. Adedapo HA, Lawal AO, Adisa AO, Adeyemi BF. Non-doctor consultations and self-medication practices in patients seen at a tertiary dental center in Ibadan. Indian J Dent Res. 2011;22:795–8. doi:10.4103/0970-9290.94671.

146. Ajayi DM, Arigbede AO. Barriers to oral health care utilization in Ibadan, South West Nigeria. Afr Health Sci. 2012;12:507–13. doi:10.4314/ahs.v12i4.17.

147. Ajayi DM, Gbadebo SO, Adebayo GE. Perception about tooth colour and appearance among patients seen in a tertiary hospital, South-West, Nigeria 2021. doi:10.11604/pamj.2021.38.38.21422.

148. Gbadebo OS, Lawal FB, Sulaiman AO, Ajayi DM. Dental implant as an option for tooth replacement: the awareness of patients at a tertiary hospital in a developing country. Contemporary Clinical Dentistry. 2014;5:302–6. doi:10.4103/0976-237X.137914.

149. Idowu EA, Afolabi AO, Fakuade BO, Akintububo OB, Ibiyemi O. Self-medication profile of dental patients attending a North Eastern tertiary hospital in Nigeria. Annals of Ibadan Postgraduate Medicine. 2019;17:173–80.

150. Lawal FB, Oladayo AAM. Sources and factors related to oral health-care information among dental patients of a teaching hospital in Ibadan, Nigeria. International Quarterly of Community Health Education. 2019;40:17–21. doi:10.1177/0272684x19833847.

151. Christell H, Gullberg J, Nilsson K, Heidari Olofsson S, Lindh C, Davidson T. Willingness to pay for osteoporosis risk assessment in primary dental care. Health economics review. 2019;9:14. doi:10.1186/s13561-019-0232-z.

152. Balenovic A, Fazlic A, Mihelcic M, Hoch A, Radujkovic V. Sociodemographic determinants and common reasons for visiting the emergency dental service in the City of Zagreb: Acta stomatologica Croatica. International Journal of Oral Sciences and Dental Medicine. 2019;53:247–54. doi:10.15644/asc53/3/6.

153. Sever I, Verbič M, Sever EK. Estimating willingness-to-pay for health care: a discrete choice experiment accounting for non-attendance to the cost attribute. J Eval Clin Pract. 2019;25:843–9. doi:10.1111/jep.13095.

154. Sever I, Verbič M, Klaric Sever E. Estimating attribute-specific willingness-to-pay values from a health care contingent valuation study: a best-worst choice approach. Applied Health Economics and Health Policy. 2020;18:97–107. doi:10.1007/s40258-019-00522-2.

155. Spalj S, Slaj M, Athanasiou AE, Govorko DK, Slaj M. The unmet orthodontic treatment need of adolescents and influencing factors for not seeking orthodontic therapy. Coll Antropol. 2014;38 Suppl 2:173–80.

156. Bahadori M, Ravangard R, Asghari B. Perceived barriers affecting access to preventive dental services: application of DEMATEL method. Iran Red Crescent Med J. 2013;15:655–62. doi:10.5812/ircmj.11810.

157. Moshkelgosha V, Golkari A. An evaluation of willingness to pay for orthodontic treatments in patients of Shiraz dental school clinic. Journal of Oral Health and Oral Epidemiology. 2013;2:35–43.

158. Moshkelgosha V, Mehrzadi M, Golkari A. The public attitude towards selecting dental health centers. J Dent Shiraz Univ Med Sci. 2014;15:129–34.

159. Moshkelgosha V, Salahi MA, Rostami S. Evaluation of perceived acceptability, beauty and value of different orthodontic brackets. Journal of Dental Biomaterials. 2015;2:33–8.

160. Saadatfar N, Jadidfard MP. Parents' preferences for preventive and curative dental services: a comparison between fissure sealant and composite filling using willingness-to-pay method. Int J Paediatr Dent. 2021;31:792–800. doi:10.1111/ipd.12778.

161. Armfield JM. What goes around comes around: revisiting the hypothesized vicious cycle of dental fear and avoidance. Community Dent Oral Epidemiol. 2013;41:279–87. doi:10.1111/cdoe.12005.

162. Beresford D, Klineberg I. A within-subject comparison of patient satisfaction and quality of life between a two-implant overdenture and a three-implant-supported fixed dental prosthesis in the mandible. Int J Oral Maxillofac Implants. 2018;33:1374–82. doi:10.11607/jomi.6666.

163. Luzzi L, Spencer AJ. Factors influencing the use of public dental services: an application of the theory of planned behaviour. BMC Health Serv Res. 2008;8:93. doi:10.1186/1472-6963-8-93.

164. Angelis P de, Manicone PF, Angelis S de, Grippaudo C, Gasparini G, Liguori MG, et al. Patient and operator centered outcomes in implant dentistry: comparison between fully digital and conventional workflow for single crown and three-unit fixed-bridge. Materials 2020. doi:10.3390/ma13122781.

165. Augusti D, Augusti G, Re D. Prosthetic restoration in the single-tooth gap: patient preferences and analysis of the WTP index. Clin Oral Implants Res. 2014;25:1257–64. doi:10.1111/clr.12264.

166. Re D, Ceci C, Cerutti F, Fabbro MD, Corbella S, Taschieri S. Natural tooth preservation versus extraction and implant placement: patient preferences and analysis of the willingness to pay. Br Dent J. 2017;222:467–71. doi:10.1038/sj.bdj.2017.271.

167. Jaafar A, Nasir WM, Ab Mumin, Nazirah, Elias NNA, Sabri MAM. Reasons for seeking dental care among adults at an academic dental centre and the associated factors. Archives of Orofacial Sciences. 2018;13:104–11.

168. Kohli S, Bhatia S, Kaur A, Rathakrishnan T. Public knowledge and acceptance of dental implant treatment in Malaysian population. J Interdiscip Dentistry. 2014;4:76. doi:10.4103/2229-5194.142938.

169. Tin-Oo MM, Saddki N, Hassan N. Factors influencing patient satisfaction with dental appearance and treatments they desire to improve aesthetics. BMC Oral Health. 2011;11:6. doi:10.1186/1472-6831-11-6.

170. Hansen Edwards C. An evaluation of the socioeconomic viability of a dental health care reform in Norway: a willingness to pay approach. Master thesis. Department of Health Management and Health Economics. The Faculty of Medicine. University of Oslo. 2013. https://core.ac.uk/download/pdf/30892531.pdf. Accessed 12 Sep 2023.

171. Nermo H, Willumsen T, Johnsen J-AK. Prevalence of dental anxiety and associations with oral health, psychological distress, avoidance and anticipated pain in adolescence: a cross-sectional study based on the Tromsø study, Fit Futures. Acta Odontol Scand. 2019;77:126–34. doi:10.1080/00016357.2018.1513558.

172. Trovik TA, Wisth PJ, Tornes K, Boe OE, Moen K. Patients' perceptions of improvements after bilateral sagittal split osteotomy advancement surgery: 10 to 14 years of follow-up. American journal of orthodontics and dentofacial orthopedics. 2012;141:204–12. doi:10.1016/j.ajodo.2011.06.039.

173. Vika M, Skaret E, Raadal M, Ost L-G, Kvale G. Fear of blood, injury, and injections, and its relationship to dental anxiety and probability of avoiding dental treatment among 18-year-olds in Norway. Int J Paediatr Dent. 2008;18:163–9. doi:10.1111/j.1365-263X.2007.00904.x.

174. Dudea D, Lasserre J-F, Alb C, Culic B, Ciutrila ISP, Colosi H. Patients' perspective on dental aesthetics in a South-eastern European community. J Dent. 2012;40:E72-E81. doi:10.1016/j.jdent.2012.01.016.

175. Tâncu AMC, Purcărea VL, Pantea M, Imre M. Key factors correlations in selecting dental services. Journal of Medicine and Life. 2019;12:83–9. doi:10.25122/jml-2019-0023.

176. Tudorici T, Feier R, Balcos C, Forna N. Socio-demographic factors and the partial edentulism in the adult population from Iasi, Romania. Romanian Journal of Oral Rehabilitation. 2017;9:68–72.

177. Ungureanu M-I, Mocean F. What do patients take into account when they choose their dentist? Implications for quality improvement. Patient Prefer Adherence. 2015;9:1715–20. doi:10.2147/PPA.S94310.

178. Gao X, Ding M, Xu M, Wu H, Zhang C, Wang X, et al. Utilization of dental services and associated factors among preschool children in China. BMC Oral Health. 2020;20:1–20. doi:10.1186/s12903-019-0996-x.

179. Leung KCM, McGrath CPJ. Willingness to pay for implant therapy: a study of patient preference. Clin Oral Implants Res. 2010;21:789–93. doi:10.1111/j.1600-0501.2009.01897.x.

180. Zhu J, Li J, Zhang Z, Li H, Cai L. Exploring determinants of health provider choice and heterogeneity in preference among outpatients in Beijing: a labelled discrete choice experiment. BMJ open 2019. doi:10.1136/bmjopen-2018-023363.

181. Shanahan D, O'Neill D. Barriers to dental attendance in older patients. Ir Med J. 2017;110:548.

182. Al-Batayneh OB, Al-Khateeb HO, Ibrahim WM, Khader YS. Parental knowledge and acceptance of different treatment options for primary teeth provided by dental practitioners. Frontiers in Public Health 2019. doi:10.3389/fpubh.2019.00322.

183. Vermaire JH, van Exel NJA, van Loveren C, Brouwer WBF. Putting your money where your mouth is: parents' valuation of good oral health of their children. Social Science & Medicine. 2012;75:2200–6. doi:10.1016/j.socscimed.2012.08.018.

184. Chebib N, Abou-Ayash S, Maniewicz S, Srinivasan M, Hill H, McKenna G, et al. Exploring older Swiss people's preferred dental services for when they become dependent. Research and science. 2020;130:876–84.

185. Meier A, Connert T, Dagassan-Berndt D, Filippi A. Dental trauma splint color preference of adults. Swiss Dent J. 2021;131:320–5.

186. Sendi P, Bertschinger N, Brand C, Marinello CP, Bucher HC, Bornstein MM. Measuring the monetary value of dental implants for denture retention: a willingness to pay approach. Open Dentistry Journal. 2017;11:498–502. doi:10.2174/1874210601711010498.

187. Tianviwat S, Chongsuvivatwong V, Birch S. Different dental care setting: does income matter? Health Econ. 2008;17:109–18. doi:10.1002/hec.1237.

188. Tianviwat S, Chongsuvivatwong V, Birch S. Optimizing the mix of basic dental services for Southern Thai schoolchildren based on resource consumption, service needs and parental preference. Community Dent Oral Epidemiol. 2009;37:372–80. doi:10.1111/j.1600-0528.2009.00481.x.

189. Hof M, Tepper G, Semo B, Arnhart C, Watzek G, Pommer B. Patients' perspectives on dental implant and bone graft surgery: questionnaire-based interview survey. Clin Oral Implants Res. 2014;25:42–5. doi:10.1111/clr.12061.

190. Pommer B, Zechner W, Watzak G, Ulm C, Watzek G, Tepper G. Progress and trends in patients' mindset on dental implants. I: level of information, sources of information and need for patient information. Clin Oral Implants Res. 2011;22:223–9. doi:10.1111/j.1600-0501.2010.02035.x.

191. van den Branden S, van den Broucke S, Leroy R, Declerck D, Hoppenbrouwers K. Measuring determinants of oral health behaviour in parents of preschool children. Community Dent Health. 2013;30:19–25. doi:10.1922/CDH_2897Branden07.

192. Atanasov N, Stoyanova R, Alexandrova M. Possibilities to model patients' preferences and their willingness to pay for a molar treatment. Dent Med Probl. 2016;53:41–9. doi:10.17219/dmp/60680.

193. Lalabonova CK. Impact of dental anxiety on the decision to have implant treatment. Folia Med (Plovdiv). 2015;57:116–21. doi:10.1515/folmed-2015-0029.

194. Fernandez RF, Bucchi C, Navarro P, Beltran V, Borie E. Bone grafts utilized in dentistry: an analysis of patients' preferences. BMC Med Ethics. 2015;16:71. doi:10.1186/s12910-015-0044-6.

195. Rojas-Torres J, Navarro-Caceres P, Fonseca GM. Attitudes, perceptions, and preferences of individuals from Temuco (Chile) about denture marking. J Forensic Sci. 2019;64:1187–95. doi:10.1111/1556-4029.13991.

196. Amjad F, Aziz S. Trends, awareness, and attitudes of patients towards replacement of missing teeth at University College of Dentistry. Pakistan Oral & Dental Journal. 2014;34:190–3.

197. Saleem F, Ashraf S, Javed F, Naveed M. Determinants of delay in presentation of patients with dental problems. Pakistan Journal of Medical and Health Sciences. 2018;12:1211–3.

198. Nair R, Yee R. Differences in willingness to pay for an extraction, a filling, and cleaning teeth at various levels of oral health-related quality of life, as measured by oral impacts on daily performance, among older adults in Singapore. Singapore Dent J. 2016;37:2–8. doi:10.1016/j.sdj.2016.10.003.

199. Bajrić E, Kobasglija S, Jurić H. Patients' reactions to local anaesthetic application devices in paediatric dentistry. Coll Antropol. 2015;39:685–90.

200. Widström E, Seppälä T. Willingness and ability to pay for unexpected dental expenses by Finnish adults. BMC Oral Health. 2012;12:35. doi:10.1186/1472-6831-12-35.

201. Chau, Man-ho, Jimmy. Community Health Project 2013/14. Dental care programme for Hong Kong secondary school students: parents’ choices and willingness to pay. 2014. https​://hdl. handl​e.net/10722​/20653​4. Accessed 12 Sep 2023.

202. Abdulwahab M, Al-Sayegh F, Boynes SG, Abdulwahab H, Zovko J, Close J. Assessing the need for anesthesia and sedation services in Kuwaiti dental practice. Anesth Prog. 2010;57:91–5. doi:10.2344/0003-3006-57.3.91.

203. Malak CA, Chakar C, Romanos A, Rachidi S. Appraisal of schoolchildren's feelings and attitudes towards dentists and their potential impact on oral health. J Contemp Dent Pract. 2021;22:23–6.

204. Dalanon J, Diano LM, Esguerra R, Belarmino MP, Docor MR, Rodis OM, Locsin R, Matsuka Y. The Cebuano mothers’ willingness to pay for dental healthcare. The Journal of the PDA. 2018;65:33–7.

205. Wedrychowska-Szulc B, Syrynska M. Patient and parent motivation for orthodontic treatment. A questionnaire study. Eur J Orthod. 2010;32:447–52. doi:10.1093/ejo/cjp131.

206. Tachalov VV, Orekhova LY, Kudryavtseva TV, Loboda ES, Pachkoriia MG, Berezkina IV, Golubnitschaja O. Making a complex dental care tailored to the person: population health in focus of predictive, preventive and personalised (3P) medical approach. EPMA Journal. 2021:1–12. doi:10.1007/s13167-021-00240-7.

207. Park S, Kim H-K, Choi M, Lee M. Factors affecting revisit intention for medical services at dental clinics. PLoS ONE. 2021;16:e0250546. doi:10.1371/journal.pone.0250546.

208. Awooda EM, Eltayeb AS, Hussein SA, Dayelnaiem SI, Abdelhamied MA, Abdulwahab Mohamed L, Taha SM. Knowledge, attitude and acceptance of dental implants among patients attending Khartoum dental teaching hospital. IOSR Journal of Dental and Medical Sciences. 2014;13:19–23. doi:10.9790/0853-131161923.

209. Nyamuryekung'e KK, Lahti SM, Tuominen RJ. Patients' willingness to pay for dental services in a population with limited restorative services. Community Dent Health. 2018;35:167–72. doi:10.1922/CDH_4227Nyamuryekunge06.

210. Bucchi C, Del Fabbro M, Arias A, Fuentes R, Mendes JM, Ordonneau M, et al. Multicenter study of patients' preferences and concerns regarding the origin of bone grafts utilized in dentistry. Patient Prefer Adherence. 2019;13:179–85. doi:10.2147/ppa.S186846.

211. Laothong W, Cheng H-C. Comparison of factors affecting orthodontic treatment motivation of Taiwanese and Thai patients in two hospitals. J Dent Sci. 2017;12:396–404. doi:10.1016/j.jds.2017.06.003.

212. Vernazza CR, Wildman JR, Steele JG, Whitworth JM, Walls AWG, Perry R, et al. Factors affecting patient valuations of caries prevention: using and validating the willingness to pay method. J Dent. 2015;43:981–8. doi:10.1016/j.jdent.2015.05.009.

213. Walshaw EG, Adam, Naeem, I, Palmeiro ML, Neves M, Vernazza CR. Patients' and parents' valuation of Fluoride. Oral Health Prev Dent. 2019;17:211–8. doi:10.3290/j.ohpd.a42666.

214. Nalbandian S, Millar BJ. The effect of veneers on cosmetic improvement. Br Dent J 2009. doi:10.1038/sj.bdj.2009.609.

215. Re D, Fessi S, Augusti G, Augusti D. The patient’s side: willingness-to-pay index for computerized anesthesia. Italian journal of dental medicine. 2016;1:49–54.

216. Tilashalski KR, Gilbert GH, Boykin MJ, Litaker MS. Racial differences in treatment preferences: oral health as an example. J Eval Clin Pract. 2007;13:102–8. doi:10.1111/j.1365-2753.2006.00661.x.

217. Wall T, Nasseh K, Vujicic M. Most important barriers to dental care are financial, not supply related. 2014. https://silo.tips/download/research-brief-most-important-barriers-to-dental-care-are-financial-not-supply-r. Accessed 12 Sep 2023.

218. Aarabi G, Valdez R, Spinler K, Walther C, Seedorf U, Heydecke G, et al. Determinants of postponed dental visits due to costs: evidence from the Survey of Health, Ageing, and Retirement in Germany. Int J Environ Res Public Health 2019. doi:10.3390/ijerph16183344.

219. Narby B, Kronström M, Söderfeldt B, Palmqvist S. Changes in attitudes toward desire for implant treatment: a longitudinal study of a middle-aged and older Swedish population. Int J Prosthodont. 2008;21:481–5.

220. Clarkson JE, Pitts NB, Goulao B, Boyers D, Ramsay CR, Floate R, et al. Risk-based, 6-monthly and 24-monthly dental check-ups for adults: the INTERVAL three-arm RCT: National Institute for Health Research. Health technology assessment 2020. doi:10.3310/hta24600.

221. Harris R, Vernazza C, Laverty L, Lowers V, Burnside G, Brown S, et al. Presenting patients with information on their oral health risk: the PREFER three-arm RCT and ethnography. Health Services and Delivery Research 2020. doi:10.3310/hsdr08030.

222. Nayee S, Kutty S, Akintola D. Patient attendance at a UK dental hospital emergency clinic. Br Dent J. 2015;219:485–8. doi:10.1038/sj.bdj.2015.883.

223. Paisi M, Baines R, Worle C, Withers L, Witton R. Evaluation of a community dental clinic providing care to people experiencing homelessness: a mixed methods approach. Health Expectations. 2020;23:1289–99. doi:10.1111/hex.13111.

224. El-Din MMN, Al-Shakhs FN, Al-Oudah SS. Missed appointments at a university hospital in Eastern Saudi Arabia: magnitude and association factors. J Egypt Public Health Assoc. 2008;83:415–33.

225. Milner MN, Anania EC, Candelaria-Oquendo K, Rice S, Winter SR, Ragbir NK. Patient perceptions of new robotic technologies in clinical restorative dentistry. Journal of Medical Systems. 2019;44:33. doi:10.1007/s10916-019-1488-x.

226. Papautsky EL, Rice DR, Ghoneima H, McKowen ALW, Anderson N, Wootton AR, Veldhuis C. Characterizing health care delays and interruptions in the United States during the COVID-19 pandemic: internet-based, cross-sectional survey study. J Med Internet Res. 2021;23:e25446. doi:10.2196/25446.

227. Da Kfouri MG, Moysés ST, Gabardo MCL, Nascimento AC, Da Rosa SV, Moysés SJ. The feminization of dentistry and the perceptions of public service users about gender issues in oral health. Scientific Electronic Library Online Brazil. Free themes. 2019;24:4285–96. doi:10.1590/1413-812320182411.00832018.

228. Maciel R, Salvador D, Azoubel K, Redivivo R, Maciel C, da Franca C, et al. The opinion of children and their parents about four different types of dental restorations in a public health service in Brazil. European archives of paediatric dentistry. 2017;18:25–9. doi:10.1007/s40368-016-0262-8.

229. Azarpazhooh A, Dao T, Ungar WJ, Da Costa J, Figueiredo R, Krahn M, Friedman S. Patients' values related to treatment options for teeth with apical periodontitis. J Endod. 2016;42:365–70. doi:10.1016/j.joen.2015.11.022.

230. Schwendicke F, Mostajaboldave R, Otto I, Doerferd CE, Burkert S. Patients' preferences for selective versus complete excavation: a mixed-methods study. J Dent. 2016;46:47–53. doi:10.1016/j.jdent.2016.01.006.

231. Sever I, Verbič M, Sever EK. Valuing the delivery of dental care: Heterogeneity in patients' preferences and willingness-to-pay for dental care attributes. J Dent. 2018;69:93–101. doi:10.1016/j.jdent.2017.12.005.

232. Sonneveld RE, Brands WG, Bronkhorst EM, Welie JVM, Truin G-J. Patients' priorities in assessing organisational aspects of a general dental practice. Int Dent J. 2013;63:30–8. doi:10.1111/idj.12001.

233. Jaapar M, Musa G, Moghavvemi S, Saub R. Dental tourism: examining tourist profiles, motivation and satisfaction. Tourism Management. 2017;61:538–52. doi:10.1016/j.tourman.2017.02.023.
