# Supplementary material for: Decision-making regarding dental treatments – What factors matter from patients’ perspective? A systematic review
Source: BMC Oral Health. 2025 Nov 25;26:289. doi: 10.1186/s12903-025-07032-9 (PMC12903421; doi:10.1186/s12903-025-07032-9)
Supplement: Supplementary file 1 — Additional file 1: A1. Guideline on literature search, selection, and analysis. A2. Search strategy. A3. PRISMA checklist. A4. SWiM checklist. A5. Search strings for databases, including hits. A6. Characteristics, factors of choice, and references of included articles (N = 233), sorted by number of identified articles per country (descending) within study designs I–V. A7. Methodological characteristics of included articles (N = 233), and search details. A8. Coding scheme, codebook, and framework, including definitions of excluded and summarized codes. A9. Code definitions. A10. Calculation of ICA and ICR. A11. Quality assessment by MMAT: study design I. A12. Quality assessment by MMAT: study design II. A13. Quality assessment by MMAT: study design III. A14. Quality assessment by MMAT: study design IV. A15. Quality assessment by MMAT: study design V. A16. MMAT assessment results description. [file 12903_2025_7032_MOESM1_ESM.zip › A7_Methodological_characteristics_and_search_details.docx]

**A7.** Methodological characteristics of included articles (N = 233), and search details

| **No.** | **Reference^1^: author (year)** | **Data collection instrument^2^** | **Methodology  answering^2^** | **Recruitment strategy** | **In-/exclusion criteria** | **Incentive** | **Duration** | **Search details** | |
| --- | --- | --- | --- | --- | --- | --- | --- | --- | --- |
|  |  |  |  |  |  |  |  | **Time-point** | **Source** |
| **I Qualitative studies** | | | | | | | | | |
| I.1 | Al-Moghrabi et al. (2019)^3^ | in-depth interviews | semi-structured | purposive sampling, recruited from a previous RCT | inclusion: patients wearing vacuum-formed retainers for 4 years, reflecting a variety of adherence levels | ns | ns | update | DB |
| I.2 | Borreani et al. (2010) | in-depth interviews, focus group interviews | semi-structured | recruited through patient institutions (e.g., day centres), contacted by telephone/e-mail to invite clients | inclusion: older people and carers of older people | ns | ns | update | SR |
| I.3 | Ellis et al. (2011)^3^ | focus group interviews | semi-structured | recruited from list of people (after advertisement) who wished for dentures replacement, but declined an implant; recruited from waiting list of patients who had been offered an implant solution and refused it | inclusion: ≥50 years of age; complete denture-wearers seeking replacement; refusal to receive implants to support new dentures | ns | ns | update | SR |
| I.4 | Exley et al. (2012) | in-depth interviews | open | asked by dentists via sending out information; interested participants returned a "consent to contact" form | inclusion: patients who considered paying for dental implant treaments in primary care; informed consent | ns | ns | initial | DB |
| I.5 | Grey et al. (2013)^3^ | in-depth interviews (telephone) | semi-structured | adults who consulted specialist, w/ interest in implantology at private dental practice in focused region | inclusion: adults who had consulted a restorative dental specialist with an interest in implantology at a private dental practice in the focused region; fluent in English | ns | 26-53min | update | SR |
| I.6 | Hanefeld et al. (2015) | (1) in-depth interviews, (2) focus group interviews | open | call posted on research project's website, online/information forums, experts asked by researchers | inclusion: informed consent | ns | (1) 30-100 min, (2) several hours | initial | DB |
| I.7 | Kashbour et al. (2018)^3^ | in-depth interviews (face-to-face, telephone) | semi-structured | recruited from pool of patients with current/previous treatment at focused institution | inclusion: ≥18 years of age; either gender; partially dentate/edentulous; at any stage of implant treatment pathway; able to communicate in English | ns | 20-42 min | update | DB |
| I.8 | Ke et al. (2013) | in-depth interviews (telephone) | semi-structured | invitation to participate given or sent, and provided with an information sheet and consent form to return to the researcher | inclusion: parents/guardians of children born with CLP | ns | ns | update | SR |
| I.9 | Scott et al. (2009) | in-depth interviews | open | newly referred patients to the focussed institutions were invited to participate | inclusion: ≥18 years of age; English speaking, with potentially malignant oral mucosal symptoms  (e.g., persistent oral pain, swelling in oral cavity);  completed questionnaire; informed consent | ns | 15-30 min | initial | SR |
| I.10 | Serban et al. (2019)^3^ | in-depth interviews | semi-structured | ns | inclusion: patients with rheumatoide arthritis, fulfilling ACR classification criteria; informed consent | financial incentive | ns | update | DB |
| I.11 | Thompson et al. (2020) | ethnographic observations, FU interviews | semi-structured | recruited by trained practice management teams at each research site | inclusion: ≥18 years of age; attending for NHS urgent dental care to address “[…] pain […] or […] infection”; consent for observation/audio record of appointment; informed consent; exclusion: pain or distress preventing ability to consent; attending for routine NHS or private dental care; accompanied by person <18 years of age/(ability to) consent; no translation service available,  if needed | ns | ns | update | DB |
| I.12 | van der Zande et al. (2021) | interviews and FU interviews | semi-structured | purposive sampling | inclusion: adults without a dentist seeking care from urgent dental services; diverse demographic characteristics, prioritizing patients with longer expected waiting times; written consent | ns | <5-≥20 min | update | DB |
| I.13 | Bohn et al. (2018) | in-depth interviews | semi-structured | recruited in waiting rooms at clinic | inclusion: ≥18 years of age; fluent in English; informed consent | $10 gift card | ns | update | DB |
| I.14 | Cohen et al. (2007) | focus group interviews | open | recruited from former project or via message boards/ screening at institutions | inclusion: >20 years of age; low-income non-Hispanic, White, Black, Hispanic, with toothache in previous 12 months; self-care, or care ≥1 times from a non-dentist; informed consent | $40 (participation expenses) | avg. 2 hours | initial | SR |
| I.15 | Dodd et al. (2014) | in-depth interviews | semi-structured | recruited during meetings sponsored by community institutions, incl. oral study description | inclusion: 10-18 years of age, resident in medically underserved and dental health professional shortage region | $35 gift card | 45-60 min | update | SR |
| I.16 | Gatten et al. (2011) | focus group interviews | open | randomly selected from database of focused departments, invited by telephoned | inclusion: ≥18 years of age; with root canal therapy or implant-based rehabilitation, coronal restoration with ≥1 year in occlusal function; treatment by clinicians with same level of proficiency (i.e., graduate students); ns; informed consent | ns | 90 min | initial | DB |
| I.17 | Hoeft et al. (2011) | in-depth interviews | open | recruited by bilingual staff | inclusion: primary caregivers of children ≤10 years of age, at least 1 child ≤5 years of age; self-identified as first- or second-generation immigrants from Mexico; informed consent | $20 gift card (grocery store) | ns | initial | SR |
| I.18 | Horton et al. (2009) | in-depth interviews | open | recruited by bilingual staff | inclusion: primary caregiver of children < 6 years of age; of Latino origin; informed consent | $20/$10 gift card (local stores); 1st/further interviews | 1-2 hours | initial | SR |
| I.19 | Siegel et al. (2012) | in-depth interviews | open | street-intercept method was used in which pedestrians were approached and requested | inclusion: ≥18 years of age; african American men and women living in focused neighborhood; (parents) born in the country; to self-identify as non-Hispanic African American; lived in focused neighborhood ≥5 years; experienced ≥1 oral symptom during previous 6 months lasting ≥2 days; informed consent | $2 gift card (fastfood restaurant), $50 cash, further (transportation costs) | 90-120 min | initial | SR |
| I.20 | Brown et al. (2020) | online search (first pages in search engine) | ns | cold aquised using a search engine | inclusion: google search; first-person narrative written by individual or partner with treatment on trip, English, with description of procedure type, with personal experience in country, publicly available; exclusion: promotional purposes, narrative not written by patient or partner, written by doctors; photo/video blogs; duplicate | ns | ns | update | DB |
| I.21 | Nogueria et al. (2019)^3^ | focus group interviews | semi-structured | recruited from ongoing clinical studies at focused dental institution | inclusion: fully edentulous for at least 1 year | no financial incentive (possibly: dentures) | 60-90 min | update | SR |
| I.22 | Mostajer Haqiqi et al. (2016) | in-depth interviews | semi-structured | recruited by dentistry division of hospital; nurses and staff invited interested parents in hospital ED | inclusion: <10 years of age; seeking care for nontraumatic dental problems in the ED | ns | 50-80 min | update | SR |
| I.23 | Atieh et al. (2016) | in-depth interviews | semi-structured | sample from original clinical trial | inclusion: 3-4 remaining osseous walls at time of implant placement; ≥2mm of tissue; oral hygiene; bone quality; compliance and commitment for FU | oral implant therapy at reduced costs | ns | update | SR |
| I.24 | Giddings et al. (2008) | in-depth interviews | semi-structured | purposive sampling, snowball technique | inclusion: ≥65 years of age; English speaking; patients who could comprehend the study details | ns | 45-90 min | update | SR |
| I.25 | Gregory et al. (2012) | in-depth interviews | ns | randomly selected from focused region electoral rolls | inclusion: older people in focussed regions selcted from regions' electoral rolls | ns | ns | update | SR |
| I.26 | McKenzie-Green et al. (2009) | in-depth interviews | semi-structured | purposive sampling using snowball technique | ns | ns | 45-90 min | update | SR |
| I.27 | Osman et al. (2014) | in-depth interviews | open | recruited from patients attending university clinic or referrals from dentists and denturists, randomly assigned to one treatment group | inclusion: be fully edentulous, medically, and psychologically suitable for implant surgery; with sufficient alveolar bone for implants, understand English or accompanied by responsible adult; informed consent | ns | ns | initial | DB |
| I.28 | Sussex et al. (2010) | in-depth interviews | semi-structured | purposive maximum-variation sampling | inclusion: ≥75 years of age; to be currently resident in the focussed region; and to have lost (or had extracted) all their teeth some time prior to 1960 while living in New Zealand | ns | ns | initial | DB |
| I.29 | Abrahamsson et al. (2017) | in-depth interviews | open | patients referred to clinic | inclusion: patients with treatment of peri-implantitis | ns | 25-65 min | update | SR |
| I.30 | Johannsen et al. (2012)^3^ | in-depth interviews | open | selected from two clinics databases | inclusion: with dental implant treatment at the focussed institutions | ns | ns | update | SR |
| I.31 | Narby et al. (2012) | in-depth interviews | open | strategicalley selected from the register at the clinic | inclusion: selected from clinic register on basis of gender, age, and place of residence; informed consent | ns | up to 1 hour | initial | DB |
| I.32 | Ostberg et al. (2013) | in-depth interviews | open | purposive sampling from data record system of public dental service, related to gender and choice of payment system (e.g., capitation plan) | inclusion: with full dental examination in public dental service during the past 3 years; range in age (young/old adult) and residence (rural/urban); informed consent | ns | 25-45 min | initial | DB |
| I.33 | Canuto et al. (2018) | in-depth interviews | semi-structured | invited until data saturation was reached | inclusion: ≥18 years of age; male; descended from indigenous people | $50 gift card | approx. 17 min | update | DB |
| I.34 | Slack-Smith et al. (2010) | focus group interviews, in-depth interviews | semi-structured | purposive sampling | inclusion: participants representing a range of sociodemographic and organisational groups | ns | ns | update | SR |
| I.35 | Azhar et al. (2018) | in-depth interviews | semi-structured | purposive sampling | inclusion: diagnosed oral cancer patients, aged 18 years old and above with disease staging from TNM stage III-IV | Malaysian Ringgit 30 | ns | update | DB |
| I.36 | Cronin et al. (2009) | in-depth interviews | open | random sample from focussed region via national database by letter of invitation | inclusion: experience with fixed and removable prostheses; informed consent | €30 gift card | ns | initial | DB |
| I.37 | Niesten et al. (2013) | in-depth interviews | open | random selection of regular daycare centers and assisted-living homes in the focussed region from a national register | inclusion: informed consent; exclusion: patients with health status beyond "frail" according to ZZP-score | ns | ns | initial | DB |
| I.38 | Vanobbergen (2007) | focus group interviews | open | recruited in social services, local neighborhood, and mother’s groups | inclusion: verbal consent; exclusion: previously lived in poverty but now employed as social workers in deprived regions | ns | 1,5 hours | initial | DB |
| I.39 | Mittal et al. (2019) | in-depth interviews | semi-structured | purposive sampling, recruited while waiting to see dentist/medical practitioner | inclusion: ≥65 years of age; eligible for subsidised dental care plans; able to understand English, Chinese, or Malay | ns | 15-30 min | update | DB |
| I.40 | Munira Hernandez-Santos et al. (2021)^3^ | in-depth interviews | semi-structured | convenience sampling | inclusion: adults of older age; attending for dental attention at the dental school-hospital; different oral conditions of participants; agreeing to be part of the study | ns | ns | update | DB |
| I.41 | Naidu (2012) | focus group interviews | open | invitation-letters sent to parents by head-teachers of 3 preschools (from previous study in focussed region) | inclusion: parents and caregivers of children attending preschools in focussed region; informed consent | ns | 45-50 min | initial | SR |
| I.42 | Abd Mutalib et al. (2017) | online search (blog posts, forums) | open | ns | exclusion: studies non-English; not meeting research questions requirements; not publicly available, from non-promotional/ facilitators' website, of length more than a sentence, of non-expatriate; dublicate | ns | 9 months | initial | DB |
| **II Quantitative randomized controlled trials - RCTs** | | | | | | | | | |
| II.1 | Harris et al. (2020) | self-administered, dentist-supported survey; WTP: self-administered questionnaire; FU by telephone/e-mail | open, closed; WTP (shuffled card) | patients invited when making appointment for NHS dental check-up | inclusion: adults (>18 years of age) with at least some teeth, identified as Red or Amber risk for poor oral health using the TL algorithm being piloted elsewhere in NHS practices; exclusion: patients identified as ‘Green’ (low risk); patients attending for an emergency appointment (because a full check-up and risk assessment is not usual care); and patients with low English language ability who required an interpreter for appointments | ns | after 12 months (FU) | update | DB |
| II.2 | Bender et al. (2007) | self-administered questionnaires | closed | random sample of patients asked in clinic reception region | exclusion: indian/native patients; who identified their background as bi- or multiracial; no report of race or gender, no ability to communicate in English; grant consent | none | March to August 03-08/2005 | initial | DB |
| II.3 | Andrade et al. (2013) | interviewer-administered surveys (telephone) | closed | patients asked upon arrival at the clinic with freedom to decline | inclusion: pre-diagnosed with periodontal disease; informed consent | ns | ns | initial | DB |
| II.4 | Esfandiari et al. (2009) | interviewer-administered questionnaires | open, closed; WTP (bidding game, unclear/ open, Yes/No), WTA (Yes/No) | invitation for participants from another study | inclusion: > 65 years of age; French speaking residents; completely edentulous | ns | avg. 20 min | initial | DB |
| II.5 | Heydecke et al. (2008) | self-administered questionnaires | closed | invitation of patients visiting the focussed clinic | inclusion: 50-85 years of age; fully edentulous, requiring a new set of dentures, able to read, understand German language; informed consent; exclusion: symptoms of defined dental disorders (e.g., xerostomia); psychologic or psychiatric conditions that could influence response to treatment | ns | January 2003 and April 2005 | initial | SR |
| II.6 | Felice et al. (2009) | interviewer-administered surveys (telephone) | open, closed | recruited in different private practices and two hospitals | inclusion: ≥18 years of age; edentulous patient having bilateral edentulism in posterior jaws (premolars and molars) with a similar degree of bone resorption requiring one to three implants 5 mm long; informed consent | ns | Juli 2008 to September 2009 | initial | DB |
| II.7 | McKenna et al. (2016)^3^ | interviewer-administered questionnaires | open; WTP (payment cards) | all included patients had been provided with oral rehabilitation 24 months previously as part of another study comparing 2 tooth replacement strategies | inclusion: ≥65 years of age; partially dentate; minimum of 6 remaining natural teeth in one arch, no systemic medical conditions preventing routine dental treatment, no evidence of dementia, were able to have dental treatment in a dental chair; English language | ns | ns | initial | DB |
| II.8 | Albonni et al. (2021) | investigator-administered questionnaires | closed, scale | randomization (tossing a coin) | inclusion: in good health; 18-60 years of age; both genders; with minimum of 20 teeth; not smokers and non-alcoholic; not pregnant or breastfeeding; no severe oral habits; other clinical values; informed consent; exclusion: bruxism; defined medical conditions, e.g., major systemic illnesses (e.g., diabetes, cancer), limitations precluding oral hygiene | ns | 6 weeks | update | DB |

| **III Quantitative non-randomized controlled trials – Non-RCTs** | | | | | | | | | |
| --- | --- | --- | --- | --- | --- | --- | --- | --- | --- |
| III.1 | Al Garni et al. (2012) | interviewer-administered questionnaires | closed; WTP (bidding game) | randomly selected patients reporting to the focussed institutions | inclusion: with ≥1 missing teeth; uninsured; informed consent | ns | September 2011 to January 2012 | initial | DB |
| III.2 | Atchison et al. (2007) | interviewer-administered questionnaires | open, closed; WTP (ns) | sample of patients, who were informed about the study and its purpose and were given an invitation-letter to participate | inclusion: English or Spanish language; patients having either a mandibular fracture or planning to undergo surgical removal of impacted third molar teeth under general anesthesia; informed consent | ns | ns | initial | SR |
| III.3 | Eyuboglu et al. (2020) | scale (VAS) questionnaires | closed, scale, clinical data | ns | inclusion: <18 or >60 years of age; patients who had symptomatic teeth requiring non-surgical root canal treatment; exclusion: patients diagnosed with systemic diseases, and patients who had used an analgesic up to 12 hours prior to the appointment | ns | ns | update | DB |
| III.4 | Yuzbasioglu et al. (2014) | self-administered questionnaires | closed | first year students of focussed institution; recruitment after examination of criteria | inclusion: no experience with conventional or digital impressions; good general health/oral hygiene/mental health; no periodontal disease; informed consent; exclusion: previous impression experience; fixed or removable prosthetic rehabilitation; orthodontic treatment and preventive appliances; use of space maintainers; moderate to excessive dental anxiety | ns | ns | initial | DB |
| III.5 | Koberlein et al. (2011) | interviewer-administered questionnaires | open, closed; WTP (bidding game) (unclear/open, Yes/No), WTA (Yes/No) | part of an omnibus survey | inclusion: 30-75 years of age | ns | ns | initial | DB |
| III.6 | Al-Dwairi et al. (2014) | interviewer-administered questionnaires | ns | patients attending the focussed removable prosthodontic clinic | inclusion: with construction of new complete and removable partial dentures | ns | ns | initial | SR |
| III.7 | Al-Quran et al. (2011) | self-administered questionnaires | closed | ns | inclusion: ≥18 years of age; successful treatment ≥1 year before study; informed consent; exclusion: any complications with prosthesis (e.g., inflammation), repaired cases, and crown fractures; ≥2 adjacent missing teeth, with edentulous spaces in the 3rd molar region; with (mental) special needs | ns | ns | initial | DB |
| III.8 | Fragouli et al. (2016) | self-administered questionnaires | closed | questionnaire distributed to patients of focussed clinic | inclusion: patients receiving root canal treatment | ns | ns | initial | DB |
| III.9 | Re et al. (2018)^3^ | questionnaires | open, closed; WTP (bidding game), scale | consecutive patients attending a private dental clinic | inclusion: patients presenting with two distinguished carious cavities of same class (I or II) in upper jaw, treatment in 2 consecutive sessions not >1 week apart; exclusion: with systemic diseases in/directly afflicting the neurological condition or altering pain perception | ns | ns | initial | DB |

| **IV Quantitative descriptive studies** | | | | | | | | | |
| --- | --- | --- | --- | --- | --- | --- | --- | --- | --- |
| *a. Cross-sectional studies* | | | | | | | | | |
| IVa.1 | Austin et al. (2009) | self-administered questionnaires | open, closed | questionnaire administered to all adults seeking dental care | inclusion: ≥18 years of age; patients that saw a dentist during their visit; and consented to participate; exclusion: violent/aggressive/abusive attendees | ns | March to October 2006 (two time periods) | initial | DB |
| IVa.2 | Fenton et al. (2021) | self-administered questionnaires | closed; DCE, WTP (ns) | sample was recruited from 4 NHS general dental practices | inclusion: ≥18 years of age; could read/write in English; capacity to consent for participation | ns | January 2018 and February 2018 | update | DB |
| IVa.3 | Furnham et al. (2009) | self-administered questionnaires (online) | closed | conducted by an online marketing research company, opted to take part in online surveys, and were recruited from a variety of different sources (e.g., email, web sources, advertising) | inclusion: representative sample of population in terms of age, sex, socioeconomic class, and educational qualifications | ns | ns | initial | DB |
| IVa.4 | Geoghegan et al. (2019) | self-administered questionnaires | closed | questionnaire distributed to new patients (and parents) referred for orthodontic assessment at 3 orthodontic specialist practices in focussed regions | inclusion: <18 years of age; registered with general dentists within the respective NHS primary care trust to the specialist orthodontic practice they attended; all patients were referred from their dentist for an orthodontic assessment by a specialist and had not been previously assessed | ns | ns | update | DB |
| IVa.5 | Goodwin et al. (2011) | interviewer-administered questionnaires (telephone) | closed | random sampling of household and participant within household; potential participants called | inclusion: ≥16 years of age; representative of the adult population; landline telephone | ns | 3 weeks | initial | DB |
| IVa.6 | Hill et al. (2013) | survey; retrospective | closed, scale | analysis of survey data from previous study | ns | ns | ns | update | SR |
| IVa.7 | Marshman et al. (2012)^3^ | self-reported questionnaires (postal) | closed, scale | questionnaires sent to 25,200 adults, representatives of region, via patients database of general practitioners | inclusion: ≥16 years of age | ns | 2008 | update | SR |
| IVa.8 | Swami et al. (2011) | self-administered questionnaires | closed | the authors directly recruited participants from several university locations, including cafeterias, libraries, and lecture theatres | inclusion: participation had been agreed | ns | ns | initial | DB |
| IVa.9 | Vernazza et al. (2015a) | interviewer-administered questionnaires | closed; WTP (payment cards) | patients from dental practices in focussed region; purposive sampling of practices by size, location, and patient payment type (public, private, mixed) | inclusion: ≥18 years of age; routine dental attenders from dental practices in focussed region; patients having at least one natural tooth; exclusion: participant could not understand or complete the interview | ns | 51 days | initial | DB |
| IVa.10 | Al Shaman et al. (2019)^3^ | self-administered questionnaires (web-based) | closed | coin toss method to select participants from all patients of the focussed clinic | inclusion: >18 years of age; patients with missing one or more teeth; participants willing to answer all survey questions | ns | ns | update | DB |
| IVa.11 | Aldaij et al. (2018)^3^ | self-administered questionnaires (tablet PC) | structured, closed | patients who attended outpatient department of university dental clinics of Munasiya campus of Riyadh Elm University | ns | ns | ns | update | SR |
| IVa.12 | Al-Hussyeen et al. (2010) | self-administered questionnaires | closed | random selection of schools | exclusion: did not visit the dentist during the last 2 years | ns | ns | initial | DB |
| IVa.13 | Ali et al. (2020) | self-administered questionnaires | ns | ns | ns | no financial incentive | ns | update | DB |
| IVa.14 | Al-Johany et al. (2010) | self-administered questionnaires | ns (ns, closed) | questionnaires were handed to patients during their regular dental visit | ns | ns | ns | initial | SR |
| IVa.15 | Alsarheed et al. (2011) | questionnaires | open, closed, scale | sample of children with a questionnaire developed by study investigators | inclusion: 9-12 years of age; attending public schools | ns | ns | update | SR |
| IVa.16 | Alshukairy et al. (2020) | self-administered questionnaires | structured, closed | parents presenting to the focussed institution for dental treatment, to determine their acceptance to treat their children under general anaesthesia for dental procedures | exclusion: parents with children older than 14-year-old and less than the age of 3 and having children with mental impairment and cardiac diseases or any other medical or mental problem | ns | 8-10 min | update | DB |
| IVa.17 | AlZarea et al. (2017) | self-administered questionnaires | closed | non-probability purposive sampling technique | inclusion: ≥60 years of age; geriatric patients who consented and belonged to the working age group, visiting department for routine check-up, scaling, with complaints of pain, for prosthesis | ns | ns | initial | DB |
| IVa.18 | Bagher et al. (2019)^3^ | interview questionnaires | closed, scale | ns | inclusion: parents of healthy children ≤12 years of age, attending pediatric dental clinics; agreed to participate | ns | 3 months | update | DB |
| IVa.19 | Bahammam et al. (2019) | questionnaires (self-reported) | closed | children visiting the focussed hospital for treatment at the pediatric department | inclusion: 9-11 years of age; have visited dentist previously; exclusion: siblings of child | ns | 4 months | update | DB |
| IVa.20 | Fatani et al. (2016) | interview‑based questionnaires | closed, open | convenience sample of patients | ns | ns | ns | update | SR |
| IVa.21 | Fawaz (2015) | self-administered questionnaires | ns | questionnaires handed to the patients during their regular dental visits | inclusion: >20 years of age; informed consent; exclusion: <20 years of age | ns | September 1st, 2014, to November 20th, 2014 | initial | SR |
| IVa.22 | Gaffar et al. (2014) | self-administered questionnaires | closed | non-probability consecutive sampling (time-bond) of waiting patients | inclusion: ≥18 years of age; all new hospital patients | ns | March 2012 to February 2013 | initial | DB |
| IVa.23 | Kakti et al. (2020) | questionnaires (sent via e-mail or messenger service) | ns | records of pediatric patients in focused clinic, parents contacted | inclusion: 0-12 years of age; healthy patients (ASA I); with completed comprehensive dental treatment and full mouth rehabilitation under general anesthesia; exclusion: >12 years of age; medically compromised or special health needs | ns | 2015 to 2020 | update | DB |
| IVa.24 | Madarati et al. (2018) | self-administered questionnaires | closed | patients were asked for voluntary participation after they underwent a session of a root canal treatment | ns | ns | January 2017 to June 2017 | initial | DB |
| IVa.25 | Mubaraki et al. (2017) | self-administered forms | closed; WTP (bidding game) | purposive sampling | inclusion: 5-9 years of age (parents); children with extraction of ≥1 primary tooth presenting to focused dental clinic | ns | ns | update | SR |
| IVa.26 | Quadri et al. (2018) | self-administered questionnaires | open, closed | patients visiting the dental clinics and centers in focussed region | inclusion: adults; could answer the questionnaire without assistance; exclusion: subjects who were physically or mentally challenged; did not give consent to participate | ns | January 2017 to February 2017 (incl. design) | initial | DB |
| IVa.27 | Sabbagh et al. (2020) | interviewer administered questionnaire (phone) | ns, closed, scale | all parents of children attending the pediatric dental clinic September 2017 to June 2018 | inclusion: parents attending clinic with healthy children, at least 1 appointment during period of study, speaks Arabic; exclusion: children receiving no treatment, treatment in other than focused clinic, medically compromised | ns | September 2018, 15 min | update | DB |
| IVa.28 | Shabbir et al. (2018)^3^ | self-administered questionnaires | open, closed | patients visiting the dental clinics were selected at random | inclusion: literate patients attending military hospitals in the focussed region; exclusion: patients who could not read or write | ns | ns | initial | DB |
| IVa.29 | Shahrani et al. (2015) | self-administered questionnaires | closed | all patients consulting the focussed clinics; questionnaires were distributed at registration by the receptionist | inclusion: all patients consulting the focussed clinics; willing to participate | ns | ns | initial | DB |
| IVa.30 | Taibah et al. (2018) | self-administered questionnaire | open, closed | questionnaires were distributed to patients in public and private clinics and hospitals; convenient sample of participants in focussed region | inclusion: >18 years of age | ns | ns | update | DB |
| IVa.31 | Chambers et al. (2019) | survey | ns | surveys packets mailed to 100 graduates of the focussed institution who had answered a previous e-mail inquiry and agreed to distribute survey to patients waiting in reception region in their practices | ns | ns | ns | update | DB |
| IVa.32 | Cohen et al. (2008) | interviewer-administered questionnaires (telephone) | ns | stratified random sample of households in region due to consensus data | inclusion: ≥21 years of age; non-Hispanic white, non-Hispanic black and Hispanic adults from focussed region, who had experienced a toothache during the previous 12-month period; low-income (annual family income of less than $25,000); informed consent | $10 gift card | ns | initial | DB |
| IVa.33 | Crystal et al. (2017)^3^ | survey (web-based) | closed, closed (score) | sample selescted from parents who were waiting for their children’s appointments at the clinics | inclusion: parents must have had children who had caries experience; speak English or Spanish; agreed to participate in the survey | no incentive | no | update | SR |
| IVa.34 | Flores et al. (2008) | interviews (telephone); retrospective | open, closed, scale | random-digit-dial sample, selection of households from all states and the capital city; random selection of max. 1 child in each household | inclusion: <18 years of age (children) | financial incentive | January 29th, 2003, to July 1st, 2004; 23-29min | update | SR |
| IVa.35 | Kelly et al. (2014) | computer-administered questionnaire | closed | all parents who brought their child for a treatment to the focussed hospital asked before attending appointment | inclusion: child's initial screening visit in the focussed clinic | ns | September 2012 to December 2012 | update | DB |
| IVa.36 | Kim et al. (2012) | self-administered questionnaires (mail) | closed | questionnaires mailed to patients’ addresses obtained from the insitution's patient registration file | inclusion: ≥18 years of age; patients who made 1st visit to the focussed practice; 1st visit was documented with defined exam codes of limited oral/comprehensive oral evaluation | ns | September 2010 to October 2010 | initial | DB |
| IVa.37 | Olson et al. (2020) | questionnaire (web-based) | closed, scale | survey delivered via a commercial polling company with existing databases of contact information for reliable panelists; electronic invitations | inclusion: 18-65 years of age (adults); residing in the United States | ns | ns | update | DB |
| IVa.38 | Samuels et al. (2015) | interviewer-administered surveys (telephone) | ns | convenience sample of patients in focussed insitution; selection of phone numbers from automated autodial system for appointment reminders | inclusion: English- or Spanish-speaking; missed appointment within 1 week | $10 gift card | July 1st, 2011, to June 30th, 2012 | initial | SR |
| IVa.39 | Vela et al. (2012) | self-administered questionnaires | closed | consecutive patients scheduled in focussed clinic | inclusion: ≥18 years of age; patients who were scheduled for evaluation and/or initial nonsurgical RCT appointments in the focussed clinic | $10 gift card | ns | initial | DB |
| IVa.40 | Asokan et al. (2016) | questionnaires | closed, scale | three different schools at Bhopal; random selection | inclusion: 9-12-years of age | ns | ns | update | SR |
| IVa.41 | Bhatia et al. (2018) | self-administered questionnaires | ns | questionnaires handed to parents in waiting region while children undergoing treatment | ns | ns | 6 months | initial | DB |
| IVa.42 | Garcha et al. (2014) | self-administered questionnaires | closed | convenience sample of participants selected from country's register of social class | inclusion: 35-45 years of age; participants equally distributed to five social classes | ns | ns | initial | SR |
| IVa.43 | Jayakaran et al. (2017)^3^ | self‑administered questionnaires | closed | children visiting focussed clinic | inclusion: 6–10 years of age (children); informed consent of parents | ns | ns | update | SR |
| IVa.44 | Kamavaram Ellore et al. (2015) | self-administered questionnaires (completed in the waiting area prior treatment) | closed | ns | exclusion: siblings of the children participants (to avoid attrition of the parents’ group) | ns | 2 months | update | SR |
| IVa.45 | Keerthana et al. (2020) | questionnaires (e.g., choice scenarios) | closed | sample of children | inclusion: 6– 12 years of age (children); attending the Department of Pedodontics in a private dental college; consent of parents/guardians | ns | ns | initial | SR |
| IVa.46 | Mahajan et al. (2021)^3^ | choice scenarios: shade selection guides; reason for choosing orally asked | closed, ns | ns | inclusion: >50 years of age; patients with complete denture treatment needs; willing to participate in the study; exclusion: patients with partial dentures, single complete dentures and having any serious vision problems or issues | ns | May 2016 to September 201 | update | DB |
| IVa.47 | Manickam et al. (2010) | questionnaires | structured, ns; WTP (ns) | random sample selection | inclusion: 18-50 years of age | ns | March 2010 | update | SR |
| IVa.48 | Paul et al. (2019) | self-administered questionnaires | closed | convenient sampling | inclusion: all patients reporting to the focussed institution from 1st February 2019 to 7th February 2019 | ns | ns | update | DB |
| IVa.49 | Poudyal et al. (2010) | self-administered questionnaires | ns | random sampling of houses in area's administration list | inclusion: ≥18 years of age; residing in the selected house | ns | ns | initial | SR |
| IVa.50 | Pragati (2010) | interviewer-administered questionnaires | closed | people were surveyed from the urban population residing in focussed region | ns | ns | ns | initial | SR |
| IVa.51 | Raj (2014) | self-administered questionnaires | closed | random selection; patients, accompanying people, and residents of focussed regions | inclusion: at least a high school educational level; exclusion: dentist, dental technicians, and assistants | ns | ns | initial | SR |
| IVa.52 | Ravikumar et al. (2016)^3^ | dentist-administered questionnaires (choice scenarios) | closed, scale | in dental set up: children were selected who visited focused department for 1st time; in school environment: personal interviews conducted by paediatric dentist, after receiving approval from school administrator and parents | inclusion: 6-11 years of age (children); written consent of parents; exclusion: children with systemic illness, the children who required emergency dental management; the children who were called for reviews | ns | 6 months | update | SR |
| IVa.53 | Saha et al. (2013) | self-administered questionnaires | ns | all the subjects coming to dental institution | inclusion: >20 years of age; patients coming to dental institution; willing to give informed consent | ns | ns | initial | SR |
| IVa.54 | Shah et al. (2014) | interviewer-administered questionnaires | ns | patients attending the focussed dental outpatient department | inclusion: >16 years of age; all subjects coming to dental college; willing to give informed consent | ns | 1 month in April 2014 | initial | SR |
| IVa.55 | Shanmugam et al. (2020) | data of patients; retrospective | ns | ns | inclusion: patients who reported anterior spacing; with treatment for midline diastema in focused dental institution | ns | June 1st, 2019, to March 31st, 2020 | update | DB |
| IVa.56 | Shrirao et al. (2016) | self-administered questionnaires | closed | all the patients reporting at the focussed department | inclusion: all patients in department; give informed consent | ns | December 2014 to March 2015 | initial | DB |
| IVa.57 | Suprakash (2013) | self-administered questionnaires | closed | all patients who attended the focussed departments | inclusion: >15 years of age; all the patients who attended the focussed departments | ns | 2 months | initial | SR |
| IVa.58 | Verma et al. (2012) | interviewer-administered questionnaires | ns | multistage sampling, 1st stage: localities in region, 2nd stage: divisions, 3rd stage: random selection of street; 4th stage: random selection of 1st and consequtive house/s; 1 respondent a house | inclusion: adult | ns | 8 months | initial | SR |
| IVa.59 | Feldens et al. (2015) | self-administered questionnaires | closed | ns | inclusion: 12-13 years of age (age at which the permanent dentition is established); informed consent and affirmative agreement | ns | ns | initial | DB |
| IVa.60 | Feu et al. (2012) | survey: VAS scale, WTP | closed; WTP (ns), scale | ns | inclusion: 17-63 years of age; had never undergone orthodontic treatment with brackets or aligners; not being a dentist or a dental student, at the University campus in the four days of data collection; informed consent | ns | 4 days | update | SR |
| IVa.61 | Leles et al. (2009) | interviewer-administered questionnaires | closed | consecutive sample of edentulous patients attending focussed department | ns | ns | October 2007 to June 2009 | initial | DB |
| IVa.62 | Leles et al. (2011) | interviewer-administered questionnaires | closed | convenience sample of consecutive patients with prosthodontic needs assigned for treatment at the focussed institution | inclusion: patients with at least 1 untreated partially edentulous arch; exclusion: fully dentate or edentulous | ns | 2 months | initial | SR |
| IVa.63 | Matsumoto et al. (2017) | interviewer-administered questionnaires | closed | multistage sampling | inclusion: ≥13 years of age; insitution user | ns | February 2011 to May 2011 | initial | DB |
| IVa.64 | Oliveira et al. (2013) | self-administered questionnaires | ns | patients who received orthodontic treatment in the focussed city, fullfilling inclusion criteria, were contacted by phone | inlusion: treatment initiated after reaching the age of 20 years; completion of orthodontic treatment; submitted to only one corrective orthodontic treatment; orthodontic treatment did not involve orthognathic surgical procedures | ns | ns | initial | SR |
| IVa.65 | Souza et al. (2013) | questionnaires | closed | patients were chosen randomly | inclusion: users of conventional dentures; required to have received the new prothesis at least 6 months prior to study | ns | ns | initial | DB |
| IVa.66 | Souza et al. (2016) | interviewer-administered questionnaires | closed | random assignment | inclusion: 18-25 years of age; with or in final stages of orthodontic treatment with fixed appliances; exclusion: survey incompleted or damaged; with <1 year of treatment, started before age of 18 | ns | January 2008 to December 2008 | initial | SR |
| IVa.67 | Vieira et al. (2015) | questionnaires | closed | convenience sample of subjects | inclusion: >18 years of age; with some degree of tooth loss; no current dental treatment; residents of two capital cities in the focussed region | ns | January 2012 to March 2013 | initial | DB |
| IVa.68 | Gurler et al. (2018) | self-administered questionnaires (completed in the waiting room) | closed | random selection, patients referred to department of focussed institution with initial treatment plan and need for oral surgical procedures | exclusion: <18 years of age; subjects who are illiterate, undergoing psychiatric/ psychological therapy and/or psychiatric medication; unwilling to participate in study | ns | 2015 to 2016 | update | DB |
| IVa.69 | Kuscu et al. (2009) | survey (choice scenarios, anxiety scale) | closed, scale | ns | ns | ns | 2007 | update | SR |
| IVa.70 | Ozdogan et al. (2019) | self-administered questionnaires | closed | ns | inclusion: patients who applied to the Hospital for implant treatment between May 18th to September 29th 2017 | ns | ns | update | DB |
| IVa.71 | Ozveren et al. (2021)^3^ | self-administered questionnaires | closed | ns | inclusion: children who applied to focused dental institution for their FDV between December 2019 and March 2020; or children who had not undergone any dental procedure from another dentist; exclusion: children who had any mental/physical disability | ns | ns | update | DB |
| IVa.72 | Patır Münevveroğlu et al. (2014) | questionnaires | ns, closed, clinical data | questionnaire was administered to children | inclusion: 6– 12 years of age (children); attending the Department of Pedodontics, Faculty for routine followup; informed consent of patients/guardians | ns | ns | update | SR |
| IVa.73 | Tengilimoglu et al. (2017) | questionnaires | closed | ns | inclusion: resident in focussed region | ns | ns | initial | DB |
| IVa.74 | Tuncer et al. (2015) | self-administered questionnaires | closed | ns | inclusion: patients or parents/caregiver for consultation, or to apply for treatment in focussed department | ns | January 2013 to April 2013 | initial | DB |
| IVa.75 | Uslu et al. (2007) | self-administered questionnaires (mailed) | closed | stamp and return envelope to patients of previous treatment | inclusion: with orthodontic treatment for correction of dento-facial anomaly; no surgical intervention; retention phase completed ≥5 years; informed consent; exclusion: any type of syndromes, cleft lip and palate, or mental retardation, with psychological treatment, severe medical problem possibly disrupting participation | ns | ns | initial | SR |
| IVa.76 | Yahyaoglu et al. (2018) | self-administered questionnaires (before treatment), and survey (few interviews, after treatment) | closed, scale, clinical data | all referred patients waiting in the waiting room prior to the clinic visit | inclusion: 6-12 years of age, who were referred to clinics of the Department of Pediatric Dentistry, Faculty of Dentistry, Karadeniz Technical University over a period of 3 months. | ns | ns | update | DB |
| IVa.77 | Azarpazhooh et al. (2015) | interviewer-administered questionnaires (telephone) | closed | stratified random sampling; telephone listing compiled by retailer containing approx. 10,000 numbers | inclusion: 18-64 years of age; household income <$35,000 per year; worked a minimum of part-time hours; no full-time student; family income below the MBM | ns | ns | initial | DB |
| IVa.78 | Balevi et al. (2007) | interviewer-administered questionnaires | open, closed; WTP (bidding game) (unclear/open, Yes/No), WTA (Yes/No) | sample of schoolteachers from focussed region, identified by snowballing method for potential participants | inclusion: profession as teacher (representing average middle income dental patient, often with access to private dental insurance; educated); informed consent | ns | ns | initial | DB |
| IVa.79 | Okuda et al. (2021)^3^ | survey (web-based/online platform) | ns, closed | survey link was shared on two social media channels | inclusion: ≥18 years of age; exclusion: anyone who works in the dental field | ns | ns | update | DB |
| IVa.80 | Srivastava et al. (2014) | questionnaires (web-based) or telephone survey | closed; WTP (payment scale) | randomly selected; sample by database provider, representative for each of all provinces | inclusion: ≥25 years of age; Canadian citizens/ residents; with annual household income >$10,000 [CAD] | $200 [CAD] lottery ticket | ns | update | DB |
| IVa.81 | Srivastava et al. (2020) | self-administered questionnaires (online) | closed; WTP (ns) | convenience sample of individuals | inclusion: ≥age of 25 years; able to understand English; provincial taxpayers; dentate or partially edentate | ns | ns | initial | SR |
| IVa.82 | Hirschfeld et al. (2019) | self-administered questionnaires | closed | ns | inclusion: >18 years of age; BPE ≥3 in ≥3 sextants; diagnosis of generalized chronic periodontitis by defined criteria, initial presence of plaque and/or calculus; exclusion: with extensive prosthetic restorations; gaps of >1 missing tooth; dysgnathia (e.g., cleft lip) | ns | February 2013 to June 2013 | update | DB |
| IVa.83 | Lamprecht et al. (2020) | questionnaires | closed, scale | non-randomised cross-sectional study, consecutive samples of dental patients and general population subjects were recruited | inclusion: ≥18 years of age; patients with sufficient command of the German language to understand and complete the questionnaire | no financial incentive | ns | update | DB |
| IVa.84 | Rustemeyer et al. (2007) | questionnaires | open, closed | questionnaires distributed to referred patients in centres and practices | inclusion: referred patients; exclusion: answers to special questions incomplete; already received dental implant/consultation; contraindications (e.g., metastatic tumours) | ns | 6 months | update | SR |
| IVa.85 | Wang et al. (2021) | self-administered questionnaires (3x, patients were asked to fulfill three questionnaires) | scale, clinical examination | all patients treated in private periodontal practice, asked to fulfill 3 questionnaires after complete clinical re-examination | inclusion: ≥18 years of age at re-examination; with treatment in private periodontal practice (≥1 implant); available medical image at implant placement (+ 3 months) and/or time of inserting prostheses, and attachment level; non-pregnant or breastfeeding; partially edentulous dentition; completed questionnaires | ns | ns | update | DB |
| IVa.86 | Adedapo et al. (2011) | self-administered questionnaires | ns | patients seen at the focussed hospital over a 4-months period | inclusion: ≥10 years of age; patients presenting over a 4-month period; consented to participate | ns | 4 months | initial | DB |
| IVa.87 | Ajayi et al. (2012) | self-administered questionnaires | closed | questionnaires were randomly distributed in the waiting hall | inclusion: ≥16 years of age; exclusion: incompletely filled questionnaires | ns | ns | initial | DB |
| IVa.88 | Ajayi et al. (2021)^3^ | self-administered questionnaires | ns | all patients visiting the focussed clinics | inclusion: all patients attending 2 outpatient clinics at the focused dental institutions; exclusion: grossly inadequately filled questionnaires | ns | ns | update | DB |
| IVa.89 | Gbadebo et al. (2014) | self-administered questionnaires | ns | randomly selected from the database of the respective departments; patients were telephoned using a detailed recruitment script and invited to participate | inclusion: ≥18 years of age; informed consent | ns | 6 months | initial | SR |
| IVa.90 | Idowu et al. (2019)^3^ | interviewer-administered questionnaires | closed | convenient sampling method | exclusion: <18 and >75 years of age | ns | ns | update | DB |
| IVa.91 | Lawal et al. (2019)^3^ | self-administered with assistance/ research assistant administered questionnaires | structured | ns | inclusion: ≥16 years of age; attending the hospital’s dental clinic for the first time; not in pain or any form of distress as at the time of the study; consented to participate; exclusion: unable to fill the questionnaire due to communication barrier | ns | ns | update | DB |
| IVa.92 | Christell et al. (2019)^3^ | questionnaires | open, closed; WTP (payment cards) | patients in clinics were consecutively invited to participate | inclusion: >50 years of age; female; could read and comprehend Swedish language | ns | May and August 2013 | update | DB |
| IVa.93 | Balenovic et al. (2019)^3^ | self-administered (1. part) and practitioner-administered (2. part/clinical data) questionnaires | closed | random sample of patients who were given a survey before the admission to the emergency room of dental care | ns | ns | ns | update | DB |
| IVa.94 | Sever et al. (2019) | self-administered questionnaires, but face-to-face interviews used in some cases | structured, closed; DCE, WTP (ns) | patients who attended the school clinic were surveyed | inclusion: patients who attended the school clinic | ns | March 2016 to January 2017 | update | DB |
| IVa.95 | Sever et al. (2020) | self-administered questionnaire (paper-based) | closed; DCE, WTP (ns) | patients who attended the school clinic, which offers dental care without any out-of-pocket costs | inclusion: patients who attended the school clinic, which offers dental care without any out-of-pocket costs | ns | March 2016 to January 2017 | update | DB |
| IVa.96 | Spalj et al. (2014) | questionnaires | closed | schools in different regions were randomly selected in a cluster sampling procedure; sample was considered representative in terms of socioeconomic status and access to care | inclusion: sample was considered representative in terms of socioeconomic status and access to care | ns | ns | initial | DB |
| IVa.97 | Bahadori et al. (2013) | self-administered questionnaires | closed | systematic random sampling method from all referred patients | inclusion: feeling need or prognoses of sticking dental floss between teeth, sensitivity of teeth (e.g., cold water), swollen gums; not referred to dental clinic before root canal treatment; informed consent | ns | ns | initial | DB |
| IVa.98 | Moshkelgosha et al. (2013) | interviewer-administered questionnaires | ns, open | purposive sampling | inclusion: 15-54 years of age; patients attending focused clinic in a period in 2010; understanding explanations; ≥8 years of formal education; eager agreed to participate | ns | 2010 | update | SR |
| IVa.99 | Moshkelgosha et al. (2014) | interview questionnaires | closed; WTP (payment scale) | purposive sampling | inclusion: patients attending focussed clinics, students of one focused institution | ns | ns | update | SR |
| IVa.100 | Moshkelgosha et al. (2015)^3^ | self-administered questionnaires | ns (closed, ns) | multistage random sampling; 8 schools in focussed region randomly selected via administrative insitution; children in schools randomly selected via school register | inclusion: parents of schoolchildren; only if happy to participate | ns | ns | initial | DB |
| IVa.101 | Saadatfar et al. (2021) | face-to-face interviews (by structured questionnaire) | open, closed; WTP (payment cards) | patients asked by researcher asked in waiting room, sample size calculation by EPV method | inclusion: 6-15 years of age (parents); attending an Iranian public children’s dental clinic; able to communicate in Persian | ns | May 2019 to December 2019 (6 months period), approx. 25-30 min | update | DB |
| IVa.102 | Armfield et al. (2013) | self-administered questionnaires (telephone) | closed | random sample of interviewees, invited to have a telephone-questionnaire posted to them | inclusion: ≥15 years of age; and participants who indicated that they had at least one natural tooth; provided verbal consent/assent | ns | ns | initial | SR |
| IVa.103 | Beresford et al. (2018)^3^ | self-administered scales | closed, scale | self-initiative: information about study to public by local radio station; interested people could apply | inclusion: ≥18 years of age; fluent in English; good physical health (ASA I/II), edentulous with a poor prognosis; adequate quality/volume of bone; informed consent; exclusion: (planned) pregnancy during study; bad physical health (ASA III-VI); substance abuse; smoking habit; severe bruxism; unrealistic expectations; other medical diseases, e.g., uncontrolled diabetes, psychologic problems | ns | 4 months | update | DB |
| IVa.104 | Luzzi et al. (2008) | self-administered questionnaires | closed | random sample of patients, participants were informed of the study at the time they contacted the clinic for dental care | inclusion: ≥18 years of age; be dentate with 6 or more natural teeth; being a holder of a government concession card entitling them to public dental care | ns | 3.5 years | initial | DB |
| IVa.105 | Angelis et al. (2020) | scale (VAS) questionnaires | closed, scale | selecting edentulous patients needing implant restorations | inclusion: ≥20 years of age; general health; no contraindications for implant treatment; not yet received implant restorations; good oral hygiene; FMPS and FMBS cut-off at 15%; exclusion: certain medical diseases, e.g., bruxism, systemic conditions, ASA IV | ns | ns | update | DB |
| IVa.106 | Augusti et al. (2014) | self-administered questionnaires | closed; WTP (bidding game) | consecutive distributed | inclusion: patients' first time at clinic; informed conset and approval | ns | ns | initial | SR |
| IVa.107 | Re et al. (2017) | interviewer-administered questionnaires | closed; WTP (bidding game) | consecutive patients attending a dental clinic | inclusion: adult age; patients attending a private dental clinic for 1st time, with normal comprehension, and verbal expression; informed consent and approval | ns | ns | initial | DB |
| IVa.108 | Jaafar et al. (2018)^3^ | reason for visit retrieved from clinical records; retrospective | ns | clinical records | inclusion: ≥18 years of age; patients attended focused clinic for 1st time from January 2015 to December 2016 | ns | ns | update | DB |
| IVa.109 | Kohli et al. (2014) | self-administered questionnaires (printed and online) | ns | random sampling, questionnaires handed to patients during dental visit | exclusion: no informed consent | ns | May 2012 to December 2012 | initial | SR |
| IVa.110 | Tin-Oo et al. (2011) | interviewer-administered questionnaires | closed | patients who attended focussed dental clinic | inclusion: >18 years of age; understand the Malay language; newly registered patients, who have not received any dental treatment within the previous six months; have no clear evidence of cognitive disturbance | ns | June 1st, 2009, to January 31st, 2010 | initial | DB |
| IVa.111 | Edwards (2013) | face-to-face and telephone interviews, questionnaires | open, closed; WTP (payment cards) | simplified region probability sampling, randomly selected telephone numbers of citizens between the age of 21 and 75 living in focused city | inclusion: >21 years of age; citizens of Oslo; exclusion: < 21 years of age | ns | ns | update | SR |
| IVa.112 | Nermo et al. (2019) | questionnaires; clinical dental examination data | closed, scale | all first-year upper-secondary school students in focused region were invited | inclusion: first-year upper-secondary school students in northern Norway; written consent; exclusion: >18 years of age | ns | 2010 to 2011 | update | DB |
| IVa.113 | Trovik et al. (2012) | self-administered questionnaires | closed | all patients with defined malocclusions treated in focussed department | inclusion: patients of previous treatment group; patients had combined 2 defined treatments; no need of major dental restorations (incl. crowns or bridges in frontal region) during FU; not syndromic or medically compromised; informed consent | ns | ns | initial | DB |
| IVa.114 | Vika et al. (2008) | self-administered questionnaires | closed | random and proportional cluster sample from population of focussed region | inclusion: 18 years of age (adolescents born in 1983); attending high schools in the focussed region | ns | ns | initial | SR |
| IVa.115 | Dudea et al. (2012) | self-administered questionnaires | closed | administered to patients who requested dental treatment in the practices | ns | ns | 2007 to 2008 | initial | DB |
| IVa.116 | Tâncu et al. (2019)^3^ | self-administered questionnaires (online) | closed | questionnaires distributed to a random group of patients in dental clinics | inclusion: adult dental patients in private clinics | ns | 14 days, max. 2 min | update | DB |
| IVa.117 | Tudorici et al. (2017) | self-administered questionnaires | closed | patients who attended the focussed department | inclusion: >20 years of age; from focussed region; with only permanent dentition | ns | ns | initial | DB |
| IVa.118 | Ungureanu et al. (2015) | computerized questionnaires (computer-assisted telephone interviews) | ns (closed, ns) | ns | inclusion: ≥18 years of age; patients visited a dentist in 12 months; national representative sample of patients; informed consents | ns | October 2012 to November 2012 | initial | DB |
| IVa.119 | Gao et al. (2020)^3^ | self-administered questionnaires (sent to parents); clinical data collected during examination | closed, scale | multistage cluster, random, equal proportion sampling method | inclusion: data of 3-5-year-old age groups | ns | August 2015 to December 2016 | update | DB |
| IVa.120 | Leung et al. (2010) | face-to-face interviews | open; WTP (bidding game) | random sample of people visiting clinic | inclusion: >16 years of age; Chinese citizen; informed consent | ns | ns | initial | DB |
| IVa.121 | Zhu et al. (2019)^3^ | (1) interviews; (2) questionnaire (online) | (1) semi-structured; (2) closed; DCE, WTP (ns) | random sample | inclusion: >18 years of age; Bejing residents; who had previously been treated for a dental condition | ns | October 2017 to December 2017 | update | DB |
| IVa.122 | Shanahan et al. (2017) | questionnaires | ns | participants were asked to fill out the questionnaire while waiting to be seen in the clinic | inclusion: >65 years of age; patients attending the focused geriatric medical outpatient clinic in March, April, and May 2016 | ns | March, April, and May 2016 | update | SR |
| IVa.123 | Al-Batayneh et al. (2019) | self-reported questionnaires | closed | convenient sample of families | inclusion: families of a healthy child, aged 2-12 years, with primary or mixed dentition, selected randomly among the family’s children where the oldest or the youngest child was chosen sequentially in the consenting family | gift (if cooperative behavior) | ns | update | DB |
| IVa.124 | Vermaire et al. (2012)^3^ | self-administered questionnaires (written) when the child visited a participating dental clinic | closed | participants from former study | inclusion: parents of children visiting a participating dental clinic in one of three large cities in the Netherlands | ns | September 2006 to September 2008 | update | SR |
| IVa.125 | Chebib et al. (2020)^3^ | questionnaire-based discrete choice experiment (DCE), WTP | ns; WTP (ns), WTT | participants were recruited from the patient pool of the institutions | inclusion: ≥65 years of age; speaking the local language fluently; independently living at home; namely French or German, for the Geneva and Bern group, respectively; exclusion: dependent participants with cognitive impairment; not sufficiently comprehend the local language; declined to provide informed consent | ns | ns | update | DB |
| IVa.126 | Meier et al. (2021) | questionnaires (written) | closed, scale | ns | exclusion: color-blindness; illegible handwriting | ns | ns | update | DB |
| IVa.127 | Sendi et al. (2017) | interviewer-administered questionnaires (telephone) | open; WTP (ns), closed, WTA | patients from previous study were sent information letters and then contacted by telephone calls | *criteria published elsewhere* | 50% discount on implant (about CHF 1,500) | 20 min | initial | DB |
| IVa.128 | Tianviwat et al. (2008) | interviewer-administered questionnaires | open; WTP (bidding game) | multistage cluster sampling with PPS, school as primary sampling unit | inclusion: parent (or other responsible adult) of primary school children in focussed region; agreed to participate | ns | April 2005 to September 2005 | initial | SR |
| IVa.129 | Tianviwat et al. (2009) | interviewer-administered questionnaires | *reported elsewhere* | parents were invited to participate by letter | inclusion: parents (or other responsible adults) of the children from previous survey | ns | ns | initial | SR |
| IVa.130 | Hof et al. (2014) | interviewer-administered questionnaires | closed | consecutive patients seeking implant treatment at the department | inclusion: informed consent; exclusion: patients with local or systemic contraindications to implant therapy or history of previous implant surgery | ns | ns | initial | SR |
| IVa.131 | Pommer et al. (2011) | interviewer-administered questionnaires | closed | sample was selected by pre-stratified multi-tiered cluster sampling using factor weighting for the variables sex, age, level of education, net monthly income, and size of residence | inclusion: >14 years of age (adults) | ns | July 20th, 2008, and August 10th, 2008 | update | SR |
| IVa.132 | van den Branden et al. (2013) | self-administered questionnaires | closed | stratified cluster sampling without replacement to select kindergarden in focussed region; all children of class 3 included | inclusion: 5 years of age (parents of children); children in focussed regions | ns | ns | initial | SR |
| IVa.133 | Atanasov et al. (2016) | self-administered questionnaires | closed; WTP (payment cards) | full list of all practicing dentists in the focussed city; random selection of 5 dentists who confirmed their willingness to take part | inclusion: ≥18 years of age | ns | April 2013 and May 2013 | initial | DB |
| IVa.134 | Lalabonova et al. (2015) | questionnaires | closed | ns | inclusion: patients referred for dental implant treatment because of partial or total loss of teeth; no contraindications that might preclude the chosen treatment | ns | ns | initial | DB |
| IVa.135 | Fernandez et al. (2015) | self-administered questionnaires | open, closed | patients in the waiting room were allocated a number from 1 to 10, each day 2 numbers were randomly selected | inclusion: ≥18 years of age; able to read and write; not under the influence of alcohol or drugs; had not previously undergone any surgery involving bone graft or bone augmentation; written free and informed consent | ns | January 2014 to July 2014 | initial | DB |
| IVa.136 | Rojas-Torres et al. (2019)^3^ | interviewer administered questionnaires | closed, open, conditional | patients from former study cohort were invited | inclusion: elderly patients; some degree of edentulism; informed consent | ns | July 2016 to October 2016, approx. 10 min | update | DB |
| IVa.137 | Amjad (2014) | interviewer-administered questionnaires | closed | ns | inclusion: ≥18 years of age; at least one tooth missing (excluding third molars); exclusion: did not give consent | ns | June 2012 to August 2012 | initial | SR |
| IVa.138 | Saleem et al. (2018) | self-administered questionnaires (given to patients in clinic) | ns | patients who showed interest was given a questionnaire to answer | inclusion: patients who arrived at outpatient clinic for the first time with past oral treatment history; showed interest was given to answer questionnaire | ns | April 2017 to September 2017 | update | DB |
| IVa.139 | Nair et al. (2016)^3^ | interviewer-administered questionnaires | closed; WTP (bidding game), scale | purposive sampling, sample of community dwelling elderly | inclusion: ≥60 years of age; informed consent | ns | 2010 and 2013 | update | SR |
| IVa.140 | Bajrić et al. (2015)^3^ | questionnaires | closed, scale | ns | inclusion: 8-, 12- and 15-year-olds; exclusion: patients with current signs and symptoms of acute odontalgia or any other urgent state in dentistry (bleeding, swelling, orodental trauma) | ns | ns | update | SR |
| IVa.141 | Widström et al. (2012) | self-administered questionnaires | open, closed; WTP (ns), ATP | random sample of subjects in focussed region selected by national statistics institution | inclusion: 47-59 years of age (adults); living in focused regions | ns | ns | initial | SR |
| IVa.142 | Chau et al. (2014)^3^ | questionnaires | open, closed | questionnaires were distributed in 8 participating randomly chosen schools; completed questionnaires were collected | inclusion: students at selected primary schools; class 2, and 5 | ns | ns | update | SR |
| IVa.143 | Abdulwahab et al. (2010) | interviewer-administered questionnaires (telephone) | closed | random digit-dialing telephone survey of the general population | inclusion: ≥18 years of age | ns | ns | initial | DB |
| IVa.144 | Malak et al. (2021)^3^ | questionnaires | ns, clinical data | multistage randomized cluster sampling; 1st stage: study sample drawn from a larger sample representative of school; 2nd stage: schools chosen at cluster level with PPS sampling method; 3rd stage: classes from selected schools | exclusion: children 12–15 years of age; non-Lebanese; with special needs | ns | September 2018 to June 2019 | update | DB |
| IVa.145 | Dalanon et al. (2018)^3^ | self-administered questionnaires (web-based) | closed | convenience sampling | ns | ns | ns | update | SR |
| IVa.146 | Wedrychowska-Szulc et al. (2010) | self-administered questionnaires | closed | recruited from the department and practices in focussed regions | inclusion: children and their parents/guardians | ns | June 2006 to December 2006 | initial | DB |
| IVa.147 | Tachalov et al. (2021) | survey | ns | ns | inclusion: 18-75 years of age; dental patients | ns | ns | update | DB |
| IVa.148 | Park et al. (2021)* | self-administered questionnaires | closed, scale | dental clinics selected by stratified random sampling, questionnaires given during visits to dental clinic | inclusion: >13 years of age; outpatients at dental clinics in Seoul; patients waiting to make a payment or receive their prescription after receiving treatment in the dental clinic | ns | December 1st, 2016, to December 30th, 2016 | update | DB |
| IVa.149 | Awooda et al. (2014) | interviewer-administered questionnaires | closed | non-probability convenience sampling technique | inclusion: ≥18 years of age; not inpatient; and no previous dental implant; informed written consent; exclusion: very old uncooperative patients; and children and mentally or physically disabled | ns | April 1st, 2013, to May 1st, 2013 | initial | SR |
| IVa.150 | Nyamuryekunge et al. (2018) | interviewer-administered questionnaires | open; WTP (ns) | outpatients in focussed hospitals were consecutively enlisted daily until sample size was reached; approached in waiting rooms | inclusion: ≥18 years of age; all outpatients | ns | 4-5 weeks | initial | DB |
| IVa.151 | Bucchi et al. (2019)^3^ | survey | closed, open | survey was administered to dental patients who were about to receive periodontal, surgical or implant treatment | inclusion: ≥18 years of age (adult); able to read and write in the local language; not under the influence of alcohol or drugs; had not previously undergone any surgery involving bone graft or bone augmentation; able to understand and follow the indications for the survey completion; willingness to participate in the study; able to provide informed consent | ns | 2017 and 2018 | update | DB |
| IVa.152 | Laothong et al. (2017) | self-administered questionnaires | closed | orthodontic patients collected from focussed hospitals | inclusion: ≥20 years of age; patients undergoing orthodontic treatment with upper and lower fixed orthodontic appliances but not in the retention stage; and with no craniofacial anomalies; willing to participate | ns | May to August 2016 | initial | DB |
| IVa.153 | Vernazza et al. (2015b) | self-administered questionnaires | ns; WTP (bidding game) | patients were recruited on a consecutive basis | inclusion: ≥40 years of age; patients attenting dental institutions; being at risk of root caries; using payment methods; completed questionnaire; exclusion: allergy to treatment components | ns | 4 weeks | initial | DB |
| IVa.154 | Walshaw et al. (2019) | self-administered questionnaires | ns, closed; WTP (bidding game) | consecutive sampling, patients attending focused clinics were invited | inclusion (Brazil): >18 years of age; with at least 1 natural tooth; utilised the SUS; capacity to consent; inclusion (UK): 6-16 years of age (parents), with at least 1 permanent tooth | ns | ns | update | DB |
| IVa.155 | Nalbandian et al. (2009) | questionnaires | closed | randomly from records of patients | inclusion: patients with completed treatment of 10 composite or 10 porcelain veneers | ns | ns | initial | DB |
| IVa.156 | Re et al. (2016)^3^ | dentist-administered  (1. part), and self-administered (2. part) questionnaire | closed; WTP (bidding game), scale | patients attending a private dental clinic were recruited for this study | inclusion: good general health (ASA I-II); with treatment less than 6 months earlier; exclusion: requiring an entire nerve block as a last procedure for pain control | ns | ns | update | DB |
| *b. Longitudinal studies* | | | | | | | | | |
| IVb.1 | Tilashalski et al. (2007) | interviewer-administered questionnaires (telephone) | closed | telephone screening methodology to identify persons who met eligibility criteria; stratified random sample of BL participants | inclusion: ≥45 years of age; at least one tooth | ns | 48 months | initial | DB |
| IVb.2 | Wall et al. (2015)^3^ | self-administered questionnaires (the home) | closed | data from nationally representative survey | inclusion: ≥2 years of age; with data for the years 2003-2004 and 2011-2012 | ns | ns | update | SR |
| IVb.3 | Arabi et al. (2019)^3^ | written questionnaires, computer-assisted personal interviews | closed, scale | probability samples; from two waves of a longitudinal, population-based survey | inclusion: ≥50 years of age; community-dwelling individuals and spouses; in Germany; exclusion: individuals incarcerated, hospitalized, or out of the country during whole study period; don’t speak country’s language, moved to unknown address; born 1962/1964 or earlier in wave 5/earlier | ns | 2013 and 2015 | update | DB |
| IVb.4 | Narby et al. (2008) | self-administered questionnaires | closed | randomly selected from official population register in focussed region | inclusion: participants still alive in second survey; still resident of focussed region | ns | 10 years | initial | SR |
| **V Mixed-methods studies** | | | | | | | | | |
| V.1 | Clarkson et al. (2020) | (1) semi-structured interviews,  (2) self-administered questionnaires (postal) | (1) open;  (2) open, closed | (1) convenience groups of dental professionals, (2) patients of focussed department and private practices requested to participate; dental specialists and general dentists requested to participate^4^ | (1) inclusion: dental professionals (general dentists, endodontists, periodontists, prosthodontists, and dental assistants/receptionists), (2) inclusion: patients of focussed department and private practices; dental specialists (endodontists, periodontists, prosthodontists, and oral and maxillofacial surgeons) and general dentists requested to paticipate (not included in no. of participants and results) | ns | (1) ns, (2) ns | initial | DB |
| V.2 | Harris et al. (2020) | (1)/(2) DCE / trial FU self-administered questionnaires (postal) | closed, scale | recruitment of patients through standard procedures and agreements for primary care research in the focussed nations; randomization: via telephone by automated central randomisation service | inclusion: patients who were dentate; had visited their dentist in the previous 2 years; received their dental care in part or fully as an NHS patient, including dental examination; exclusion: patients who had a medical condition indicating increased risk of bleeding; immunocompromised patients | £15 gift card (if FU attendance) | (1) July 7th, 2016, to August 5th, 2016; approx. 4-1,640 min; (2) 4 years; BL, and year 1, 2, 3, and 4 | update | DB |
| V.3 | Nayee et al. (2015) | (1) interviews (structure unclear), (2) questionnaires | (2) closed, scale | dental clinics selected using random sampling, divided between care units; selection of users came from records of the PCIS of the municipal health department | inclusion: ≥18 years of age; not have any physical or mental disability capable of compromising the interview; being registered with the sampled unit for at least two years | ns | ns | update | DB |
| V.4 | Paisi et al. (2020) | (1) retrospective study,  (2) interviews | (1) closed;  (2) closed, unclear | (1) all outpatients' visits for 1 year, data obtained from hospital information system; (2) database of scheduled appointment, constructed by hospital information system; random selection of cases and controls in waiting region of at selected clinics that show the highest patient volume | (1) inclusion: patients categorized into appeared and missed visits.; (2) inclusion: ≥18 years of age; patients of outpatient clinics with more than 10.000 visits a year; cases: who had at least one missed appointment during the previous 6 months to the current appointment; controls: *same criteria, but* who had no missed appointment; informed consent; exclusion: psychiatry clinics | ns | (1) 1 year, (2) March 2008 to May 2008 | initial | SR |
| V.5 | El-Din (2008) | (1) systematic review; (2) randomised controlled trial; (3) observations, in-depth interviews (telephone) | (1) literature search;  (2) open, closed; WTP (payment cards), clinical data; (3) semi-structured | (1) electronic searches of nine databases, hand-searching of eight specialist journals; (2)(3) RCT: recruited from four NHS dental practices; practices were invited to participate by sequential invitation based on random selection from a list of NHS dental practices | inclusion RCT: ≥18 years of age; high/medium (red/amber) risk of poor oral health; NHS patients; new patients or regular attenders; any level of literacy; exclusion: low (green) risk based on the absence of either clinical- or patient-related factors; patients attending for an emergency appointment (they do not usually receive a full check-up); edentulous; private patients; patients requiring an interpreter for routine care | no financial incentive, £25 prize lottery (if FU attendance) | (2) BL, short-term (3 months), medium-term (6 months) and long-term (12 months) FU; (3) medium-term (6 months) and long-term (12 months) FU | update | DB |
| V.6 | Milner et al. (2019) | (1) literature review; (2) self-administered questionnaires | (1) closed,  (2) closed | (1) dental experts contacted via telephone; (2) distribution to inbound tourists in dental clinic | (1) inclusion: dental experts with work experience related to dental tourism, patient satisfaction surveys, and dental care services for inbound tourists in private dental clinics; within the scope of work related to legislation and its enforcement; informed consent; (2) inclusion: ≥18 years of age; tourists staying ≥1 night, receiving services at private dental clinics in focussed region; exclusion: expatriates and foreigners residing in focussed region | ns | (1)/(2) May 2015 to November 2015 | initial | DB |
| V.7 | Papautsky et al. (2021) | (1) interviewer-administered questionnaires, (2) in-depth interviews | (1) closed,  (2) open | (1) children and parents/caregiver visiting public health service in focussed region, right after treatment; (2) children and parents visiting a health | (1) inclusion: children with occlusal and proximal caries in primary molars; without pulp involvements; exclusion: certain clinical values; cavity criteria class I or II^3^; (2) inclusion: children and parents visiting health unit for treatment; informed consent | ns | (1) October 2014 to July 2015, (2) ns | initial | DB |
| V.8 | Da Kfouri et al. (2019) | choice scenarios: questionnaires (online) | closed, scale | (1)/(2) recruited by an online provider | ns | financial incentive | ns | update | DB |
| V.9 | Maciel et al. (2017) | (1)/(2) self-administered questionnaires | (1)/(2) open, closed | (1)/(2) questionnaire given while registering in dental institution | (1)/(2) inclusion: patients in dental institution | ns | (1)/(2) 4 weeks | initial | DB |
| V.10 | Azarpazhooh et al. (2016) | (1) retrospective assessment of anonymous patient data;  (2) face-to-face interviews | (1) ns, closed, clinical data; (2) semi-structured | (1) ns; (2) purposeful sampling, approached through gatekeeper | ns | ns | (2) September 2018 to February 2020, 20-45 min | update | DB |
| V.11 | Schwendicke et al. (2016) | (1)/(2) survey, self-administered questionnaires (web-based) | open, closed | invited through social media providers, listservs, social networks, and websites (e.g., of institution) | ns | no incentive | approx. 35 minutes | update | DB |
| V.12 | Sever et al. (2018)^3^ | (1) focus group interviews,  (2) self-administered questionnaires | (1) open,  (2) closed | (1) convenience sample, recruited face-to-face by investigator at dental institution in focussed regions; (2) convenience, snowball, and deviant-case sampling; questionnaire given by nurse in waiting rooms | (1) inclusion: diverse sample regarding gender, age, educational status; verbal and written consent; (2) inclusion: patients in focussed institutions | no | (1)/(2) March 2014 to March 2015 | initial | DB |
| V.13 | Sonneveld et al. (2013) | (1) face-to-face in-depth interviews;  (2) paper-based questionnaires, WTP | (1) ns;  (2) closed; WTP (ns) | (1)/(2) initial sample selection at appointment desk, systematically allocated to groups by faculty member | (1)/(2) inclusion: patients who attended clinic | toothbrush, toothpaste | (1)/(2) March 2016 to January 2017 | initial | DB |
| V.14 | Jaapar et al. (2017) | (1) focus group interviews,  (2) self-administered questionnaires | (1) closed,  (2) closed | (1) randomly selected via patient platform; (2) patients visiting practice; multi-stage-sampling: dentists randomly chosen from community registry; 1st 50 patients treated by dentist in period | (1) inclusion: patients on patient platform;  (2) inclusion: patients of selected dentists, respondents who chose between 8 and 12 aspects (out of 41) in questionnaire | ns | (1) ns, (2) ns | initial | DB |
| **Legend:** ^1^ order of references according to Table A6; ^2^ details if available; ^3^ included in cross-check (n = 45 articles, n = 2 articles excluded afterwards from pool of relevant articles); ^4^ not included in no. of participants and results; ACR – American College of Rheumatology; ASA – American Society of Anaesthesiologists/physical status classification system; BL – baseline; BPE – basic periodontal examination; CAD – Canadian dollars; CLP – cleft lip palate; DB – database literature search; DCE – discrete-choice experiment; ED – emergency department; EPV – Event per Variable; FDV – 1st dental visit; FMBS – full mouth bleeding score; FMPS – full mouth plaque score; FU – follow-up; PPS – probability proportional to size; MBM – Market Basket Measure; NHS – National Health Service (UK and Northern Ireland); ns – not stated or unclear; PCIS – Primary Care Information System; RCT – randomized controlled trial; SR – screening of article titles in systematic reviews' literature lists; SUS – Brazilian Unified Health System; TNM – Tumor–Node–Metastasis classification system (for malignant tumours); WTA – willingness-to-accept, incl. approach; WTP – willingness-to-pay; WTT – willingness-to-travel; ZZP – Package of Care Dependency/scale on level and type of care needed | | | | | | | | | |
