# Supplementary material for: Decision-making regarding dental treatments – What factors matter from patients’ perspective? A systematic review
Source: BMC Oral Health. 2025 Nov 25;26:289. doi: 10.1186/s12903-025-07032-9 (PMC12903421; doi:10.1186/s12903-025-07032-9)
Supplement: Supplementary file 1 — Additional file 1: A1. Guideline on literature search, selection, and analysis. A2. Search strategy. A3. PRISMA checklist. A4. SWiM checklist. A5. Search strings for databases, including hits. A6. Characteristics, factors of choice, and references of included articles (N = 233), sorted by number of identified articles per country (descending) within study designs I–V. A7. Methodological characteristics of included articles (N = 233), and search details. A8. Coding scheme, codebook, and framework, including definitions of excluded and summarized codes. A9. Code definitions. A10. Calculation of ICA and ICR. A11. Quality assessment by MMAT: study design I. A12. Quality assessment by MMAT: study design II. A13. Quality assessment by MMAT: study design III. A14. Quality assessment by MMAT: study design IV. A15. Quality assessment by MMAT: study design V. A16. MMAT assessment results description. [file 12903_2025_7032_MOESM1_ESM.zip › A8_Coding_scheme_codebook_and_framework.docx]

**A8.** Coding scheme, codebook, and framework, including definitions of excluded and summarized codes

| **Coding scheme** | | | | | | | | | |
| --- | --- | --- | --- | --- | --- | --- | --- | --- | --- |
| category I   - code 1 - code 2 - new code names - - codes   subcategory I.1   - code 1 - code 2 - code 3 - … - code 5 = code 1+2 - code 3 - code 4 - ... - code 1 - code 2 - + codes - - codes - code 1 - code 2 - + codes - - codes   subcategory I.2  …  … | | | | | | | | | |
| **Codebook** | | | | | | | | | |
| **Framework: codes version 1, before  1. coding (n = 176)** | **Codes version 2, after 1. coding**  **(n = 189)** | **Freq.** | **Codes version 3, after 2. coding**  **(n = 181)** | **Freq.** | **Code version 4, after consensus**  **(n = 181)** | **Freq.** | **Summarization of codes & categorization to factors (n = 101)** | **Subcategories**  **(n = 9)** | **Categories**  **(n = 3)** |
| access barriers | access barriers | 47 | access barriers | 49 | access barriers | 30 | access barriers | **Access to care** | **Dentist & dental institution** |
| treatment availability | treatment availability | 3 | treatment availability | 3 | treatment availability | 1 |  |  |  |
| availability | availability | 10 | availability | 10 | availability | 5 |  |  |  |
| lack of dentist | lack of dentist | 10 | lack of dentist | 12 | lack of dentist | 8 |  |  |  |
|  | limited resources*^#^ | 3 | limited resources | 3 | limited resources | 1 |  |  |  |
|  | barriers*^#^ | 11 |  |  |  |  |  |  |  |
| after care | after care | 10 | after care | 12 | after care | 7 | after care |  |  |
| insurance | insurance | 44 | insurance | 48 | insurance | 26 | insurance |  |  |
| infrastructure | infrastructure | 2 | infrastructure | 2 | infrastructure | 4 | location |  |  |
| location | location | 56 | location | 55 | location | 33 |  |  |  |
| alternative treatment methods | alternative treatment methods | 20 | alternative treatment methods | 21 | alternative treatment methods | 15 | other/different alternative |  |  |
| other/different alternative | other/different alternative | 2 | other/different alternative | 3 | other/different alternative | 2 |  |  |  |
| personalized care | personalized care | 1 | personalized care | 1 | personalized care | 3 | personalized care |  |  |
| guideline-oriented working | guideline-oriented working | 10 | guideline-oriented working | 10 | guideline-oriented working | 9 | quality of care |  |  |
| hygiene | hygiene | 16 | hygiene | 14 | hygiene | 6 |  |  |  |
| quality of care | quality of care | 54 | quality of care | 53 | quality of care | 24 |  |  |  |
| safety | safety | 14 | safety | 14 | safety | 9 |  |  |  |
| specialties in dental practice | specialties in dental practice | 4 | specialties in dental practice | 4 | specialties in dental practice | 2 | specialties in dental practice |  |  |
| transportation | transportation | 31 | transportation | 33 | transportation | 21 | transportation |  |  |
| altruism | altruism | 4 | altruism | 4 | altruism | 2 | altruism | **Communication** |  |
|  | communication skills* | 9 | communication skills | 9 | communication skills | 9 | communication skills |  |  |
|  | dentist's responsiveness* | 3 | dentist's responsiveness | 3 | dentist's responsiveness | 5 | dentist's responsiveness |  |  |
| family orientation | family orientation | 1 | family orientation | 1 | family orientation | 1 | family orientation |  |  |
| intrusiveness of dentist and staff | intrusiveness of dentist and staff | 4 | intrusiveness of dentist and staff | 5 | intrusiveness of dentist and staff | 3 | intrusiveness of dentist and staff |  |  |
| communication with dentist | communication with dentist | 34 | communication with dentist | 34 | communication with dentist | 12 | provided information |  |  |
|  |  |  |  |  | communication with staff* | 1 |  |  |  |
| dentist provides information | dentist provides information | 39 | dentist provides information | 40 | dentist provides information | 26 |  |  |  |
| provided information | provided information | 26 | provided information | 31 | provided information | 24 |  |  |  |
| explanation of treatment | explanation of treatment | 1 | explanation of treatment^#^ | 0 |  |  |  |  |  |
| extensive offer of treatment | extensive offer of treatment | 1 | extensive offer of treatment | 1 | extensive offer of treatment | 0 |  |  |  |
| relationship to dentist | relationship to dentist | 25 | relationship to dentist | 25 | relationship to dentist | 12 | relationship to professional |  |  |
| relationship to staff | relationship to staff | 16 | relationship to staff | 16 | relationship to staff | 7 |  |  |  |
| respect | respect | 3 | respect | 4 | respect | 3 | respect |  |  |
| community orientation | community orientation | 3 | community orientation | 3 | community orientation | 1 | social competence of professionals |  |  |
| cultural competence | cultural competence | 3 | cultural competence | 3 | cultural competence | 3 |  |  |  |
| social competence of dentist | social competence of dentist | 24 | social competence of dentist | 26 | social competence of dentist | 16 |  |  |  |
| social competence of staff | social competence of staff | 18 | social competence of staff | 22 | social competence of staff | 9 |  |  |  |
| signs of service areas | signs of service areas^#^ | 0 |  |  |  |  |  |  |  |
| staff behavior | staff behavior | 19 | staff behavior | 19 | staff behavior | 14 | staff behavior |  |  |
| clarity of responsibilities | clarity of responsibilities | 3 | clarity of responsibilities | 3 | clarity of responsibilities | 2 | transparency |  |  |
| transparency | transparency | 1 | transparency | 1 | transparency | 1 |  |  |  |
|  | trust in dentistry* | 1 | trust in dentistry | 1 | trust in dentistry | 2 | trust |  |  |
| trust in dentists | trust in dentists | 29 | trust in dentists | 29 | trust in dentists | 18 |  |  |  |
| trust in staff | trust in staff | 8 | trust in staff | 8 | trust in staff | 5 |  |  |  |
| language | language | 7 | language | 7 | language | 6 | understandable information |  |  |
| understandable information | understandable information | 4 | understandable information | 4 | understandable information | 4 |  |  |  |
| academic institution | academic institution | 8 | academic institution | 8 | academic institution | 5 | academic institution | **Qualification** |  |
| continuous professional development | continuous professional development^#^ | 0 |  |  |  |  |  |  |  |
| dentist attire | dentist attire | 34 | dentist attire | 34 | dentist attire | 18 | dentist attire |  |  |
| dentist behavior | dentist behavior | 13 | dentist behavior | 12 | dentist behavior | 6 | dentist behavior |  |  |
| dentist characteristics | dentist characteristics | 45 | dentist characteristics | 45 | dentist characteristics | 19 | dentist characteristics |  |  |
| accuracy | accuracy | 1 | accuracy | 1 | accuracy | 1 | dentist professional skills |  |  |
|  | confidentiality* | 1 | confidentiality | 1 | confidentiality | 3 |  |  |  |
| dentist competence | dentist competence | 23 | dentist competence | 27 | dentist competence | 18 |  |  |  |
| dentist experience | dentist experience | 8 | dentist experience | 8 | dentist experience | 4 |  |  |  |
| dentist professional skills | dentist professional skills | 12 | dentist professional skills | 12 | dentist professional skills | 4 |  |  |  |
| reliability | reliability | 3 | reliability | 4 | reliability | 4 |  |  |  |
| dentist qualification | dentist qualification | 31 | dentist qualification | 31 | dentist qualification | 17 | dentist qualification |  |  |
| feedback culture | feedback culture | 0 | feedback culture | 0 | feedback culture | 1 | feedback culture |  |  |
| quality assessment culture | quality assessment culture | 6 | quality assessment culture | 6 | quality assessment culture | 1 |  |  |  |
|  |  |  |  |  | medical error* | 1 | medical error |  |  |
| racism | racism | 3 | racism | 4 | racism | 3 | racism |  |  |
| staff qualification | staff qualification | 4 | staff qualification | 4 | staff qualification | 2 | staff qualification |  |  |
|  | staff attire* | 1 | staff attire | 1 | staff attire^#^ | 0 |  |  |  |
| technical qualifications | technical qualifications^#^ | 0 |  |  |  |  |  |  |  |
| collaboration with other medical professionals | collaboration with other medical professionals | 6 | collaboration with other medical professionals | 6 | collaboration with other medical professionals | 5 | collaboration with other medical professionals | **Organization** |  |
| customer service | customer service | 39 | customer service | 53 | customer service | 28 | customer service |  |  |
| documentation | documentation | 4 | documentation | 4 | documentation | 4 | documentation |  |  |
| emergency service availability | emergency service availability | 6 | emergency service availability | 6 | emergency service availability | 2 | emergency service availability |  |  |
| access to facility | access to facility | 11 | access to facility | 11 | access to facility | 5 | facilities |  |  |
| facilities | facilities | 37 | facilities | 37 | facilities | 22 |  |  |  |
| parking | parking | 3 | parking | 3 | parking | 3 |  |  |  |
| scented operatory | scented operatory | 1 | scented operatory | 1 | scented operatory | 1 |  |  |  |
| waiting room facilities | waiting room facilities | 14 | waiting room facilities | 15 | waiting room facilities | 8 |  |  |  |
| flexibility | flexibility | 1 | flexibility | 1 | flexibility | 1 | flexibility |  |  |
| continuity of care | continuity of care | 4 | continuity of care | 4 | continuity of care | 2 | follow-up care |  |  |
| follow-up care | follow-up care | 12 | follow-up care | 11 | follow-up care | 6 |  |  |  |
| organization | organization | 4 | organization | 5 | organization | 6 | organization |  |  |
| overall impression | overall impression | 3 | overall impression | 3 | overall impression | 3 | overall impression |  |  |
| payment modality | payment modality | 12 | payment modality | 12 | payment modality | 5 | payment modality |  |  |
| referred by professional | referred by professional | 20 | referred by professional | 20 | referred by professional | 10 | referred by professional |  |  |
| appointment reminder | appointment reminder | 6 | appointment reminder | 6 | appointment reminder | 3 | time management of professional |  |  |
| time management of professional | time management of professional | 62 | time management of professional | 64 | time management of professional | 36 |  |  |  |
| waiting time for appointment | waiting time for appointment | 28 | waiting time for appointment | 28 | waiting time for appointment | 14 |  |  |  |
| delay of treatment | delay of treatment | 4 | delay of treatment | 4 | delay of treatment | 3 |  |  |  |
| emergency | emergency | 9 | emergency | 9 | emergency | 8 | emergency | **Medical characteristics** | **Patient** |
| health status | health status | 36 | health status | 34 | health status | 21 | health status |  |  |
| life-course perspective | life-course perspective | 7 | life-course perspective | 7 | life-course perspective | 5 | life-course perspective |  |  |
| medical need | medical need | 24 | medical need | 30 | medical need | 24 | medical need |  |  |
| special treatment necessary | special treatment necessary | 7 | special treatment necessary | 10 | special treatment necessary | 6 |  |  |  |
| specialist | specialist | 24 | specialist | 20 | specialist | 12 |  |  |  |
| psychological needs of patient | psychological needs of patient | 12 | psychological needs of patient | 12 | psychological needs of patient | 3 |  |  |  |
| physical needs of patient | physical needs of patient | 52 | physical needs of patient | 61 | physical needs of patient | 31 | physical needs of patient |  |  |
| prevention | prevention | 29 | prevention | 33 | prevention | 20 | prevention |  |  |
| quality of life | quality of life | 9 | quality of life | 9 | quality of life | 4 | quality of life |  |  |
| sickness/unwellness | sickness/unwellness | 21 | sickness/unwellness | 21 | sickness/unwellness | 12 | sickness/unwellness |  |  |
| appointment was not with the doctor of choice | appointment was not with the doctor of choice | 6 | appointment was not with the doctor of choice | 6 | appointment was not with the doctor of choice | 2 | dentist of choice | **Social characteristics** |  |
| dentist rotation | dentist rotation | 4 | dentist rotation | 4 | dentist rotation | 2 |  |  |  |
| animal testing | animal testing | 3 | animal testing | 4 | animal testing | 2 | ethics |  |  |
| ethics | ethics | 8 | ethics | 9 | ethics | 4 |  |  |  |
| improve professional opportunities | improve professional opportunities | 5 | improve professional opportunities | 4 | improve professional opportunities | 3 | improve professional opportunities |  |  |
| oral health awareness | oral health awareness | 48 | oral health awareness | 47 | oral health awareness | 21 | oral health awareness |  |  |
| religion | religion | 5 | religion | 6 | religion | 3 | religion |  |  |
| school requirement | school requirement | 4 | school requirement | 5 | school requirement | 4 | school requirement |  |  |
| self-esteem | self-esteem | 24 | self-esteem | 30 | self-esteem | 19 | self-esteem |  |  |
| shame | shame | 4 | shame | 4 | shame | 4 |  |  |  |
| impact of war | impact of war | 1 | impact of war | 1 | impact of war | 1 | social environment |  |  |
| personal circumstances | personal circumstances | 5 | personal circumstances | 5 | personal circumstances | 4 |  |  |  |
| professional authority | professional authority | 4 | professional authority | 4 | professional authority | 3 |  |  |  |
| social class | social class | 2 | social class | 2 | social class | 1 |  |  |  |
| social environment | social environment | 81 | social environment | 36 | social environment | 17 |  |  |  |
| social pressure | social pressure | 18 | social pressure | 22 | social pressure | 12 |  |  |  |
| social security | social security | 1 | social security | 1 | social security | 1 |  |  |  |
|  | social self*^#^ | 8 |  |  |  |  |  |  |  |
| social isolation | social isolation | 14 | social isolation | 21 | social isolation | 11 | social isolation |  |  |
| acceptance | acceptance | 24 | acceptance | 26 | acceptance | 20 | acceptance | **Individual characteristics** |  |
| aesthetics | aesthetics | 109 | aesthetics | 116 | aesthetics | 64 | aesthetics |  |  |
| teeth location | teeth location | 4 | teeth location | 4 | teeth location | 3 |  |  |  |
| tooth color | tooth color | 14 | tooth color | 14 | tooth color | 8 |  |  |  |
| appearance | appearance | 26 | appearance | 25 | appearance | 13 | appearance |  |  |
|  | anxiety*^#^ | 14 |  |  |  |  |  |  |  |
| comfort | comfort | 39 | comfort | 44 | comfort | 27 | comfort |  |  |
| complaints during treatment | complaints during treatment | 1 | complaints during treatment | 1 | complaints during treatment | 0 |  |  |  |
| convenience | convenience | 25 | convenience | 32 | convenience | 17 | convenience |  |  |
| usability | usability | 1 | usability | 1 | usability | 1 |  |  |  |
| visiting friends/relatives in area | visiting friends/relatives in area | 2 | visiting friends/relatives in area | 2 | visiting friends/relatives in area | 2 |  |  |  |
| easy access to modes of non-doctor treatments (e.g., drugs) | easy access to modes of non-doctor treatments (e.g., drugs) | 6 | easy access to modes of non-doctor treatments (e.g., drugs) | 6 | easy access to modes of non-doctor treatments (e.g., drugs) | 4 | easy access to modes of non-doctor treatments (e.g., drugs) |  |  |
| expectation | expectation | 12 | expectation | 15 | expectation | 10 | experience by patient |  |  |
| experience by patient | experience by patient | 101 | experience by patient | 100 | experience by patient | 49 |  |  |  |
| fear | fear | 130 | fear | 138 | fear | 73 | fear |  |  |
| forgetfulness | forgetfulness | 10 | forgetfulness | 10 | forgetfulness | 5 | forgetfulness |  |  |
| handicap | handicap* |  |  |  |  |  |  |  |  |
| health-seeking behavior | health seeking behavior | 98 | health seeking behavior | 54 | health-seeking behavior | 35 | health-seeking behavior |  |  |
| income | income | 3 | income | 1 | income^#^ | 0 |  |  |  |
| attractiveness travel/tourism package | attractiveness travel/tourism package | 5 | attractiveness travel/tourism package | 5 | attractiveness travel/tourism package | 4 | medical tourism |  |  |
| willingness-to-travel | willingness-to-travel | 1 | willingness-to-travel | 2 | willingness-to-travel | 1 |  |  |  |
| pain | pain | 106 | pain | 110 | pain | 61 | pain |  |  |
| patient characteristics | patient characteristics | 66 | patient characteristics | 39 | patient characteristics | 20 | patient characteristics |  |  |
| sensitive | sensitive | 8 | sensitive | 8 | sensitive | 7 |  |  |  |
| knowledge | knowledge | 22 | knowledge | 30 | knowledge | 15 | patient knowledge |  |  |
| patient knowledge | patient knowledge | 82 | patient knowledge | 79 | patient knowledge | 40 |  |  |  |
| frustration | frustration | 2 | frustration | 2 | frustration | 2 | patient motivation |  |  |
| importance | importance | 7 | importance | 6 | importance | 9 |  |  |  |
| motivation | motivation | 10 | motivation | 6 | motivation | 5 |  |  |  |
| patient attitude | patient attitude | 6 | patient attitude | 6 | patient attitude | 5 |  |  |  |
| patient motivation | patient motivation | 47 | patient motivation | 24 | patient motivation | 14 |  |  |  |
|  | patient preference*^#^ | 27 |  |  |  |  |  |  |  |
| recommendation by professional | recommendation by professional | 20 | recommendation by professional | 23 | recommendation by professional | 17 | recommendation |  |  |
| recommendation of others | recommendation of others | 31 | recommendation of others | 36 | recommendation of others | 26 |  |  |  |
| SARS-CoV-2 infection | SARS-CoV-2 infection | 2 | SARS-CoV-2 infection | 2 | SARS-CoV-2 infection | 1 | SARS-CoV-2 infection |  |  |
| self-diagnosis | self-diagnosis | 53 | self-diagnosis | 82 | self-diagnosis | 43 | self-diagnosis |  |  |
| source of information | source of information | 76 | source of information | 76 | source of information | 36 | source of information |  |  |
|  | stuck in traffic* | 1 | stuck in traffic | 1 | stuck in traffic | 1 | time management of patient |  |  |
| time off work required | time off work required | 14 | time off work required | 14 | time off work required | 10 |  |  |  |
| time management of patient | time management of patient | 56 | time management of patient | 56 | time management of patient | 28 |  |  |  |
| adaptation with alternative | adaptation with alternative | 4 | adaptation with alternative | 5 | adaptation with alternative | 4 | adaptation with alternative | **Treatment characteristics** | **Treatment** |
|  | apps* | 1 | apps^#^ | 0 |  |  |  |  |  |
| complexity of treatment | complexity of treatment | 7 | complexity of treatment | 8 | complexity of treatment | 4 | complexity of treatment |  |  |
| ease of cleaning | ease of cleaning | 5 | ease of cleaning | 5 | ease of cleaning | 4 |  |  |  |
| simplicity | simplicity | 2 | simplicity | 2 | simplicity | 2 |  |  |  |
| complicated treatment | complicated treatment | 1 | complicated treatment | 1 | complicated treatment | 1 | complicated treatment |  |  |
| consequences (medical) | consequences (medical) | 39 | consequences (medical) | 42 | consequences (medical) | 24 | consequences |  |  |
| consequences (non-medical) | consequences (non-medical) | 9 | consequences (non-medical) | 8 | consequences (non-medical) | 5 |  |  |  |
| complications | complications | 17 | complications | 22 | complications | 15 | complications |  |  |
| diagnostic care | diagnostic care | 5 | diagnostic care | 5 | diagnostic care | 3 | diagnostic care |  |  |
| durability | durability | 7 | durability | 7 | durability | 5 | durability |  |  |
| duration of treatment | duration of treatment | 55 | duration of treatment | 61 | duration of treatment | 34 | duration of treatment |  |  |
| extent of treatment | extent of treatment | 17 | extent of treatment | 21 | extent of treatment | 13 |  |  |  |
| time duration of session | time duration of session | 12 | time duration of session | 12 | time duration of session | 10 |  |  |  |
| treatment period | treatment period | 1 | treatment period | 1 | treatment period | 1 |  |  |  |
| efficient treatment | efficient treatment | 11 | efficient treatment | 13 | efficient treatment | 11 | efficient treatment |  |  |
| functionality | functionality | 59 | functionality | 67 | functionality | 41 | functionality |  |  |
| medical equipment | medical equipment | 32 | medical equipment | 36 | medical equipment | 19 | medical equipment |  |  |
| medicine as alternative | medicine as alternative | 5 | medicine as alternative | 5 | medicine as alternative | 5 | medicine as alternative |  |  |
| naturality | naturality | 10 | naturality | 10 | naturality | 8 | naturality |  |  |
| impact on general health | impacts on general health | 12 | impacts on general health | 13 | impact on general health | 7 | outcome |  |  |
| outcome | outcome | 33 | outcome | 37 | outcome | 16 |  |  |  |
| satisfaction | satisfaction | 35 | satisfaction | 35 | satisfaction | 22 |  |  |  |
| treatment success | treatment success | 8 | treatment success | 9 | treatment success | 11 |  |  |  |
| risk | risk | 19 | risk | 24 | risk | 15 | risk |  |  |
| tooth saving | tooth saving | 34 | tooth saving | 20 | tooth saving | 11 | tooth saving |  |  |
| healing time | healing time | 1 | healing time | 1 | healing time | 2 | treatment characteristics |  |  |
| instability | instability | 1 | instability | 1 | instability | 1 |  |  |  |
| invasiveness | invasiveness | 4 | invasiveness | 5 | invasiveness | 4 |  |  |  |
| longevity | longevity | 15 | longevity | 17 | longevity | 10 |  |  |  |
| material | material | 10 | material | 14 | material | 10 |  |  |  |
|  | surgery necessary* | 7 | surgery necessary | 9 | surgery necessary | 8 |  |  |  |
| treatment characteristics | treatment characteristics | 83 | treatment characteristics | 29 | treatment characteristics | 19 |  |  |  |
| cost | cost | 292 | cost | 294 | cost | 151 | out-of-pocket payment | **Cost** |  |
| for second opinion | for second opinion | 1 | for second opinion | 2 | for second opinion | 1 | for second opinion |  |  |
| instalments | instalments | 3 | instalments | 3 | instalments | 2 | instalments |  |  |
| **Legend:** * new codes emerging from coding and consensus discussion, ^#^ excluded codes due to consensus discussion or frequency in coding n = 0, Freq. – frequency of codes used in coding by both coders (= reviewers) | | | | | | | | | |
| **Excluded or summarized codes** | | **Definition:** | | | | | | | |
| access to facility | | ... access to dental institution, e.g., wheelchair level. | | | | | | | |
| accuracy | | ... exact working method of dentist. | | | | | | | |
| alternative treatment methods | | ... existence of (dental) treatment alternatives. | | | | | | | |
| animal testing | | ... patient’s attitude towards animal experiments. | | | | | | | |
| anxiety | | ... fear of a condition as a personality trait. | | | | | | | |
| appointment reminder | | ... (automatic) reminder of the patient (e.g., by cell phone message) that her/his treatment appointment is due soon. | | | | | | | |
| appointment was not with the doctor of choice | | ... that treatment was not performed by the desired dentist. | | | | | | | |
| apps | | ... software used in form of an app as part of dental treatment. | | | | | | | |
| attractiveness travel/tourism package | | ... (organized) amenities of dental tourism. | | | | | | | |
| availability | | ... offer of dental treatment, so that it could be perceived by the patient. | | | | | | | |
| barriers | | ... any circumstances that make it difficult to access dental treatment or care. | | | | | | | |
| clarity of responsibilities | | ... patient’s overview of responsibilities of dental professionals involved in dental treatment. | | | | | | | |
| communication skills | | ... skills of dentist and dental staff regarding their communication. | | | | | | | |
| communication with dentist | | ... verbal and non-verbal communication between patient and dentist. | | | | | | | |
| communication with staff | | ... verbal and non-verbal communication between patient and dental staff. | | | | | | | |
| community orientation | | ... non-profit nature of dental service. | | | | | | | |
| complaints during treatment | | ... patient’s discomfort during dental treatment. | | | | | | | |
| confidentiality | | ... confidential handling and secrecy of patient data, including implementation of data protection measures in dental institution. | | | | | | | |
| consequences (medical) | | ... occurrence of foreseeable events after dental treatment requiring further medical intervention. | | | | | | | |
| consequences (non-medical) | | ... occurrence of foreseeable events after dental treatment requiring non-medical interventions. | | | | | | | |
| continuity of care | | ... need for treatment to be continued. | | | | | | | |
| continuous professional development | | ... constant visible progress of dentist expertise and dental institution conditions. | | | | | | | |
| cultural competence | | ... consideration of patient’s cultural needs within dental treatment. | | | | | | | |
| delay of treatment | | ... postponement of dental treatment to another date. | | | | | | | |
| dentist competence | | ... professional expertise of dentist attributed by patient. | | | | | | | |
| dentist experience | | ... experience of dentist measured in units, e.g., number of professional years or dental treatments. | | | | | | | |
| dentist provides information | | ... information provided by dentist about dental treatment, e.g., through conversation with patient. | | | | | | | |
| dentist rotation | | ... change of dentists for practice-organizational reasons during patient contact. | | | | | | | |
| dentist's responsiveness | | ... responsibility of dentist regarding dental treatment and her/his decisions towards the patient. | | | | | | | |
| ease of cleaning | | ... effort required for patient’s (daily) toothbrushing routine. | | | | | | | |
| expectation | | ... patient’s expectations of dental treatment, dentist, or dental institution. | | | | | | | |
| explanation of treatment | | ... explanation of dental treatment, e.g., regarding extent and result. | | | | | | | |
| extensive offer of treatment | | ... emphatic request by the dentist or dental staff to agree to dental treatment. | | | | | | | |
| extent of treatment | | ... extent of dental treatment, measured in defined units, e.g., months, sessions. | | | | | | | |
| frustration | | ... feeling of disappointment and powerlessness after an experience regarding dental treatment by patient. | | | | | | | |
| guideline-oriented working | | ... dentist works according to latest scientific standards and follows dental medical guidelines. | | | | | | | |
| handicap | | ... physical or mental impairment of patient. | | | | | | | |
| healing time | | ... length of time after dental treatment until a wound has healed or patient has no complaints caused by dental treatment,   e.g., surgical incision. | | | | | | | |
| hygiene | | ... implementation of hygiene measures in dental institution. | | | | | | | |
| impact of war | | ... influence of war on dental service utilization. | | | | | | | |
| impact on general health | | ... impact on patient’s overall physical and psychological health condition. | | | | | | | |
| importance | | ... conditions perceived as personally important. | | | | | | | |
| income | | ... patient’s financial income through employment or from other sources, e.g., unemployment compensation. | | | | | | | |
| infrastructure | | ... patient’s connection possibilities from her/his place of residence or work to dental institution, e.g., via bus or train. | | | | | | | |
| instability | | ... result of dental treatment feels unstable or insecure. | | | | | | | |
| invasiveness | | ... degree of invasiveness involved in dental treatment. | | | | | | | |
| knowledge | | ... extent of (dental) medical expertise of patient. | | | | | | | |
| lack of dentist | | ... unavailability of dentist as dental service provider. | | | | | | | |
| language | | ... communication by language between patient, dentist, and staff. | | | | | | | |
| limited resources | | ... material and technical limitations regarding implementation of dental treatment. | | | | | | | |
| longevity | | ... expected durability of dental treatment (result). | | | | | | | |
| material | | ... material used in dental treatment (e.g., dental filling, instruments) and its attributes. | | | | | | | |
| medical error | | ... dental medical errors and mistreatment | | | | | | | |
| motivation | | ... totality of patient’s motivations that conditions decision or action. | | | | | | | |
| parking | | ... parking facilities near dental institution. | | | | | | | |
| patient attitude | | ... attitude of patient, e.g., expressed by gestures and facial expressions. | | | | | | | |
| patient motivation | | ... totality of patient's motivations that influences her/his decision or action to improve her/his health status, including self-diagnosis. | | | | | | | |
| patient preference | | ... patient's preference for certain features. | | | | | | | |
| personal circumstances | | ... circumstances affecting patient’s daily life, e.g., care of relatives. | | | | | | | |
| professional authority | | ... embodiment of authority by dentist, whose decisions are not questioned. | | | | | | | |
| psychological needs of patient | | ... basic psychological needs required for well-being or life stability, e.g., sense of security. | | | | | | | |
| quality assessment culture | | ... implementation of measures in dental institution aiming at continuous quality improvement. | | | | | | | |
| recommendation by professional | | ... recommendation for or against dental treatment by person with (dental) professional expertise. | | | | | | | |
| recommendation of others | | ... recommendation for or against dental treatment by family or friends. | | | | | | | |
| relationship to dentist | | ... relationship between patient and dentist. | | | | | | | |
| relationship to staff | | ... relationship between patient and dental staff. | | | | | | | |
| reliability | | ... extent to which announced condition occurs. | | | | | | | |
| safety | | ... state of protection from danger or harm. | | | | | | | |
| satisfaction | | ... fulfillment of desired outcomes, indicating a high probability of the patient’s return. | | | | | | | |
| scented operatory | | ... perfuming of dental institution rooms. | | | | | | | |
| sensitive | | ... patient-individual extent of stimulus-reaction according to dental treatment, e.g., pain. | | | | | | | |
| shame | | ... patient's sense of shame when her/his teeth are seen by another person. | | | | | | | |
| signs of service areas | | ... signposting in dental institution with aim of best patient orientation. | | | | | | | |
| simplicity | | ... implementation of dental treatment with least effort. | | | | | | | |
| social class | | ... classification of patient into a category based on socio-economic characteristics. | | | | | | | |
| social competence of dentist | | ... ability of the dentist to act in a social environment. | | | | | | | |
| social competence of staff | | ... ability of the dental staff to act in a social environment. | | | | | | | |
| social environment | | ... social structure of patient, including presence of parents during dental treatment of child. | | | | | | | |
| social pressure | | ... pressure on patient by her/his social environment. | | | | | | | |
| social security | | ... security patient feels and receives from her/his social environment. | | | | | | | |
| social-self | | ... patient’s point of view within her/his social environment. | | | | | | | |
| special treatment necessary | | ... need for particular (dental) treatment. | | | | | | | |
| specialist | | ... involvement or consultation of dentist or physician specializing in particular dental/medical discipline. | | | | | | | |
| staff attire | | ... overall external impression of dental staff, e.g., dress. | | | | | | | |
| stuck in traffic | | ... patient’s delay to dental treatment appointment due to traffic conditions. | | | | | | | |
| surgery necessary | | ... that dental treatment (also) includes a surgical intervention. | | | | | | | |
| technical qualifications | | ... training status of (dental) practitioners and dental staff to use technical equipment correctly. | | | | | | | |
| teeth location | | ... position of tooth in dentition, e.g., anterior tooth in maxilla. | | | | | | | |
| time duration of session | | ... time duration of single session within dental treatment. | | | | | | | |
| time off work required | | ... need for patient to take time off from work for dental treatment appointment. | | | | | | | |
| tooth color | | ... color or discoloration of the tooth or dentition. | | | | | | | |
| treatment availability | | ... fact that a certain dental treatment can be implemented, e.g., due to technical conditions. | | | | | | | |
| treatment period | | ... time duration of dental treatment period. | | | | | | | |
| treatment success | | ... fulfillment of expectations regarding dental treatment results. | | | | | | | |
| trust in dentistry | | ... confidence from patient’s perspective in dental care. | | | | | | | |
| trust in dentists | | ... patient's confidence in dentist regarding her/his decisions and actions. | | | | | | | |
| trust in staff | | ... patient's trust in dental staff regarding their decisions and actions. | | | | | | | |
| usability | | ... feasibility of dental treatment or non-restriction by dental treatment results in patient’s everyday life. | | | | | | | |
| visiting friends/relatives in area | | ... use of dental treatment appointment to visit family and friends in surrounding area of dental institution. | | | | | | | |
| waiting room facilities | | ... furnishings in waiting area of medical facility. | | | | | | | |
| waiting time for appointment | | ... waiting time for an appointment, which results from high utilization of dentist, e.g., due to popularity. | | | | | | | |
| willingness-to-travel | | ... the patient’s willingness to accept travel-related effort (e.g., time, cost) to receive dental treatment. | | | | | | | |
