# Supplementary material for: Decision-making regarding dental treatments – What factors matter from patients’ perspective? A systematic review
Source: BMC Oral Health. 2025 Nov 25;26:289. doi: 10.1186/s12903-025-07032-9 (PMC12903421; doi:10.1186/s12903-025-07032-9)
Supplement: Supplementary file 1 — Additional file 1: A1. Guideline on literature search, selection, and analysis. A2. Search strategy. A3. PRISMA checklist. A4. SWiM checklist. A5. Search strings for databases, including hits. A6. Characteristics, factors of choice, and references of included articles (N = 233), sorted by number of identified articles per country (descending) within study designs I–V. A7. Methodological characteristics of included articles (N = 233), and search details. A8. Coding scheme, codebook, and framework, including definitions of excluded and summarized codes. A9. Code definitions. A10. Calculation of ICA and ICR. A11. Quality assessment by MMAT: study design I. A12. Quality assessment by MMAT: study design II. A13. Quality assessment by MMAT: study design III. A14. Quality assessment by MMAT: study design IV. A15. Quality assessment by MMAT: study design V. A16. MMAT assessment results description. [file 12903_2025_7032_MOESM1_ESM.zip › A9_Code_definitions.docx]

**A9.** Code definitions

| **No.** | **Code (= factor)** | **Definition:** |
| --- | --- | --- |
| **1** | academic institution | ... academic background of the dentist, dental staff, or institution, e.g., university hospital. |
| **2** | acceptance | ... the patient’s acceptance of dental treatment. |
| **3** | access barriers | ... factors influencing access to dental treatment or care. |
| **4** | adaptation with alternative | ... the dental treatment can be extended to include another (dental) treatment if necessary. |
| **5** | aesthetics | ... the patient’s appearance perceived as beautiful. |
| **6** | aftercare | ... necessity of certain behavior in private after dental treatment. |
| **7** | altruism | ... selfless behavior of a person towards at least one other person. |
| **8** | appearance | ... overall external impression of a person (dentist, staff). |
| **9** | collaboration with other medical professionals | ... cooperation of the dentist with other dentists or physicians, e.g., specialists. |
| **10** | comfort | ... condition the patient perceives as pleasant. |
| **11** | communication skills | ... skills of a dentist and dental staff concerning their verbal and non-verbal communication. |
| **12** | complexity of treatment | ... extent of dental treatment in terms of several treatment sessions. |
| **13** | complicated treatment | ... degree of severity when performing a dental treatment, e.g., invasive procedure. |
| **14** | complications | ... occurrence of unplanned events during or after dental treatment. |
| **15** | consequences | ... medical or non-medical result of dental treatment for the patient. |
| **16** | convenience | ... amenities for the patient associated with receiving dental treatment. |
| **17** | customer service | ... retention activities by the dental institution designed to ease utilization of dental treatment for the patient. |
| **18** | dentist attire | ... an overall external impression of the dentist. |
| **19** | dentist behavior | ... perceptible behavior of the dentist. |
| **20** | dentist characteristics | ... personal characteristics of the dentist, e.g., age, gender. |
| **21** | dentist of choice | ... possibility for the patient to choose between several dentists or dental institutions. |
| **22** | dentist professional skills | ... application of dental knowledge through learned methods and skills by the dentist. |
| **23** | dentist qualification | ... the dentist’s education, e.g., degree from a particular university. |
| **24** | dentist's responsiveness | ... responsibility of the dentist regarding dental treatment and her/his decisions towards a patient. |
| **25** | diagnostic care | ... necessity of diagnostic intervention. |
| **26** | documentation | ... seamless recording of patient and dental treatment data in the dental institution. |
| **27** | durability | ... duration after completing dental treatment until a new dental intervention is necessary. |
| **28** | duration of treatment | ... time required for dental treatment, including all treatment sessions. |
| **29** | easy access to modes of non-doctor treatments (e.g., drugs) | ... barrier-free access to drug treatment alternatives. |
| **30** | efficient treatment | ... dental treatment as worthwhile for the patient regarding cost-benefit ratio. |
| **31** | emergency | ... medical urgency for dental treatment. |
| **32** | emergency service availability | ... emergency medical care is available at the (dental) institution. |
| **33** | ethics | ... compliance with or consideration of ethical aspects in implementation of dental treatment. |
| **34** | experience by patient | ... the patient’s experience from previous dental treatments. |
| **35** | facilities | ... rooms and characteristics of the dental institution, e.g., decorative items, cleanliness. |
| **36** | family orientation | ... consideration of family needs or circumstances of the patient. |
| **37** | fear | ... the patient’s dental anxiety or concerns regarding dental treatments. |
| **38** | feedback culture | ... receiving patient feedback on dental treatments and its implementation aiming at process improvement. |
| **39** | flexibility | ... opportunities of adjustment regarding dental treatments by the dentist. |
| **40** | follow-up care | ... dental institution offering treatment-specific care after dental treatment. |
| **41** | for second opinion | ... another dentist is consulted by the patient to obtain a second opinion, e.g., regarding costs or invasiveness. |
| **42** | forgetfulness | ... the patient’s weakness to remember things, e.g., regarding an appointment. |
| **43** | functionality | ... restoration of natural function of dentition with dental treatment. |
| **44** | health status | ... the patient’s state of health, described by measurable or visible characteristics, e.g., degree of caries disease. |
| **45** | health-seeking behavior | ... the patient’s behavior towards dental care, e.g., utilization of prevention. |
| **46** | improve professional opportunities | ... benefits that accrue in the patient’s professional life from dental treatment. |
| **47** | instalments | ... possibility for the patient of paying dental treatment costs in staggered smaller amounts. |
| **48** | insurance | ... the patient’s membership in statutory or private health insurance. |
| **49** | intrusiveness of dentist and staff | ... unpleasant insistence of the dentist or dental staff on the patient to act against her/his original will. |
| **50** | life-course perspective | ... presumed remaining lifetime of the patient. |
| **51** | location | ... location of dental institution, e.g., city center, rural region. |
| **52** | medical equipment | ... any medical instruments and/or medical devices used for treatment. |
| **53** | medical error | ... medical malpractice decisions and treatments. |
| **54** | medical need | ... need for dental treatment diagnosed by the dentist or other medical professional. |
| **55** | medical tourism | ... the patient’s way of travelling to other (parts of) countries in search of dental treatments or dentists. |
| **56** | medicine as alternative | ... use of medication instead of dental treatment. |
| **57** | naturality | ... preservation or restoration of the natural structure of teeth or dentition. |
| **58** | oral health awareness | ... the patient’s attention to her/his oral health. |
| **59** | organization | ... processes and their coordination in the dental office. |
| **60** | other/different alternative | ... patient deciding for an alternative (dental) treatment. |
| **61** | outcome | ... health-related measurable results of dental treatment. |
| **62** | out-of-pocket payment | ... financial cost of treatment taken by the patient. |
| **63** | overall impression | ... the patient’s perception of dental institution, including organization of processes and friendly (staff) atmosphere. |
| **64** | pain | ... pain experienced by the patient due to dental disease, including pain before, during and after dental treatment. |
| **65** | patient characteristics | ... the patient’s personal characteristics, e.g., age, gender. |
| **66** | patient knowledge | ... the patient’s knowledge of dentistry and oral health, including dental treatment alternatives she/he is facing. |
| **67** | patient motivation | ... totality of the patient’s motivations that influence her/his decisions, actions, and health-behavior. |
| **68** | payment modality | ... way in which payment can be made by the patient, e.g., by credit card. |
| **69** | personalized care | ... adaptation of dental treatment to patient-specific conditions or wishes. |
| **70** | physical needs of patient | ... body functions of the patient necessary for her/his life support. |
| **71** | prevention | ... (regular) preventive dental measures taken by the patient. |
| **72** | provided information | ... given information on dental care, including (dental) treatment alternatives, by dental institution, e.g., via website. |
| **73** | quality of care | ... features of health care with the aim of achieving best possible dental treatment results. |
| **74** | quality of life | ... features of everyday life and living conditions with the aim of achieving best possible dental treatment results. |
| **75** | racism | ... attitudes, way of thinking, and acting towards persons or population groups with certain ethnic origin. |
| **76** | recommendation | ... suggestions for actions or reports of (dental) experiences from dental professionals or other people (e.g., family, friends)  regarding dental treatments. |
| **77** | referred by professional | ... referral to a dental institution or to treatment of the patient by dentist or other (dental) medical professional. |
| **78** | relationship to professional | ... type of connection and behavior towards each other between the patient and dentist or dental staff. |
| **79** | religion | ... behavior of the patient is based on principles of a religion or religious community. |
| **80** | respect | ... respectful behavior of the dentist and dental staff towards the patient and between each other. |
| **81** | risk | ... the possibility that a desired dental treatment result does not occur is accepted by the patient. |
| **82** | SARS-CoV-2 infection | ... the patient’s fear of infection with the coronavirus of the current COVID-19 pandemic (since 2019). |
| **83** | school requirement | ... routine dental examination of children that are mandatory or offered in educational institutions. |
| **84** | self-diagnosis | ... assessment of the patient's own health status and derivation of dental treatment measures by the patient her-/himself. |
| **85** | self-esteem | ... the patient’s confidence in her-/himself, expressed by self-confident appearance. |
| **86** | sickness/unwellness | ... the patient’s sickness or unwellness other than dental disease leading the patient to postpone or cancel a dental appointment. |
| **87** | social competence of professionals | ... ability of the dentist and dental staff to act in a social environment. |
| **88** | social environment | ... social structure of the patient defining her/his everyday life. |
| **89** | social isolation | ... self-imposed isolation of the patient from her/his social environment. |
| **90** | source of information | ... any sources of information about dentistry and oral health used by the patient, e.g., internet. |
| **91** | specialties in dental practice | ... concentration of dentists or other dental/medical professionals with different specializations in one dental/medical institution. |
| **92** | staff behavior | ... appropriate and friendly behavior of dental staff towards the patient. |
| **93** | staff qualification | ... training status of dental staff. |
| **94** | time management of patient | ... structured handling by the patient of her/his available time. |
| **95** | time management of professional | ... structured handling of the dentist and dental staff with available time. |
| **96** | tooth saving | ... dental measures taken by the dentist or patient to save teeth from extraction. |
| **97** | transparency | ... disclosure and traceability of processes in dental institution. |
| **98** | transportation | ... transportation of the patient to dental institution, e.g., by bus or car. |
| **99** | treatment characteristics | ... attributes and levels concerning dental treatment, e.g., due to material. |
| **100** | trust | ... the patient’s confidence in the dentist’s and dental staff’s decisions and actions. |
| **101** | understandable information | ... communication by dentist and dental staff in a way that is understandable to the patient, e.g., in lay language. |
