# Supplementary material for: Decision-making regarding dental treatments – What factors matter from patients’ perspective? A systematic review
Source: BMC Oral Health. 2025 Nov 25;26:289. doi: 10.1186/s12903-025-07032-9 (PMC12903421; doi:10.1186/s12903-025-07032-9)
Supplement: Supplementary file 1 — Additional file 1: A1. Guideline on literature search, selection, and analysis. A2. Search strategy. A3. PRISMA checklist. A4. SWiM checklist. A5. Search strings for databases, including hits. A6. Characteristics, factors of choice, and references of included articles (N = 233), sorted by number of identified articles per country (descending) within study designs I–V. A7. Methodological characteristics of included articles (N = 233), and search details. A8. Coding scheme, codebook, and framework, including definitions of excluded and summarized codes. A9. Code definitions. A10. Calculation of ICA and ICR. A11. Quality assessment by MMAT: study design I. A12. Quality assessment by MMAT: study design II. A13. Quality assessment by MMAT: study design III. A14. Quality assessment by MMAT: study design IV. A15. Quality assessment by MMAT: study design V. A16. MMAT assessment results description. [file 12903_2025_7032_MOESM1_ESM.zip › A11_Quality_assessment_by_MMAT_study_design_I.docx]

**A11.** Quality assessment by MMAT: study design I

| **Quality assessment by Mixed Methods Appraisal Tool (MMAT): study design I – Qualitative studies** | | | | | | | | | | |
| --- | --- | --- | --- | --- | --- | --- | --- | --- | --- | --- |
| Questions to answer:  **S1. Are there clear research questions?**  **S2. Do the collected data allow to address the research questions?**  **1.1. Is the qualitative approach appropriate to answer the research question?** *E.g., grounded theory approach for development of a theory, ethnography for human cultures/society*  **1.2. Are the qualitative data collection methods adequate to address the research question?** *Judge method of data collection (in depth/group interviews, observations) and form of data (tape recording, video, photo); clear justifications needed if collection methods modified during study*  **1.3. Are the findings adequately derived from the data?** *Appropriate data analysis method, e.g., open/axial/selective coding for grounded theory and within-/cross-case analysis for case studies*  **1.4. Is the interpretation of results sufficiently substantiated by data?** *E.g., quotes justify themes*  **1.5. Is there coherence between qualitative data sources, collection, analysis, and interpretation?** *There is a clear link between sources/collection/analysis/interpretation* | | | | | | | | | | |
| **No.** | **Reference^1^: author (year)** | **S1. clear research questions** | **S2. data addresses research questions** | **1.1. appropriate qualitative approach** | **1.2. adequate collection methods** | **1.3. findings adequately derived** | **1.4. sufficiently interpretation of results** | **1.5. coherence between data sources, collection, analysis, and interpretation** | **Number of points** | **Quality score (points)** |
| I.1 | Al-Moghrabi et al. (2019) | yes | yes | 1 | 1 | 0 | 1 | 1 | 4 | 0.8 (****) |
| I.2 | Borreani et al. (2010) | yes | yes | 1 | 1 | 0 | 1 | 0 | 3 | 0.6 (***) |
| I.3 | Ellis et al. (2011) | yes | yes | 0 | 1 | 1 | 1 | 1 | 4 | 0.8 (****) |
| I.4 | Exley et al. (2012) | yes | yes | 1 | 1 | 1 | 0 | 0 | 3 | 0.6 (***) |
| I.5 | Grey et al. (2013) | yes | yes | 1 | 1 | 0 | 1 | 0 | 3 | 0.6 (***) |
| I.6 | Hanefeld et al. (2015) | yes | yes | 1 | 1 | 1 | 1 | 0 | 4 | 0.8 (****) |
| I.7 | Kashbour et al. (2018) | yes | yes | 1 | 1 | 1 | 1 | 1 | 5 | 1.0 (*****) |
| I.8 | Ke et al. (2013) | no | 0 | 0 | 0 | 0 | 0 | 0 | 0 | none |
| I.9 | Scott et al. (2009) | yes | yes | 1 | 1 | 1 | 1 | 1 | 5 | 1.0 (*****) |
| I.10 | Serban et al. (2019) | yes | yes | 1 | 1 | 1 | 1 | 0 | 4 | 0.8 (****) |
| I.11 | Thompson et al. (2020) | yes | yes | 1 | 1 | 0 | 1 | 1 | 4 | 0.8 (****) |
| I.12 | van der Zande et al. (2021) | yes | yes | 1 | 1 | 1 | 1 | 1 | 5 | 1.0 (*****) |
| I.13 | Bohn et al. (2018) | yes | yes | 1 | 1 | 0 | 1 | 1 | 4 | 0.8 (****) |
| I.14 | Cohen et al. (2007) | yes | yes | 0 | 0 | 0 | 0 | 1 | 1 | 0.2 (*) |
| I.15 | Dodd et al. (2014) | yes | yes | 0 | 1 | 0 | 1 | 0 | 2 | 0.4 (**) |
| I.16 | Gatten et al. (2011) | yes | yes | 1 | 1 | 1 | 0 | 1 | 4 | 0.8 (****) |
| I.17 | Hoeft et al. (2011) | yes | yes | 1 | 0 | 1 | 1 | 1 | 4 | 0.8 (****) |
| I.18 | Horton et al. (2009) | yes | yes | 1 | 1 | 1 | 1 | 0 | 4 | 0.8 (****) |
| I.19 | Siegel et al. (2012) | yes | yes | 1 | 1 | 1 | 1 | 1 | 5 | 1.0 (*****) |
| I.20 | Brown et al. (2020) | yes | yes | 1 | 0 | 1 | 1 | 0 | 3 | 0.6 (***) |
| I.21 | Nogueria et al. (2019) | yes | yes | 1 | 1 | 1 | 1 | 1 | 5 | 1.0 (*****) |
| I.22 | Mostajer Haqiqi et al. (2016) | yes | yes | 1 | 1 | 1 | 1 | 1 | 5 | 1.0 (*****) |
| I.23 | Atieh et al. (2016) | yes | yes | 1 | 1 | 1 | 1 | 1 | 5 | 1.0 (*****) |
| I.24 | Giddings et al. (2008) | yes | yes | 1 | 1 | 1 | 1 | 1 | 5 | 1.0 (*****) |
| I.25 | Gregory et al. (2012) | yes | yes | 1 | 1 | 1 | 1 | 1 | 5 | 1.0 (*****) |
| I.26 | McKenzie-Green et al. (2009) | yes | yes | 1 | 1 | 0 | 1 | 1 | 4 | 0.8 (****) |
| I.27 | Osman et al. (2014) | no | 0 | 0 | 0 | 0 | 0 | 0 | 0 | none |
| I.28 | Sussex et al. (2010) | yes | yes | 1 | 1 | 1 | 1 | 0 | 4 | 0.8 (****) |
| I.29 | Abrahamsson et al. (2017) | yes | yes | 1 | 1 | 1 | 1 | 1 | 5 | 1.0 (*****) |
| I.30 | Johannsen et al. (2012) | yes | yes | 1 | 1 | 1 | 1 | 0 | 4 | 0.8 (****) |
| I.31 | Narby et al. (2012) | yes | yes | 1 | 1 | 1 | 1 | 1 | 5 | 1.0 (*****) |
| I.32 | Ostberg et al. (2013) | yes | yes | 1 | 1 | 1 | 1 | 1 | 5 | 1.0 (*****) |
| I.33 | Canuto et al. (2018) | yes | yes | 1 | 1 | 1 | 1 | 1 | 5 | 1.0 (*****) |
| I.34 | Slack-Smith et al. (2010) | yes | yes | 1 | 1 | 0 | 1 | 1 | 4 | 0.8 (****) |
| I.35 | Azhar et al. (2018) | yes | no | 0 | 0 | 0 | 0 | 0 | 0 | none |
| I.36 | Cronin et al. (2009) | yes | yes | 1 | 1 | 1 | 1 | 1 | 5 | 1.0 (*****) |
| I.37 | Niesten et al. (2013) | yes | yes | 1 | 1 | 1 | 1 | 1 | 5 | 1.0 (*****) |
| I.38 | Vanobbergen (2007) | yes | yes | 1 | 1 | 1 | 1 | 1 | 5 | 1.0 (*****) |
| I.39 | Mittal et al. (2019) | yes | yes | 1 | 1 | 1 | 1 | 1 | 5 | 1.0 (*****) |
| I.40 | Munira Hernandez-Santos et al. (2021) | no | 0 | 0 | 0 | 0 | 0 | 0 | 0 | none |
| I.41 | Naidu (2012) | no | 0 | 0 | 0 | 0 | 0 | 0 | 0 | none |
| I.42 | Abd Mutalib et al. (2017) | yes | yes | 1 | 0 | 1 | 1 | 1 | 4 | 0.8 (****) |
| **Legend:** ^1^ order of references according to Table A6 | | | | | | | | | | |
